# Cobalt photocatalysed carbonylation reactions in continuous flow

**DOI:** 10.1039/d6cc03757g

**Published:** 2026-08-31

**Authors:** Mónica Martínez-Aguirre, Rowan M. Bailey, Bernd Schaefer, Mark R. Crimmin, Philip W. Miller

**Affiliations:** a Department of Chemistry, Imperial College London, Molecular Sciences Research Hub, White City Campus, Wood Lane London W12 OBZ UK philip.miller@imperial.ac.uk; b BASF, Ludwigshafen am Rhein, Rhineland-Palatinate Germany

## Abstract

A cobalt photocatalysed carbonylation reaction has been developed and applied in continuous flow. Dicobalt octacarbonyl, [Co_2_(CO)_8_], serves both as a catalyst and as a carbon monoxide surrogate for the synthesis of amides and esters. The process is technically straightforward, has been applied to pharamceutical derviatives and demonstrated on multi-gram scale.

## Abstract 2

A cobalt photocatalysed carbonylation reaction has been developed and applied in continuous flow.

## Graphical abstract

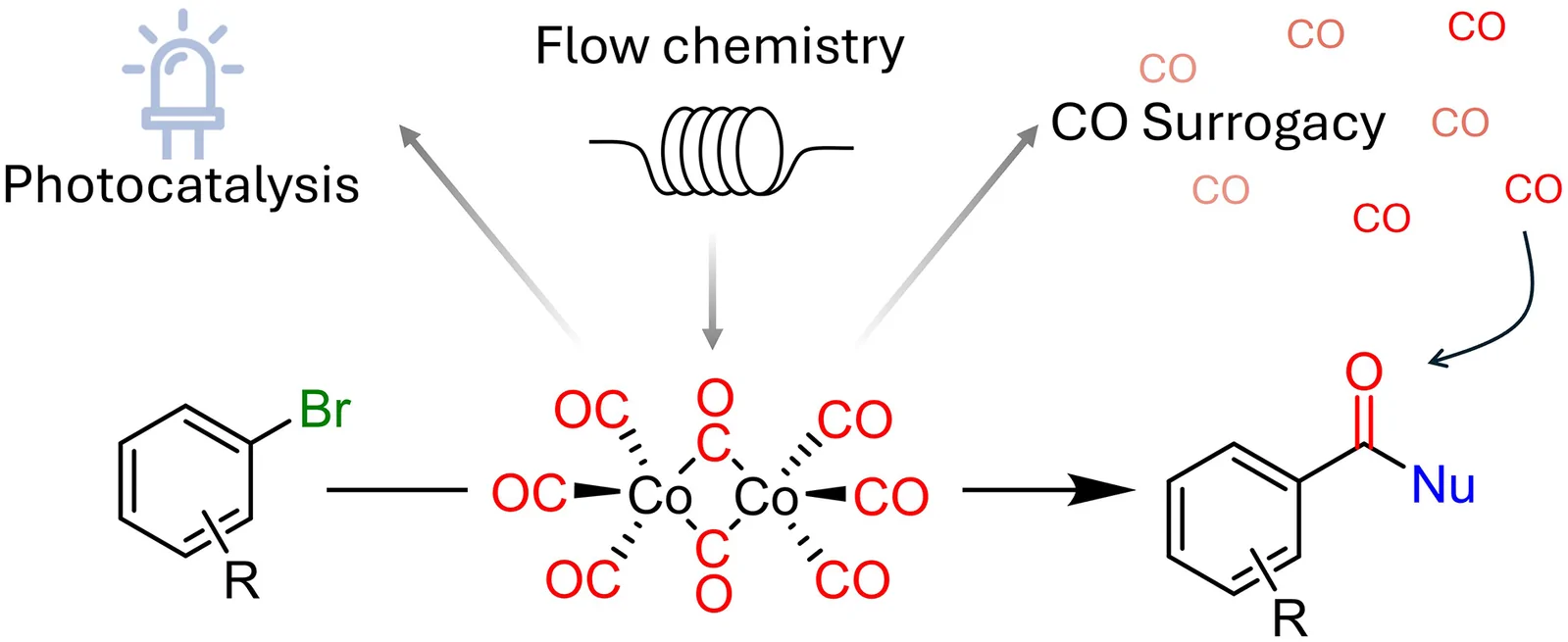

Amides are among the most prevalent functional groups in active pharmaceutical ingredients and agrochemicals.^1–3^ Typically, amides are prepared by reaction of acyl chlorides with amines or through the coupling of carboxylic acids using stoichiometric activating agents. The drawbacks of these routes, including toxic reagents, side reactivity and low atom economy, can limit their application (Fig. 1).^4–6^ Palladium catalysed carbonylation reactions offer a viable alternative and atom-economical route to access carbonyl-containing functional groups.^7–9^ In some instances these methods have been applied on scale (*e.g.* ibuprofen).^10,11^ However, expensive Pd and specialised ligands, extended reaction times at elevated temperatures and pressures, and restricted functional group tolerance have limited industrial deployment (Fig. 1c).^12,13^ Furthermore, the handling of toxic and flammable carbon monoxide gas necessitates specialised equipment and stringent safety protocols.

**Fig. 1:**
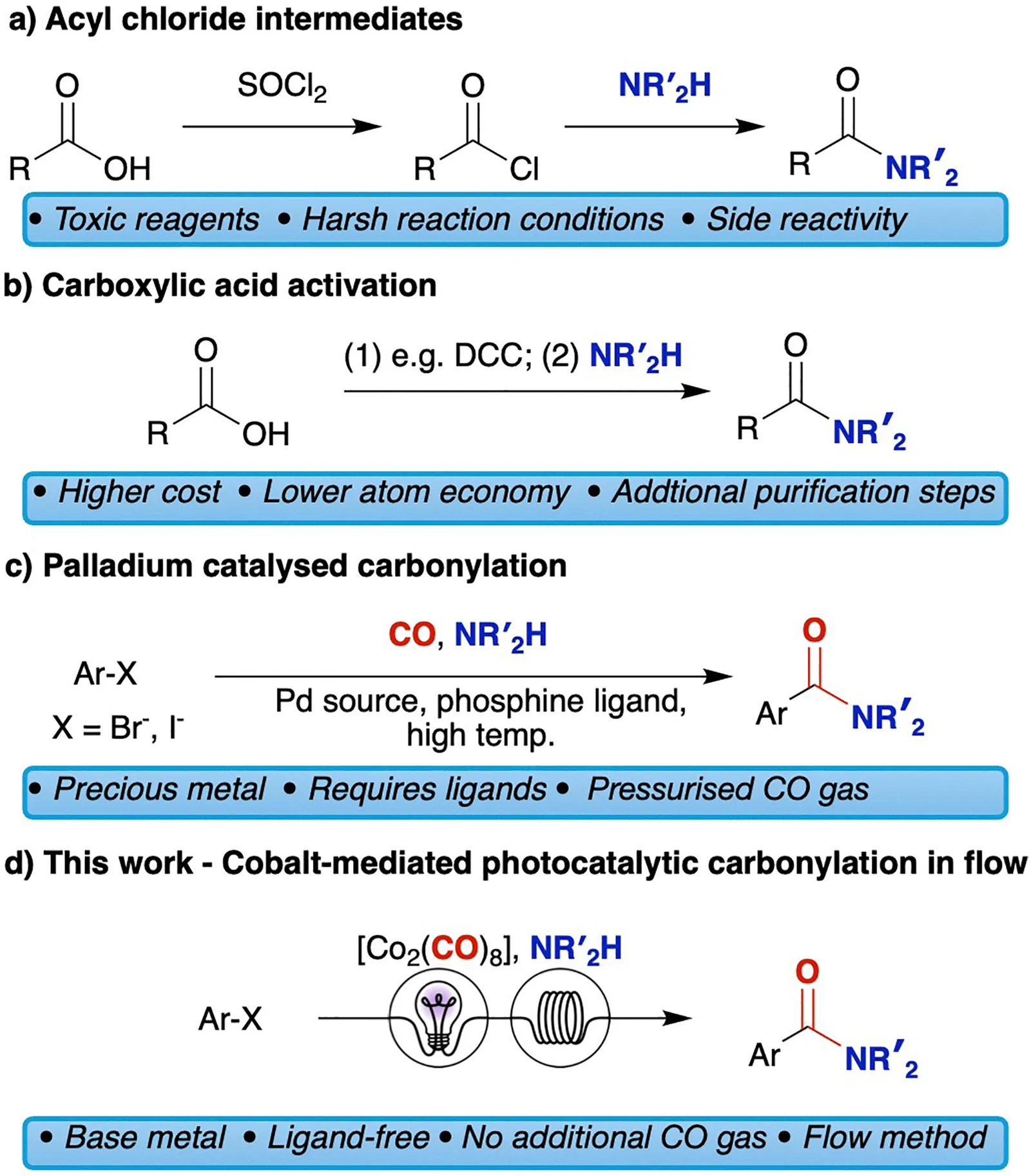
(a and b) Typical routes for amide synthesis methods and their characteristics. DCC = dicyclohexylcarbodiimide. (c) Pd catalysed carbonylations for amide formation. (d) Cobalt mediated photocatalytic carbonylation used in this approach.

Photocatalysis has recently shown potential to address some of the limitations of conventional Pd-catalysed carbonylations.^14^ The use of narrow-wavelength LEDs has enabled the development of new photocatalytic carbonylation methods employing more sustainable transition metals and/or organophotocatalysts.^15–18^ Compared to traditional Pd-based approaches, these photochemical methods can operate under lower CO pressures, access distinct reactivity and catalytic pathways, boasting enhanced chemoselectivity and broad substrate scope in select cases.^14,19–21^

The use of CO gas presents significant practical challenges as reactions typically require pressurisation, specialised equipment, and technical expertise to ensure safe handling. To address these limitations, CO surrogates—defined as compounds that release carbon monoxide upon activation—have been widely explored in laboratory settings.^22,23^ Metal carbonyl complexes have been extensively employed for this purpose.^24–30^ More recently, the *in situ* use of a single compound as both the CO source and catalytic species has been demonstrated with [Co_2_(CO)_8_] and [Mn_2_(CO)_10_] for photocatalytic synthesis of amides and esters.^31–33^ The relatively low cost of cobalt and manganese, combined with the absence of ancillary ligands and the operational simplicity of such systems, renders these photocarbonylation methods attractive for both reaction discovery and small-scale synthesis. Despite this, examples using [Co_2_(CO)_8_] are limited by excessive catalyst loadings (>100 mol%) or addition of alternative surrogates to furnish significant yields.

Flow chemistry is widely regarded as a highly effective approach for scaling photochemical reactions, owing to its enhanced surface-area-to-volume ratios, improved light penetration through narrow reactor channels (typically ∼1 mm), and well-defined mixing profiles.^34–37^ Whilst several photocarbonylation reactions have been previously translated to flow,^19,38,39^ we envisioned that combining a CO surrogate system with photocarbonylation in flow would circumvent the need for CO handling. Herein, we report a homogeneous and operationally accessible flow-photocatalysed carbonylation process that employs [Co_2_(CO)_8_] as a dual CO surrogate and catalyst, enabling the efficient synthesis of amides and esters from aryl bromides.

The photocatalysed carbonylation of 4-bromoanisole and piperidine using [Co_2_(CO)_8_] was selected as a model reaction. Preliminary testing under batch conditions with 5 mol% [Co_2_(CO)_8_] using an Asynt Illumin8 photoreactor and tetramethylpiperidine (TMP) base yielded ∼40% of amide after 48 h irradiation at 365 nm which equated to quantitative consumption of CO (see SI). Unfortunately, this reaction mixture was heterogeneous owing to the formation of TMP hydrobromide salts which would hinder flow studies. Recently we discovered that the selection of base is crucial to both the rate of reaction and the solubility of hydrobromide salt byproducts. Replacing TMP with the organic superbase DBU generated homogeneous reaction mixtures and markedly improved reaction kinetics.^21^ Using a rudimentary flow set-up with a coil of FEP tubing placed inside the Illumin8 photoreactor, preliminary experiments using higher quantities of [Co_2_(CO)_8_] (12.5 mol%, 1 equiv. in CO) and DBU as base gave amide yields of 48%, at residence times (*t*_R_) of 60 min (SI, table S2).

Encouraged by these preliminary flow results, a modified set-up using a Vapourtec UV-150 flow photoreactor, equipped with a 60 W 365 nm LED was used to investigate the effects of residence time, catalyst/surrogate concentration, solvent choice, substrate concentration and light source. We found that it was essential to employ two input streams, one containing [Co_2_(CO)_8_] dissolved in a *t*-amyl-OH/THF solvent mixture, and the other containing the organic reagents (Fig. 2). Mixing the two solutions in flow minimised the risk of initial CO dissolution from reaction of DBU with [Co_2_(CO)_8_]. Using stoichiometric amounts of CO *i.e.* [Co_2_(CO)_8_] loadings of 12.5 mol% and varying the residence time between 15 to 60 min demonstrated a yield of 56% with complete conversion of the substrate. Increasing catalyst loading from 12.5 mol% to 16 mol% (1.3 equiv. of CO) showed a ∼10% improvement to amide yield at 60- and 100 min residence times, affording a maximum yield of 70%.

**Fig. 2:**
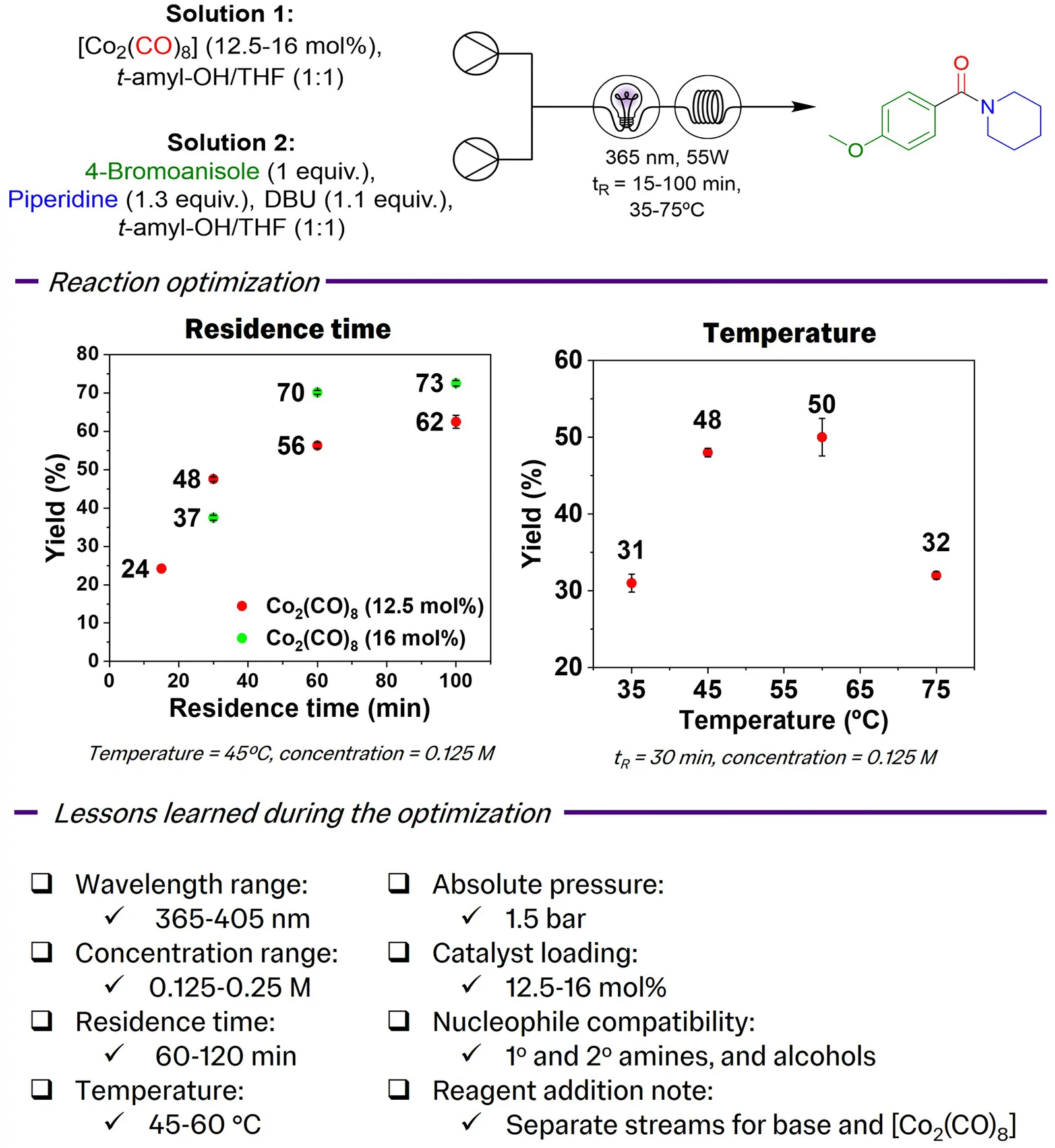
Schematic of model photocarbonylation flow reaction of 4-bromoanisole and piperidine (top); impact of changing residence times and temperature under flow conditions (middle); lessons learned during the optimization process (bottom).

The effect of substrate concentration on the reaction was marked, doubling the concentration from 0.25 M to 0.5 M resulted in a dramatically depleted yield from 59% to 12% (SI Fig. S5). We anticipate that this is due to the high absorptivity of the catalyst which can obstruct the amount of light received beyond the reaction surface. The impact of temperature on the reaction was more complex. At a residence time of *t*_R_ = 30 minutes it was found that optimal yields were obtained between 45–60 °C. Temperatures above and below this range resulted in poorer yields. This result was unexpected as previous batch tests had shown optimal reactivity at higher temperatures (>65 °C). Notably, at higher temperatures, gas segments were present. This may suggest a reduction to CO solubility, or degradation of the surrogate species.

Using either 12.5 or 16 mol% of [Co_2_(CO)_8_] consistently resulted in quantitative conversion of 4-bromoanisole, suggesting that at a concentration of 0.125 M, the reaction was not light-limited (*Φ*_Vapourtec_ = 2.80 × 10^−5^ mol s^−1^, see SI for detailed actinometry experiments). However, yields of amide were limited to 56% and 70% respectively under optimised conditions. Given the mass balance discrepancy, it was suspected that amide yield was limited by a competitive reaction which generated volatile byproducts. To investigate this further, 4-fluorobromobenzene was used to study the reaction *via*^19^F NMR spectroscopy.

Under optimized reaction conditions (0.125 M of 4-bromofluorobenzene, 60 minutes residence time, 45 °C and 15 mol% of [Co_2_(CO)_8_]), the photocarbonylation of 4-fluorobromobenzene gave similar starting material conversions and yields of amide (68–70%), by quantitative ^19^F NMR spectroscopy *versus* trifluorotoluene internal standard. The major side product (∼25%) was identified as fluorobenzene (SI Fig. S6) confirming that a competitive hydrodebromination was occurring. Hydrodebromination products are common in photocatalytic reactions,^40–43^ and have been previously reported using the [Mn_2_(CO)_10_] surrogate in similar reactions.^33^ The high cobalt concentration and the lower relative availability of CO likely favour this side reactivity.

Changing the light source to longer wavelength 405 nm did show reduced fluorobenzene formation to 10% but lowered reaction rates, requiring an increased residence times of 120 minutes to observe similar amide formation (70%, SI table S11). In this instance, starting material was still visible post-reaction. Extension of residence times above 120 minutes did not show improved reactivity.

The source of hydrogen for the hydrodehalogenation reaction was investigated using ^2^D-piperidine in place of ^1^H-piperidine. Using ^2^D-NMR spectroscopy, there was no evidence that the hydrogen/deuterium was exchanged *via* piperidine, althought it is highly likely that this experiment is complicated by *in situ* scrambling and exchange with protic solvent *t*-amyl-OH. A solvent screen was conducted to determine whether displacement of protic *t*-amyl-OH could reduce competitive side-reactivity. Although the solvent choice did have an impact on reaction selectivity, *t*-amyl-OH/THF (1/1 volume) was ultimately determined as the optimal solvent system based on solubility and yield (SI, Table S10).

The photocarbonylation of 2- and 4-fluoro-bromoarenes with a selection of amines and alcohols were investigated in the flow set-up under optimised conditions (Fig. 3). Amide yields of 50–71% were obtained for a range of nucleophiles. Impressively, sterically challenging *tert*-butylamine was reactive under the reaction conditions, albeit at lower yields. Quantitative consumption of the aryl bromide starting materials was observed for all amine nucleophiles except tert-butylamine. This suggested that the hydrodebromination pathway was again largely limiting the amide yields, however, in the case of *tert*-butylamine the steric hinderance may also impede catalysis.

**Fig. 3:**
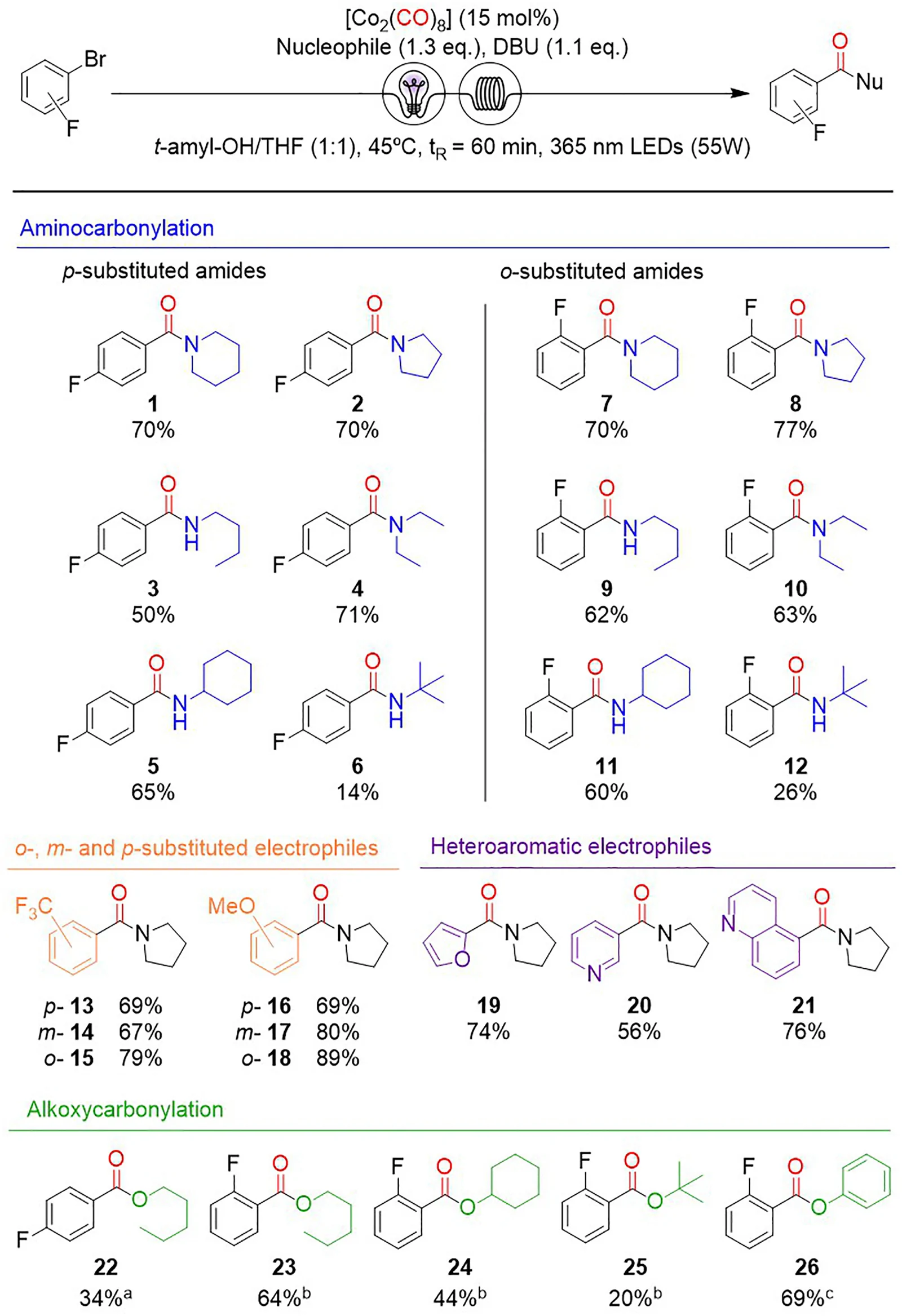
Optimised reaction conditions (top); Scope of amino/alkoxycarbonylation reactions in CO free [Co_2_(CO)_8_] mediated conditions (bottom). Q-NMR yields calculated from the ^19^F NMR and ^1^H NMR spectra (for more information, see SI). ^*a*^2 eq. DBU, *t*_R_ = 2 h, 45 °C. ^*b*^2 eq. DBU, *t*_R_ = 2 h, 60 °C, ^*c*^2 eq. DBU, *t*_R_ = 1 h, 45 °C.

Substitution at the *ortho*-, *meta*- and *para*-positions was explored using both electron-donating (EDGs) and electron-withdrawing (EWDs) groups. While substrates bearing EDGs generally afforded higher yields than those containing EWGs, the most pronounced trend was the positional effect, with *ortho*-substituted electrophiles consistently providing the highest yields (79–89%). Complete consumption of the starting material was observed in all cases, suggesting that the *ortho*-substituted substrates supress hydrodehalogenation competing reactivity. The reaction was also amenable to reaction of heteroaromatic electrophile substrates, giving amide yields ranging from 56 to 76%.

Successful alkoxycarbonylation of 2- and 4-substituted fluorobromobenzene gave ester yields between 20–69% with modified reaction conditions. A significant difference in reactivity was observed between the 2- and 4-fluorobromobenzene substrates using pentanol. Across many substrates tested, a high level of CO ligand incorporation from [Co_2_(CO)_8_] was observed, ranging from 3–6 equivalents of CO per mole of [Co_2_(CO)_8_]. The yield of amides or esters under the optimised conditions appears to be largely dependent on the rate of hydrodebromination and the nucleophilicity of the amine or alcohol, except where there are exceptionally steric nucleophiles.

Reactions were monitored continuously for extended periods (3–6 h) to assess scale-up potential. During testing, no evidence of fouling or blockages were observed, and gram scale quantities of carbonylated products were collected. Following this, an acid wash and Zaiput membrane separator were integrated into the reactor set-up (Fig. 4, top), enabling in-line separation of organic amide products from unreacted amines, DBU salts and cobalt-derived species. Residual solvents and undesired dehalogenated products were readily removed *in vacuo* from the organic stream. Five amide products were isolated in good yields ranging 50–78% and in high purities 90–95% using this reactor set up. Production rates of between 0.3–0.5 g h^−1^ were obtained (Fig. 4, bottom) enabling simple isolation of multigram amides. Medicinally relevant examples of a simplified Olaparib fragment (cancer therapy drug, 28) and Moclobemide (antidepressant, 29) were included in scaled experiments to evidence the efficacy of this process and purification method. Due to increased complexity of these molecules, additional purification by column chromatography was necessary to obtain pure 28 and 29, as starting materials were detected in products streams. We expect that tailored optimisation of these reactions would afford improved isolated yields.

**Fig. 4:**
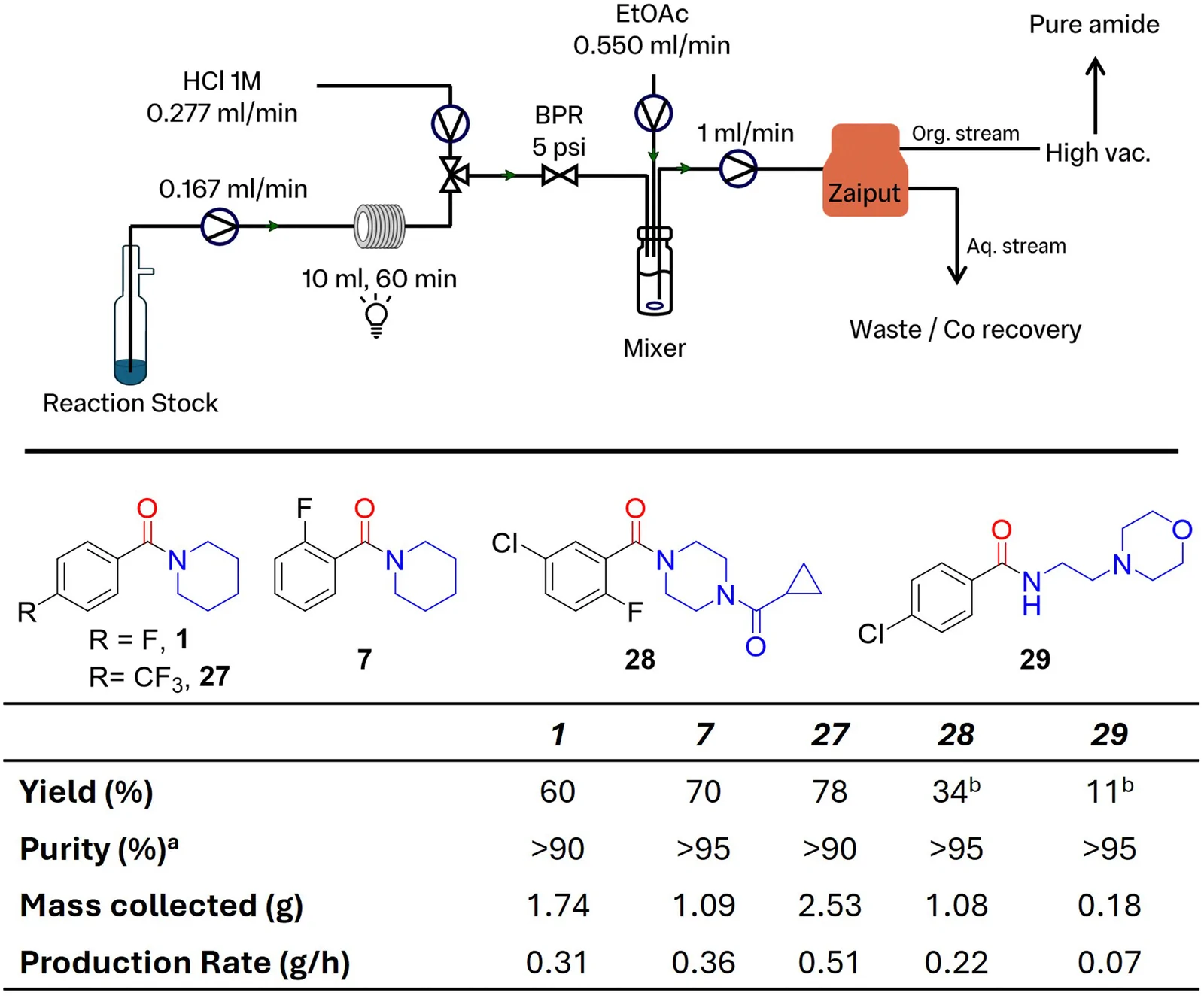
(top) Reactor set up for scope assessment and scale up [Co_2_(CO)_8_] catalysed photoaminocarbonylation. BPR = Back pressure regulator. (bottom) Example scale up products for photoaminocarbonylation reactions. Production rates are presented based on a 10 mL reaction and *t*_R_ = 1 h. ^*a*^Purity analysed by standard ^1^H NMR, from the relative integration of the product *vs.* any detectable impurity signals present in the spectra. ^*b*^Isolated yield after chromatographic column purification.

In conclusion, a cobalt mediated photocatalysed carbonylation reaction, using [Co_2_(CO)_8_] as both catalyst and CO source, has been successfully optimised and translated to flow. The set-up is technically straightforward, requiring no *ex situ* CO gas addition. The process is robust, showing no signs of fouling or blockage when operated continuously for several hours. Introduction of a membrane separator to the set up enabled in-line purification. In combination, this provided access to amide products in gram scale quantities, demonstrating the potential of the system for scale-up. Whilst this carbonylation method does exhibit a competitive side reaction that limits yields at present, the operational simplicity of the system is still, nevertheless, attractive. Circumventing the need for CO gas handling equipment is advantageous for both safety and cost considerations for smaller scale processes and we anticipate that this system will be attractive for lab-scale preparation of carbonyl containing compounds.

## Author contributions

M. Martínez-Aguirre: conceptualization, formal analysis, investigation, methodology, writing. R. M. Bailey: conceptualization, formal analysis, methodology, writing. B. Schaefer: supervision, funding acquisition. M. R. Crimmin: supervision, writing. P. W. Miller: conceptualization, supervision, formal analysis, funding acquisition, writing.

## Conflicts of interest

The authors are named inventors on a provisional European patent application EP 25187255.2 covering the preparation of amides and esters by photo-induced carbonylation.

## Supplementary Material

CC-062-D6CC03757G-s001

## Data Availability

The data supporting this article have been included as part of the supplementary information (SI). Supplementary information is available. See DOI: https://doi.org/10.1039/d6cc03757g.
