# Supplementary material for: Cobalt photocatalysed carbonylation reactions in continuous flow
Source: Chem Commun (Camb). 2026 Aug 31;62(77):19306–10. doi: 10.1039/d6cc03757g (PMC13551312; doi:10.1039/d6cc03757g)
Supplement: Supplementary file 1 [file CC-062-D6CC03757G-s001.pdf]

Supplementary Information (SI) for ChemComm.
This journal is © The Royal Society of Chemistry 2026

Supporting Information

Cobalt Photocatalysed Carbonylation Reactions in Continuous Flow

Mónica Martínez-Aguirre,[a] Rowan M. Bailey,[a] Bernd Schaefer,[b] Mark R. Crimmin,[a] Philip W. Miller[a]*

[a]Department of Chemistry, Imperial College London, Molecular Sciences Research Hub, White City Campus, Wood Lane, London, UK, W12 0BZ

[b]BASF, Ludwigshafen am Rhein, Rhineland-Palatinate, Germany

Table of Contents

1. General methods ... 3

GC Quantification ... 4

2. Preliminary tests ... 5

3. Reaction optimization ... 9

3.1. Residence time screening at different catalyst loadings ... 9

3.2. Temperature screening ... 10

3.3. Concentration screening ... 10

4. Selectivity optimization ... 12

4.1. General procedure ... 12

4.2. Catalyst loading ... 13

4.3. Piperidine and DBU loading ... 13

4.4. Solvent screening in flow ... 14

4.5. Variation of wavelength of light ... 14

4.6. Control reactions ... 15

5. ^{2}H NMR spectra ... 16

6. Reaction scope ... 17

6.1. General procedure for aminocarbonylation reactions ... 17

6.2. General procedure for alkoxycarbonylation reactions ... 29

7. Scale-up and in-line extraction in flow ... 39

8. Actinometry ... 47

9. Bibliography ... 49

1. General methods

All reagents were used as received without further purification, except for amines which were distilled prior to use. Solvents were acquired in dry form over activated alumina from a solvent purification system (SPS) and degassed before use. Reagents and solvents were obtained from Sigma Aldrich, Tokyo Chemical Industry (TCI) or Fluorochem. Dicobalt octacarbonyl [$Co_2(CO)_8$] was stored and handled inside a dinitrogen filled glovebox. Unless otherwise stated all preparations were performed using standard Schlenk line and glovebox techniques under inert atmosphere of dinitrogen. Quantitative GC analysis was recorded in an Agilent Technologies 7820A GC instrument equipped with an FID detector and an HP-5 column. Helium was used as a carrier gas and n-dodecane as the internal standard. For details on the method, see GC Quantification. Mass spectrometry was done in a Waters LCT Premier (ES-ToF) with a ACQITY i-Class system.

^{1}H and ^{19}F NMR spectra were obtained on a Bruker Av-400 spectrometer at room temperature using $CDCl_3$ and DMSO-d_6 as solvents. ^{1}H NMR chemical shifts (d) were referenced to internal proton solvent resonances, while ^{19}F NMR chemical shifts were referenced to $CFCl_3$. Chemical shifts (δ) are presented in parts per million (ppm) relative to residual solvent peaks and coupling constants (J) are given in Hz. A relaxation delay of 55 s was used for quantitative ^{19}F NMR integration. NMR data was processed using the MestReNova 14 software. Known compounds were identified by comparing the ^{1}H NMR and ^{19}F NMR spectroscopic data to the information available in the literature.

All batch photocatalytic reactions were carried out in an eight well Asynt LightSyn Illumin8 parallel photoreactor equipped with 8x10W 365 nm LEDs. A preliminary flow study was conducted using a coil of 1/16” OD (1mm ID) FEP tubing (part number: JOURJR-T-6806-M10) placed inside Asynt LightSyn Illumin8 reactor, all other flow reactions were performed in a Vapourtec UV-150 photochemical reactor, equipped with a 60 W 365 nm LED. Vapourtec peristaltic pumps and Apparatus Pump 11 Elite Syringe Pumps with SGE Gastight Syringes were used to infuse reagents. Other flow fittings include switch valves (part number: IDEX P-783), T-mixer (part number: IDEX P-712), Back Pressure Regulator Assembly 5psi (IDEX P-790), and Quick Connect Female Luer Adapters (IDEX P-659).

GC Quantification

Table S1. GC Method Details

Temperature (°C)	Hold (min)	Ramp (°C/min)
50	1	2
80	0	25
105	4	25
300	4	0

Total run time = 28.8 min, Injection volume = 1 μL, Column = GC HP-5 Column.

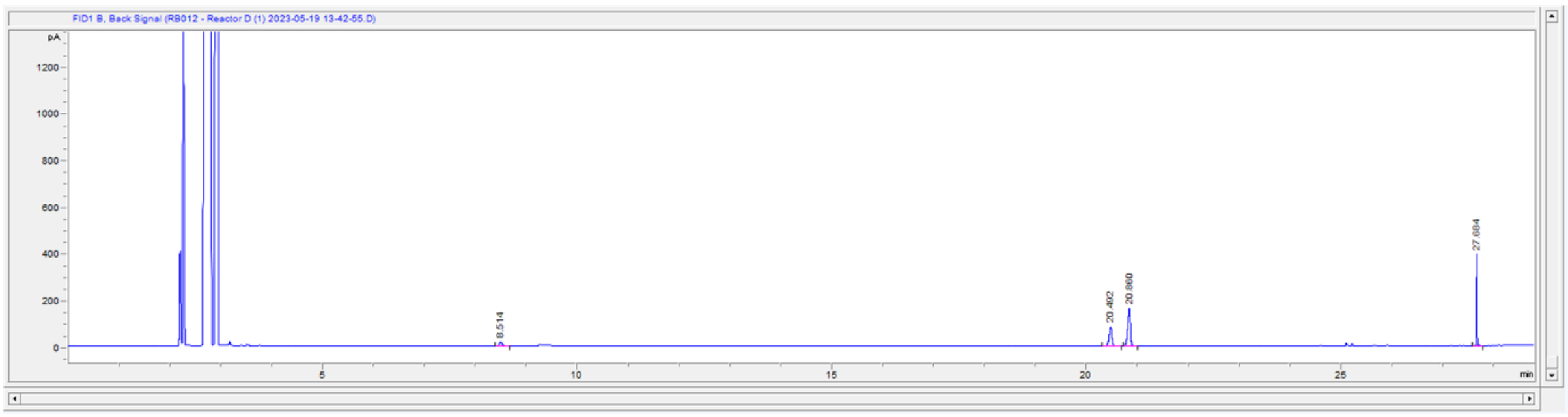

Figure S1. Example Trace: 20.492 min = 4-bromoanisole, 20.860 min = IS (n-dodecane), 27.684 min = Product Amide

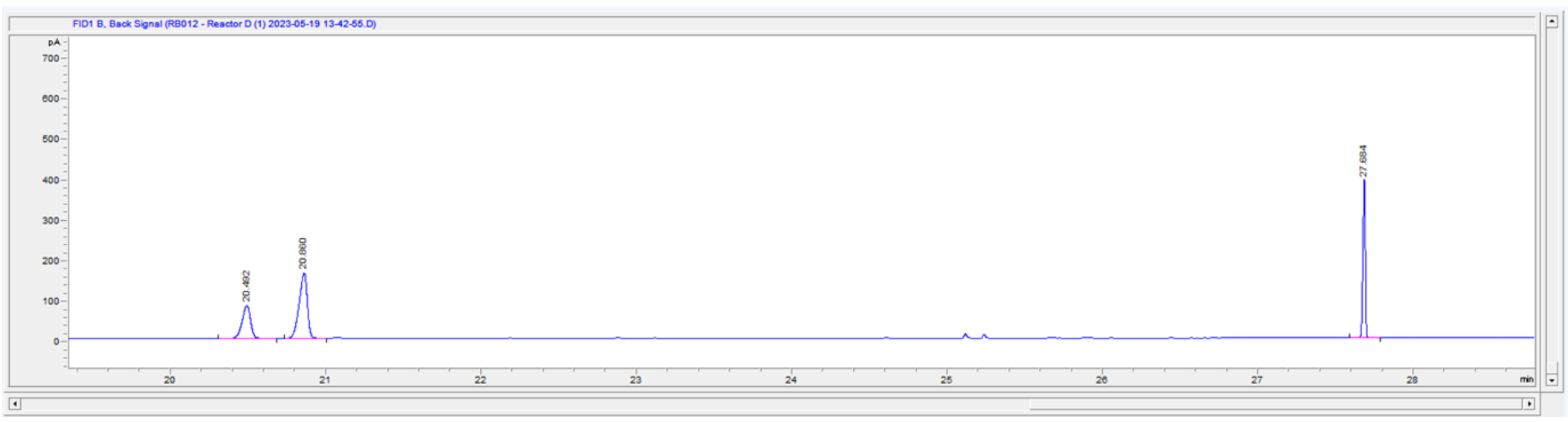

Figure S2. Zoomed GC trace

2. Preliminary tests

Preliminary tests were conducted using the Asynt Illumin8 reaction (Figure S3). Inside a glovebox, each vial was charged with 17.1 mg of [$Co_2(CO)_8$] (0.05 mmol, 0.05 equiv.), 125 µl of 4-bromoanisole (1 mmol, 1 equiv.), 128 µl of piperidine (1.3 mmol, 1.3 equiv.), 164 µl of DBU (1.1 mmol, 1.1 equiv.), and *t*-amyl-OH as solvent to a total volume of 2 ml. The reactor was sealed in a nitrogen atmosphere and irradiated under stirring for 48 hours at 45 ºC. After that time, an aliquot was taken from each reaction vial and the yield was analysed by GC using n-dodecane as the internal standard.

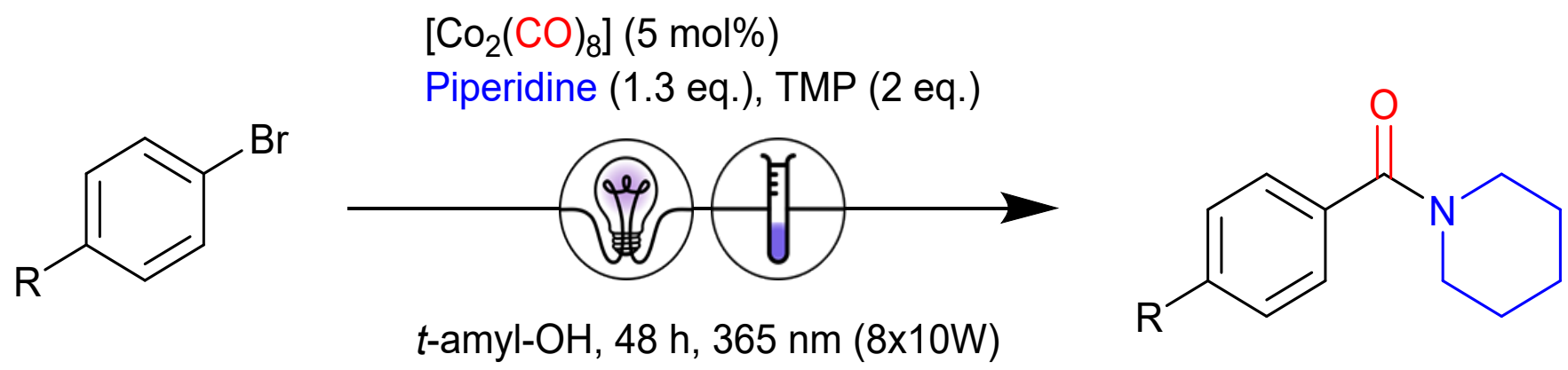

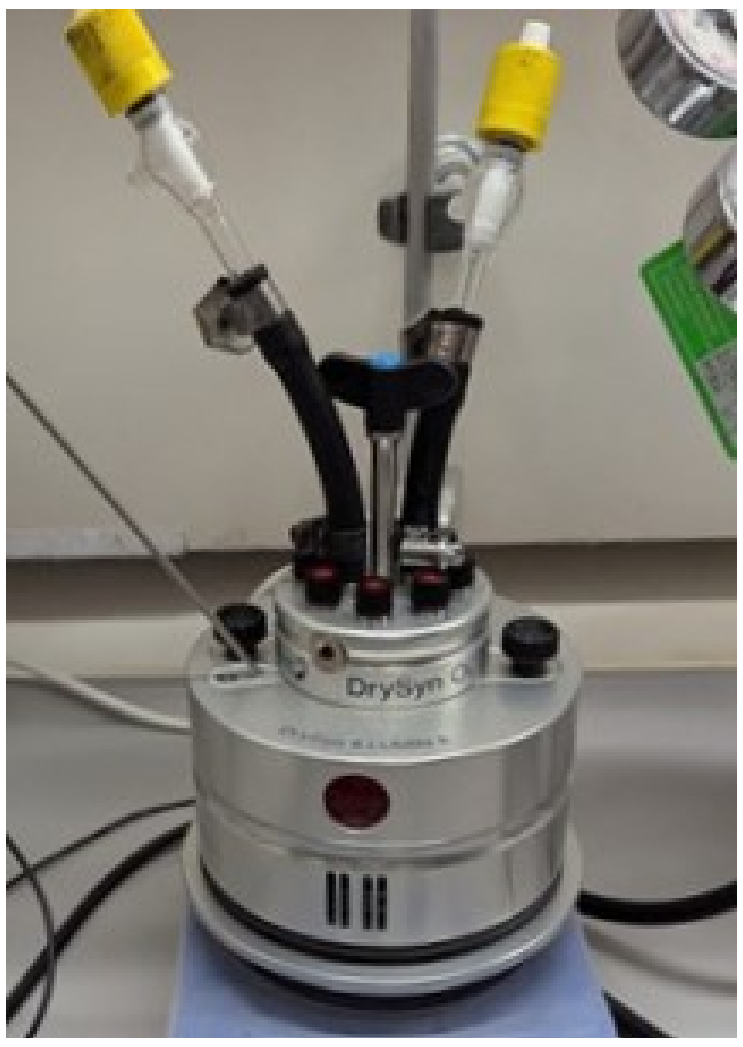

Figure S3. Asynt Illumin8 photoreactor

Table S2. Preliminary tests performed in batch

Entry	5 mol% [$Co_2(CO)_8$]
1	43
2	45

Solvent Screen

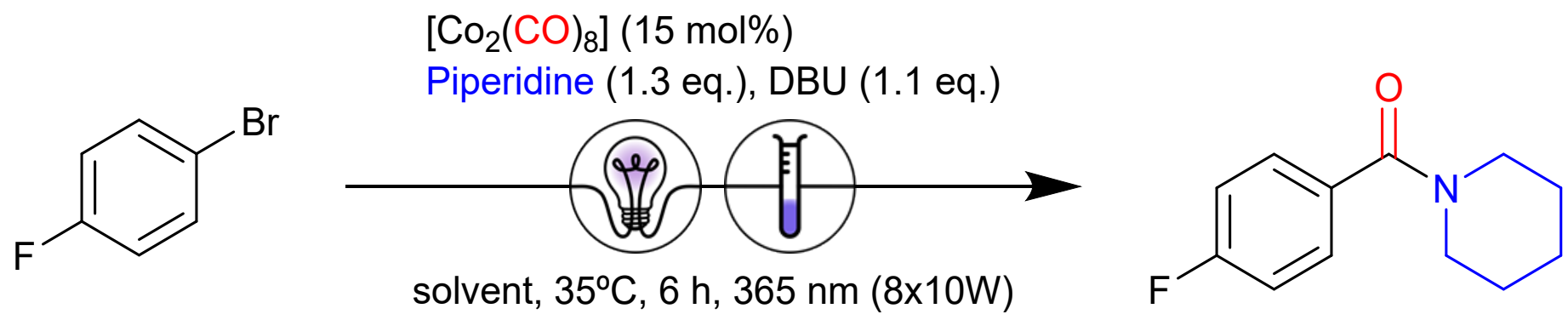

Inside a glovebox, each vial was charged with 13.4 mg of [$Co_2(CO)_8$] (0.038 mmol, 0.15 equiv.), 27 µl of 4-bromofluorobenzene (0.25 mmol, 1 equiv.), 32 µl of piperidine (0.33 mmol, 1.3 equiv.), 41 µl of DBU (0.28 mmol, 1.1 equiv.), and solvent to a total volume of 2 ml. The reactor was sealed in a nitrogen atmosphere and irradiated under stirring for 6 hours at 35 ºC. After that time, an aliquot was taken from each tube and ^{19}F NMR spectroscopy was used to calculate the conversion of the reaction.

Table S3. Solvent screening in batch

Solvent	NMR yield (%)[a]	
	Amide	Fluorobenzene
TAA	74	10
TAA/MeCN[b]	72	28
MeCN	59	28
TAA/DMF[b]	72	17
DMF	72	16
TAA/DMSO[b,c]	36	14
DMSO[c]	29	15
DCM[c]	42	0

TAA = *t*-amyl-OH. [a]NMR yields are calculated from the ratio of the respective integrals from the ^{19}F NMR spectra, taking into account the signals from other secondary product and starting material. [b]Solvent mixture 1:1 v/v. [c]50-55% of starting material unreacted for these three solvents.

Preliminary flow study

Flow conditions were initially investigated using a modified Illumin8 reactor. 2.5 m of FEP tubing (ID: 1 mm) were introduced in the reactor arranged around the carousel, and the reactor mixture was infused using an Apparatus Pump 11 Elite Syringe Pump. The Illumin8 365 nm LED lamps (8x10 W) were used for irradiation. Stock solutions were prepared inside a glovebox (4-bromoanisole: 1 equiv.; piperidine: 1.3 equiv.; [$Co_2(CO)_8$]: 0.125 equiv., DBU: 1.1 equiv.; n-dodecane; solvent: *t*-amyl-alcohol). Yields were calculated by GC as previously described as an average of the three samples.

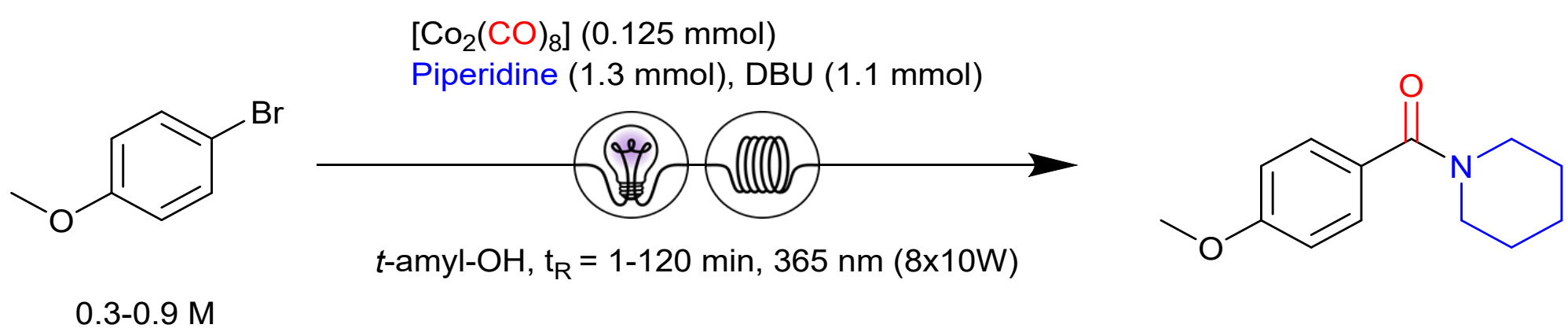

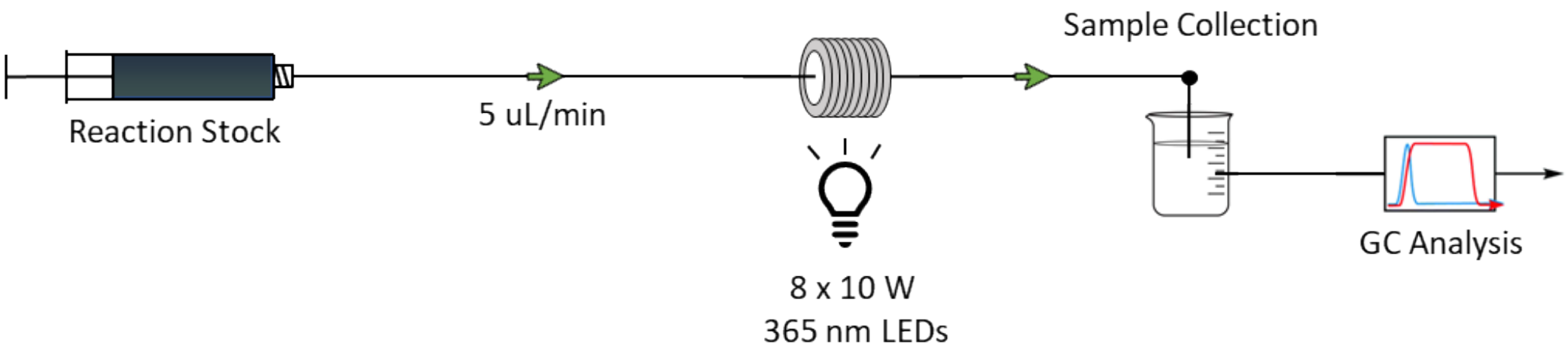

Scheme S1. Reactor set-up in flow

Results were the following:

Table S4. Preliminary flow results

Residence time (min)	Yield (%)[a]	Starting material consumption (%)[a]
**1**	4.4	13.1
**6**	28.2	45.0
**30**	28.7	58.4
**60**	57.8	94.6
**120**	53.9	86.0

[a]GC yields are calculated with an internal standard (n-dodecane). The results are the average of three samples.

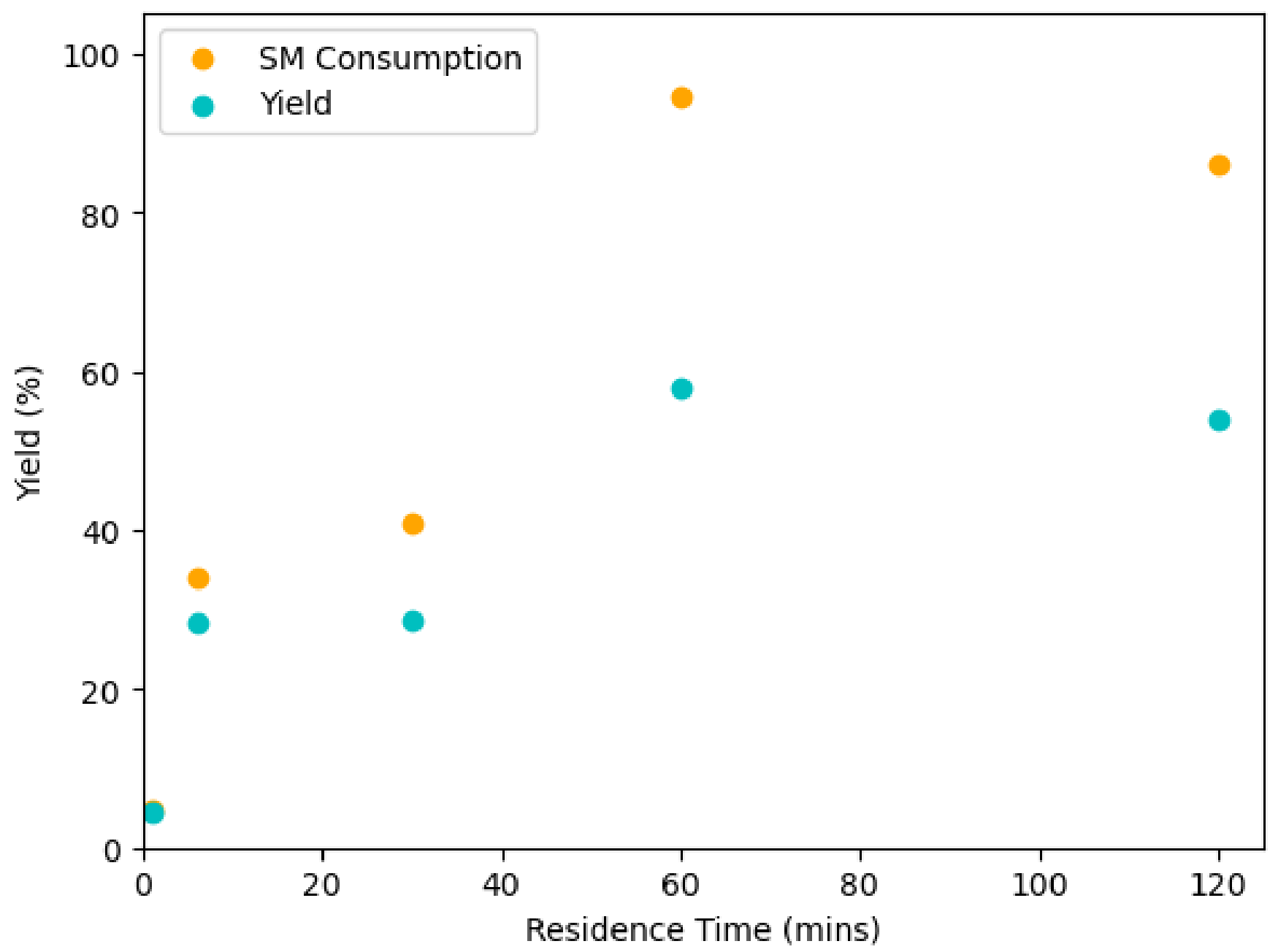

Figure S4. Evolution of the reaction in flow vs the residence time

3. Reaction optimization

Reaction optimizations were carried out using the Vapourtec UV-150 photoreactor (365 nm, 60 W input power), with 4-bromoanisole and piperidine as substrates. A known amount of n-dodecane was added as the internal standard for GC analysis and ratio confirmed at t=0. Stocks solutions where prepared in a glovebox, in the following order of addition: solvent (*t*-amyl-alcohol/THF (1:1 v/v)), [$Co_2(CO)_8$], 4-bromoanisole, DBU, n-dodecane and piperidine. This order of addition minimized reactivity between DBU and the cobalt complex prior to the reaction, which otherwise caused significant gas evolution and formation of a deep blue insoluble oil. The reaction mixture was removed from the glovebox in a Schlenk tube. The flow reactor was flushed with nitrogen and dry THF (15 ml) before infusing the reaction mixture. Steady-state operation was confirmed by taking three 5 ml samples at different reaction times after 1 reactor volume (2 x t_R). Yields were calculated by GC as previously described as an average of the three samples.

3.1. Residence time screening at different catalyst loadings

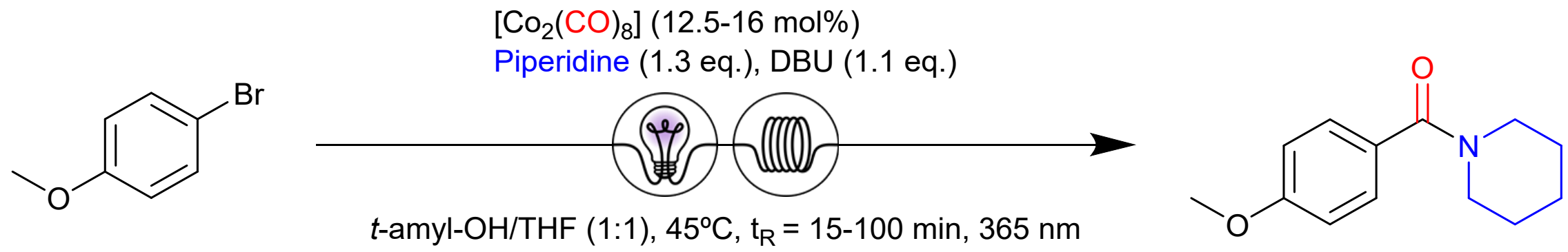

Inside a glovebox, a 0.125 M stock solution of 4-bromoanisole was prepared (4-bromoanisole: 1 equiv.; piperidine: 1.3 equiv.; [$Co_2(CO)_8$]; DBU: 1.1 equiv.; n-dodecane; solvent: *t*-amyl-alcohol/THF (1:1 v/v)). The Vapourtec UV-150 flow reactor was flushed with nitrogen and dry THF (15 ml) before infusing the reaction mixture. Yields were calculated by GC as previously described as an average of the three samples. Two different loadings of [$Co_2(CO)_8$] were tested (12.5 mol% and 16 mol%) at four different residence times.

Table S5. Residence time screening at two different catalyst loadings in flow

	**12.5 mol% [$Co_2(CO)_8$]**	**16 mol% [$Co_2(CO)_8$]**
**Residence time (min)**	**Yield (%)[a]**	**Yield (%)[a]**
**15**	24	-
**30**	48	37
**60**	56	70
**100**	62	73

[a]GC yields are calculated with an internal standard (n-dodecane). The results are the average of three samples.

3.2. Temperature screening

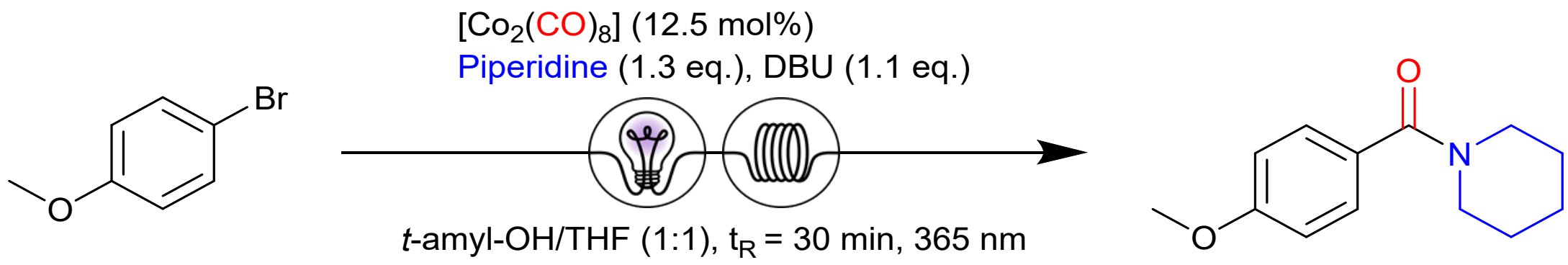

In a glovebox, a 0.125 M stock solution of 4-bromoanisole was prepared (4-bromoanisole: 1 equiv.; piperidine: 1.3 equiv.; $[Co_2(CO)_8]$: 0.125 equiv.; DBU: 1.1 equiv.; n-dodecane; solvent: *t*-amyl-alcohol/THF (1:1 v/v)). The flow reactor was flushed with nitrogen and dry THF (15 ml) before infusing the reaction mixture. The temperature was controlled using the built-in Vapourtec UV-150 reactor, and four different temperatures were selected. The flow rate was set at 0.334 ml/min (t_R = 30 min). Yields were calculated by GC as previously described as an average of the three samples.

Table S6. Temperature screening in flow

Temperature (°C)	Yield (%)[a]
**35**	31
**45**	48
**60**	50
**75**	32

[a]GC yields are calculated with an internal standard (n-dodecane). The results are the average of three samples.

3.3. Concentration screening

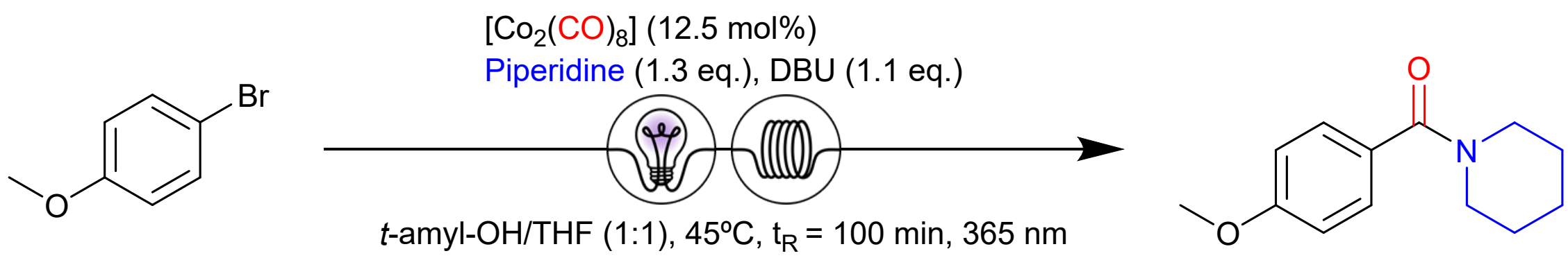

Next, the effect of the concentration in the reaction yield was studied. In a glovebox, different stock solutions with 0.125 M, 0.25 M and 0.5 M of 4-bromoanisole were prepared (4-bromoanisole: 1 equiv.; piperidine: 1.3 equiv.; $[Co_2(CO)_8]$: 0.125 equiv.; DBU: 1.1 equiv.; n-dodecane; solvent: *t*-amyl-alcohol/THF (1:1 v/v)). The Vapourtec UV-150 flow reactor was flushed with nitrogen and dry THF (15 ml) before infusing the reaction mixture. The flow rate was set to 0.100 ml/min (t_R = 100 min) and the temperature at 45 ºC. Yields were calculated by GC as previously described as an average of the three samples.

Table S7. Concentration screening in flow

Concentration (M)	Yield (%)[a]
**0.125**	62

**0.25**	59
**0.5**	12

[a]GC yields are calculated with an internal standard (n-dodecane). The results are the average of three samples.

4. Selectivity optimization

For the selectivity optimization, 4-fluorobromobenzene was used as the substrate, and yields were obtained by quantitative ^{19}F NMR using DMSO-d_6 as solvent. A known amount of trifluorotoluene (δ = -62.01 ppm in DMSO-d_6) was added directly to each aliquot. No work-up or evaporation was performed to the aliquots before analysis. Product peaks were assigned based on literature values and ^{19}F NMR spectra of the pure compounds: 4-fluorobromobenzene (δ = -115.6 ppm in DMSO-d_6),[1] N-(4-Fluorobenzoyl)piperidine (δ = -112.1 ppm in DMSO-d_6),[2] 4-fluorobenzene (δ = -113.6 ppm in DMSO-d_6).[3] An example of an ^{19}F NMR spectrum for an incomplete reaction is shown in Figure S5.

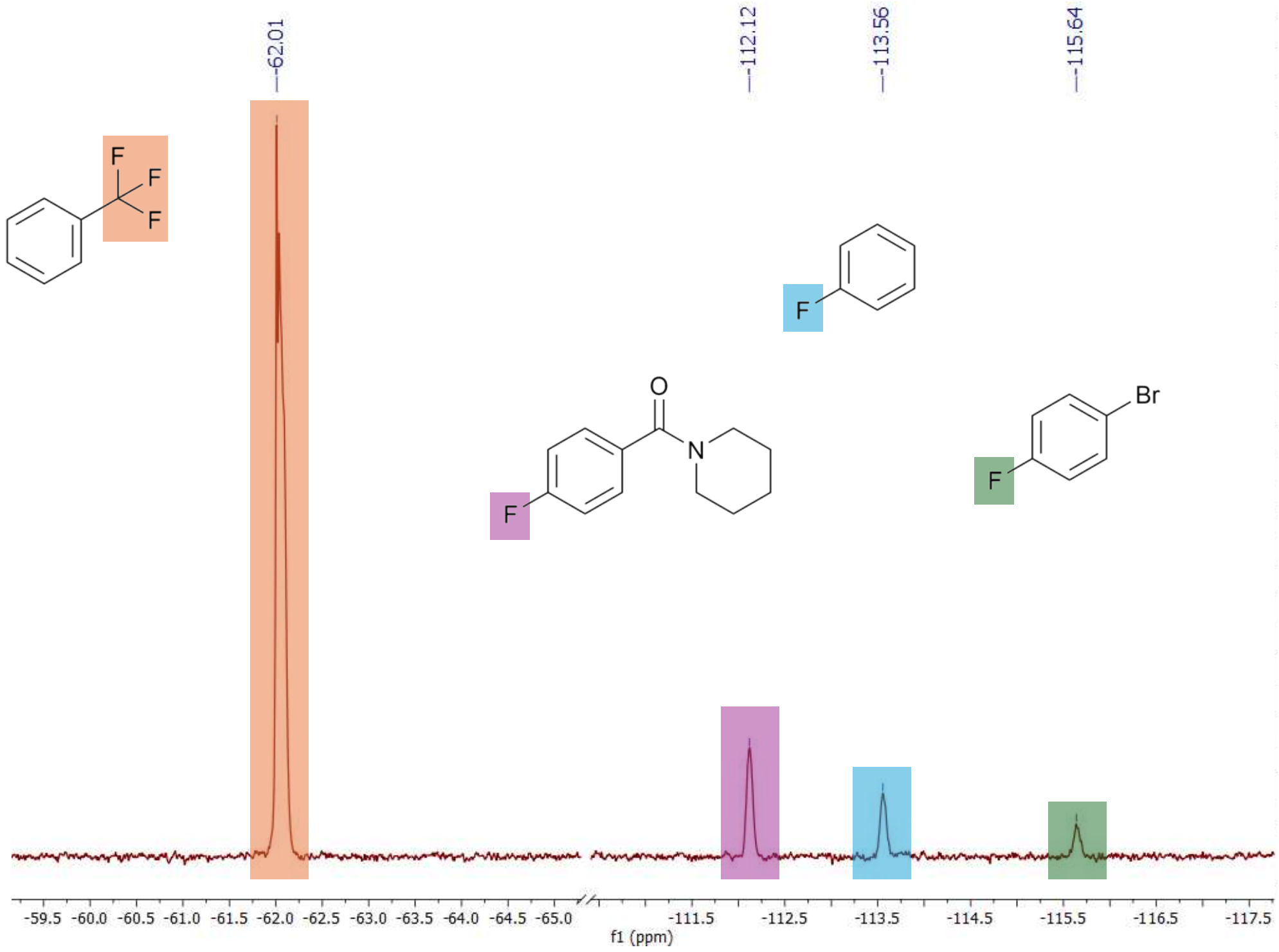

Figure S5. ^{19}F NMR spectrum with the signals assigned to the internal standard, starting material and products

4.1. General procedure

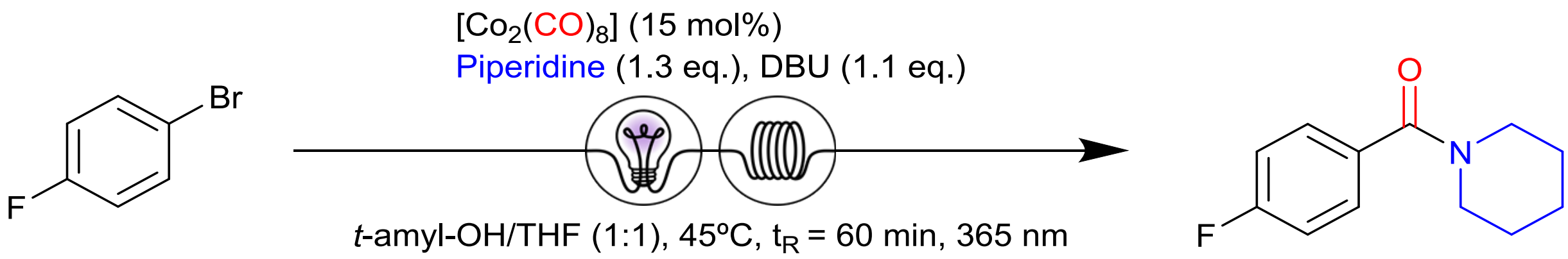

For the selectivity optimization, syringe pumps were used instead of the peristaltic pumps for a better flexibility. Two stock solutions were prepared; the catalyst stock containing 15 mol% of [$Co_2(CO)_8$] in *t*-amyl-OH/THF (1:1 v/v)), and the organic stock (4-fluorobromobenzene: 1 equiv.; piperidine: 1.3 equiv.; DBU: 1.1 equiv., solvent: *t*-

amyl-OH/THF (1:1 v/v)). The two solutions were mixed in a T-junction prior to the reactor inlet (Figure S6), achieving a final concentration of 0.125 M on 4-fluorobromobenzene. For all the experiments in this section, the variables shown in the reaction scheme were used, unless stated otherwise.

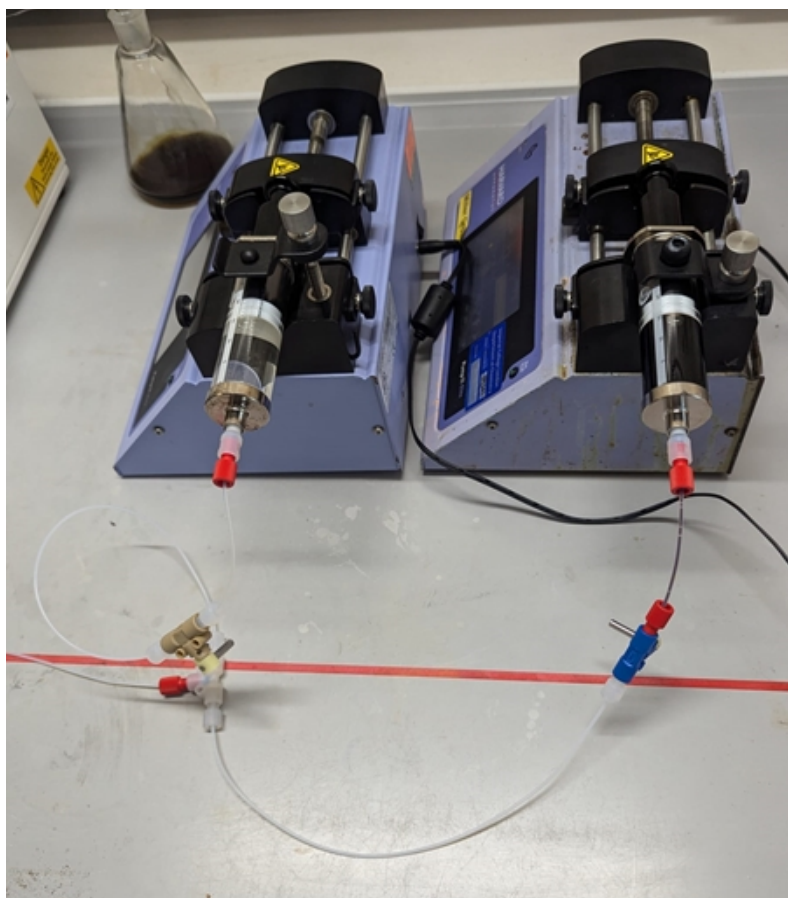

Figure S6. Syringe pumps set-up

Steady-state operation was confirmed by sampling at different reaction times after 0.5 reactor volume (1.5 x t_R). After the collection of the samples, a known amount of reaction mixture was mixed with the internal standard (trifluorotoluene), and yields were calculated by ^{19}F-NMR.

4.2. Catalyst loading

Two stock solutions were prepared; the catalyst stock containing 8.3 mol% or 15 mol% of [$Co_2(CO)_8$] in *t*-amyl-OH/THF (1:1 v/v)), and the organic stock (4-fluorobromobenzene: 1 equiv.; piperidine: 1.3 equiv.; DBU: 1.1 equiv., solvent: t-amyl-OH/THF (1:1 v/v)). The two solutions were mixed in a T-junction prior to the reactor inlet, achieving a final concentration of 0.125 M on 4-fluorobromobenzene. Yields were calculated by ^{19}F-NMR.

Table S8. Catalyst loading influence in selectivity

**[$Co_2(CO)_8$] loading**	**NMR yields (%)[a]**	
	Amide	Fluorobenzene
8.3 mol%	42	22
15 mol%	68	20

[a]Q-NMR yields are calculated with an internal standard (trifluorotoluene).

4.3. Piperidine and DBU loading

Two stock solutions were prepared; the catalyst stock containing 15 mol% of [$Co_2(CO)_8$] in *t*-amyl-OH/THF (1:1 v/v)), and the organic stock (4-fluorobromobenzene: 1 equiv.; piperidine; DBU; solvent: t-amyl-OH/THF (1:1 v/v)). The two solutions were mixed in a T-junction prior to the reactor inlet, achieving a final concentration of 0.125 M on 4-fluorobromobenzene. Yields were calculated by ^{19}F-NMR.

Table S9. Piperidine and DBU loading influence in selectivity

Reagent	Equiv.	NMR yields (%)[a]	
		Amide	Fluorobenzene
**Piperidine**	**1**	68	22
	**1.3**	70	22
	**1.5**	74	17
	**2**	72	20
**DBU**	**1.1**	70	22
	**1.5**	72	20

[a]Q-NMR yields are calculated with an internal standard (trifluorotoluene).

4.4. Solvent screening in flow

Two stock solutions were prepared; the catalyst stock containing 15 mol% of $[Co_2(CO)_8]$ in the appropriated solvent (Table S10), and the organic stock (4-fluorobromobenzene: 1 equiv.; piperidine: 1.3 equiv.; DBU: 1.1 equiv.; solvent). The two solutions were mixed in a T-junction prior to the reactor inlet, achieving a final concentration of 0.125 M on 4-fluorobromobenzene. Yields were calculated by ^{19}F-NMR. Precipitation was observed with neat *t*-amyl-OH (TAA) prior of the reaction, and neat THF caused significant amounts of precipitation inside the reactor coil, increasing the risks of blockage.

Table S10. Solvent influence in selectivity in flow

Solvent	NMR yield (%)[a]	
	**Amide**	**Fluorobenzene**
**TAA/THF (1/1)**	70	22
**TAA[b]**	62	13
**THF**	72	17
**THF/MeCN (1/1)[c]**	46	30
**DMF**	57	22

[a]Q-NMR yields are calculated with an internal standard (trifluorotoluene). [b]16% of 4-fluorobromobenzene unreacted. [c]11% of 4-fluorobromobenzene unreacted.

4.5. Variation of wavelength of light

Two stock solutions were prepared; the catalyst stock containing 15 mol% of $[Co_2(CO)_8]$ in *t*-amyl-OH/THF (1:1 v/v)), and the organic stock (4-fluorobromobenzene: 1 equiv.; piperidine: 1.3 equiv.; DBU: 1.1 equiv., solvent: t-amyl-OH/THF (1:1 v/v)). The two solutions were mixed in a T-junction prior to the reactor inlet, achieving a final concentration of 0.125 M on 4-fluorobromobenzene. The light source of the Vapourtec photoreactor was change to a 405 nm 60 W LED. Different residence times were tested (Table S11), and higher catalyst loading and concentration (Table S12). Yields were calculated by ^{19}F-NMR.

Table S11. Selectivity at different residence times using a 405 nm irradiation source

Residence time (min)	NMR yield (%)[a]

	Amide	Fluorobenzene	Starting material
**60**	53	10	22
**120**	68	11	3
**180**	72	8	9

[a]Q-NMR yields are calculated with an internal standard (trifluorotoluene).

Table S12. Selectivity using 405 nm irradiation source and different conditions

Conditions	Residence time (min)	NMR yield (%)[a]		
		Amide	Fluorobenzene	Starting material
**20 mol% [$Co_2(CO)_8$] loading**	120	70	10	6
**0.25 M in 4-fluorobromobenzene**	180	74	12	1

[a]Q-NMR yields are calculated with an internal standard (trifluorotoluene).

4.6. Control reactions

Different control reactions were performed following the general procedure, in absence of catalyst, light, DBU or piperidine. Two stock solutions were prepared; the catalyst stock containing 15 mol% of [$Co_2(CO)_8$] in *t*-amyl-OH/THF (1:1 v/v)) (or only solvent mixture for the no-catalyst control reaction), and the organic stock (4-fluorobromobenzene: 1 equiv.; piperidine; DBU; solvent: *t*-amyl-OH/THF (1:1 v/v)). The two solutions were mixed in a T-junction prior to the reactor inlet, achieving a final concentration of 0.125 M on 4-fluorobromobenzene. Yields were calculated by ^{19}F-NMR.

Table S13. Control reactions

Conditions	NMR Yield (%)[a]		
	Amide	Fluorobenzene	Starting material
**No catalyst**	0	0	100
**No irradiation**	0	0	100
**No base**	9	0	91
**No piperidine**	0	13	59

[a]Q-NMR yields are calculated with an internal standard (trifluorotoluene).

5. ²H NMR spectra

The synthesis of *d*-piperidine was performed following a procedure found in literature.[4]

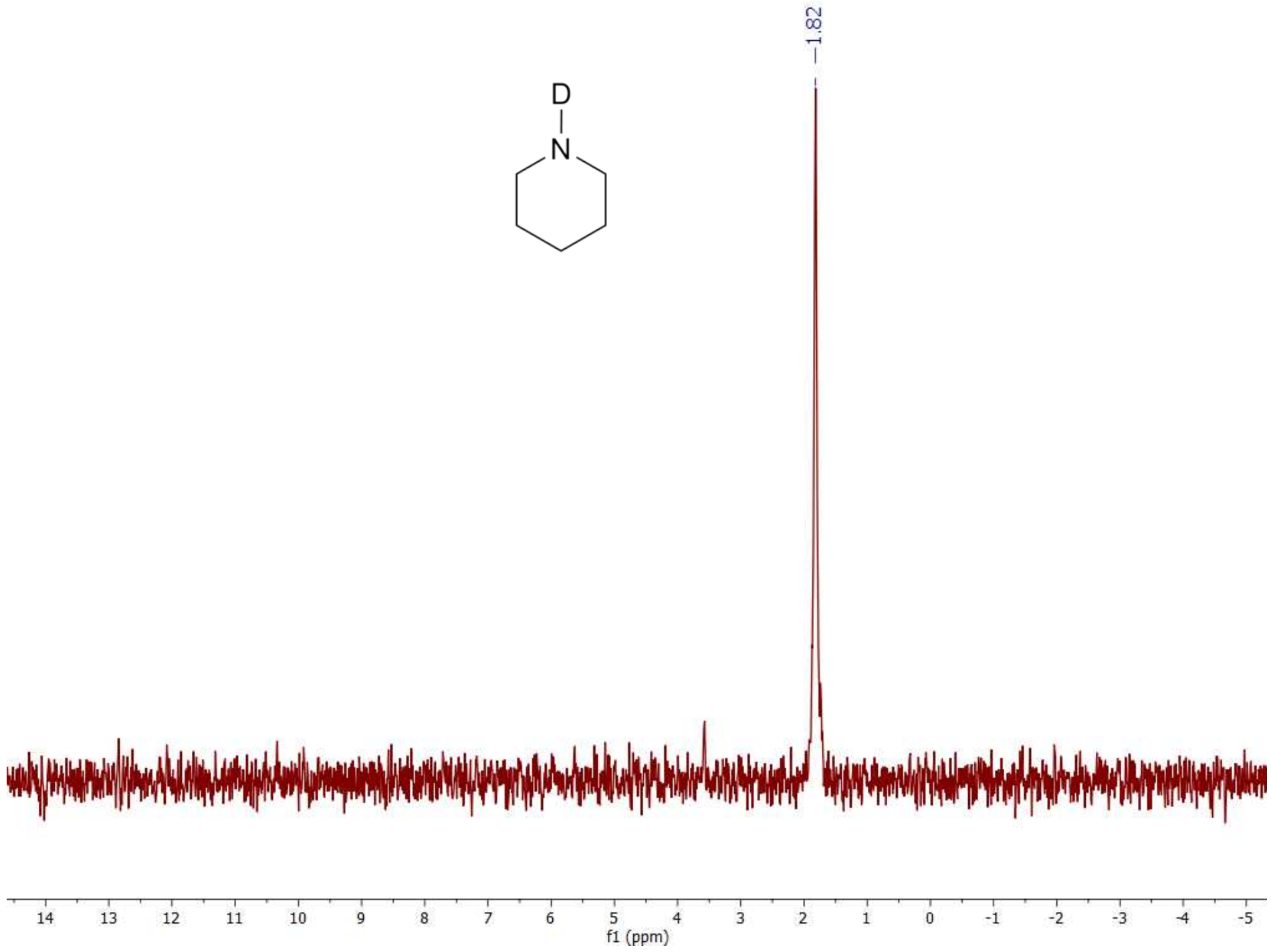

Figure S7. ²H NMR spectrum of d-piperidine, THF, 500 MHz

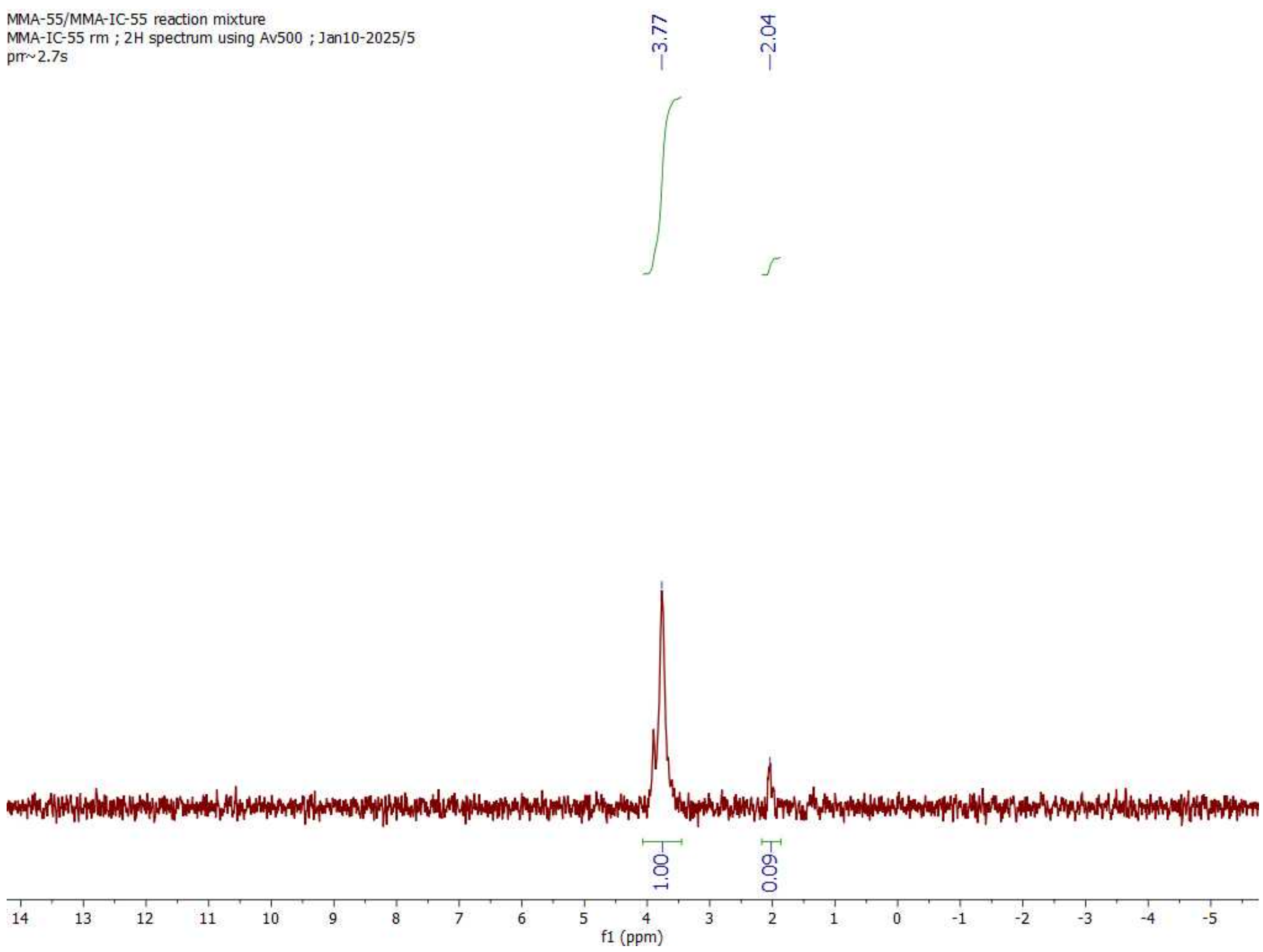

Figure S8. ²H NMR spectrum of the reaction mixture, after the carbonylation reaction of 4-bromofluorobenzene with d-piperidine, following the general procedure described in section 3., THF, 500 MHz

6. Reaction scope

The reaction scope was performed following a general procedure, using two syringes containing the catalyst stock and the organic stock.

6.1. General procedure for aminocarbonylation reactions

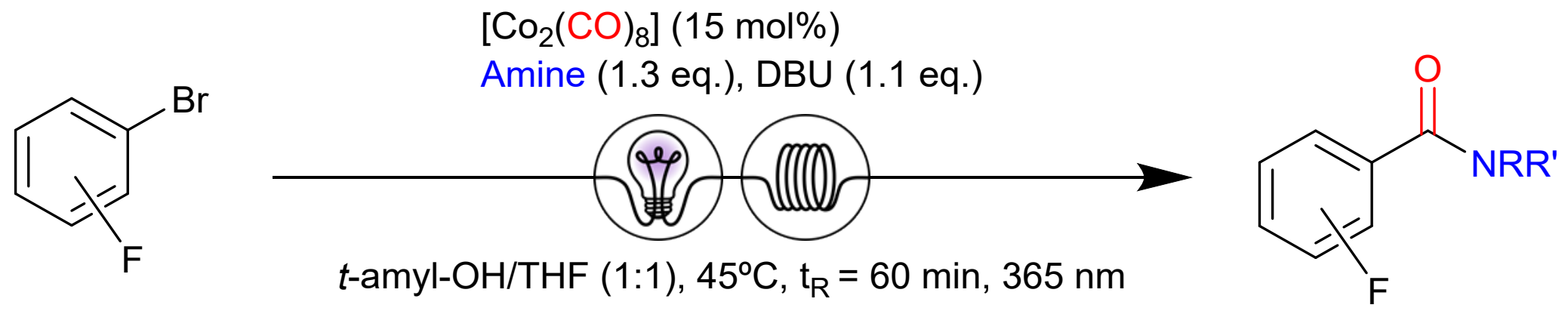

In a typical procedure, a stock solution of $[Co_2(CO)_8]$ in a 1:1 v/v mixture of *t*-amyl-OH/THF and a separate organic stock solution containing 2/4-fluorobromobenzene (1 eq, 0.25 M), the corresponding amine (1.3 equiv.), DBU (1.1 equiv.), and 1:1 v/v mixture of *t*-amyl-OH/THF were prepared and loaded into 50 mL syringes. The solutions were infused at a total flow rate of 0.167 ml/min (t_R = 60 minutes) and mixed prior to entering the photoreactor using a T-mixer (part number: P-714, supplier: VWR), this resulted in a 0.125 M concentration in 4-fluorobromobenzene, and a catalyst loading of 15 mol%. Three 5 ml samples were collected sequentially after 1.5 x t_R, and yields determined using quantitative ^{19}F NMR or ^{1}H NMR spectroscopy, using trifluorotoluene or trimethoxybenzene respectively as internal standard. For ^{19}F NMR, the samples were prepared directly with the reaction mixture, adding the trifluorotoluene dissolved in the deuterated solvent (DMSO-d_6). The preparation of the samples for ^{1}H NMR included a previous wash with 1M HCl aqueous solution, and extraction with ethyl acetate. Trimethoxybenzene was added after the sample was submitted to high vacuum for at least 10 minutes. The signals of the products were assigned based on the values found in the literature.

(4-fluorophenyl)(piperidino)methanone (1): ^{19}F NMR (377 MHz, DMSO-d_6, δ): -112.00 (s, 1F).[2]

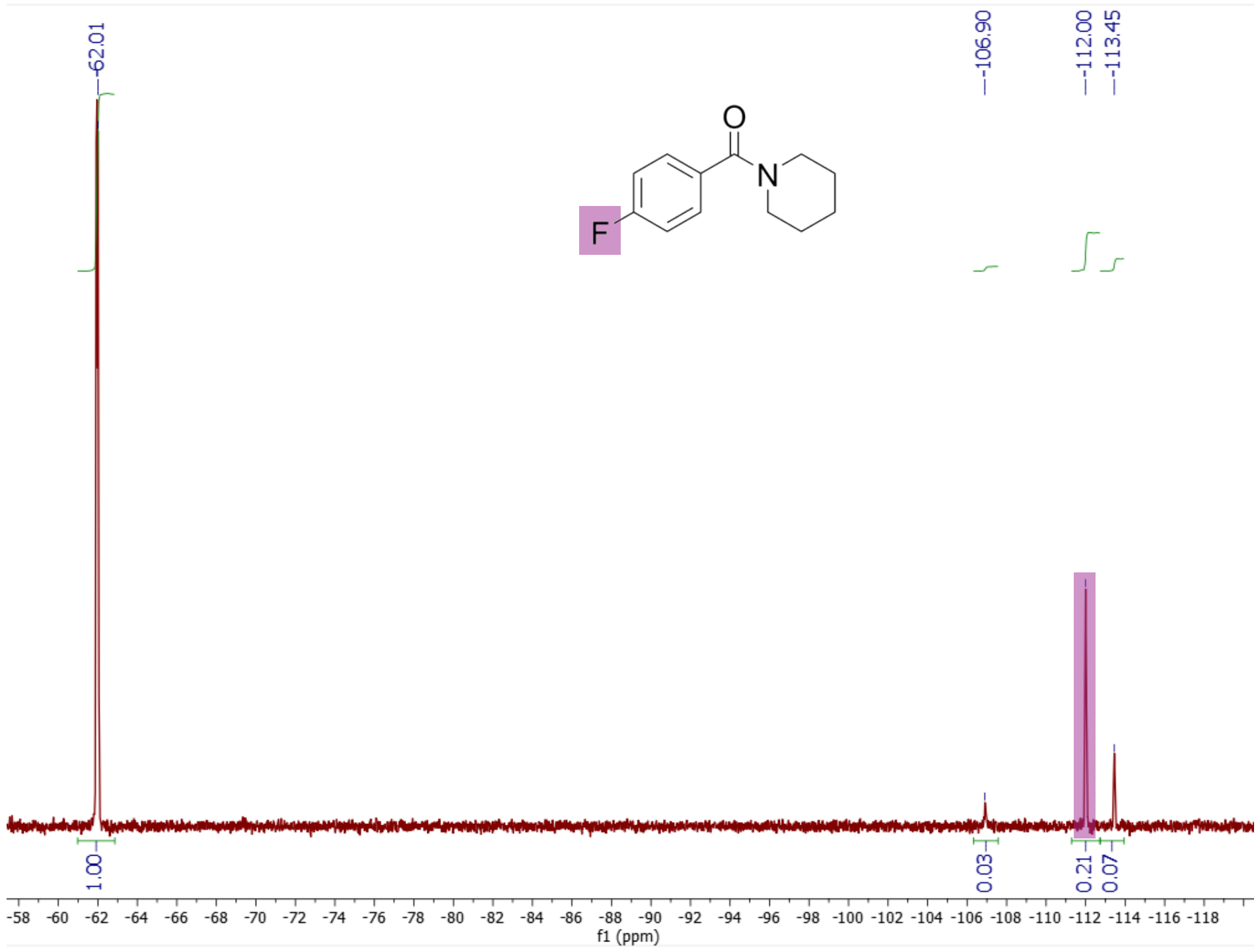

*Figure S9. ^{19}F NMR spectrum of the reaction mixture of the synthesis of **1**. Internal standard: trifluorotoluene (δ = -62.01 ppm in DMSO-d_6)*

(4-fluorophenyl)(pyrrolidin-1-yl)methanone (2): ^{19}F NMR (377 MHz, DMSO-d_6, δ): -110.75 (s, 1F).[5]

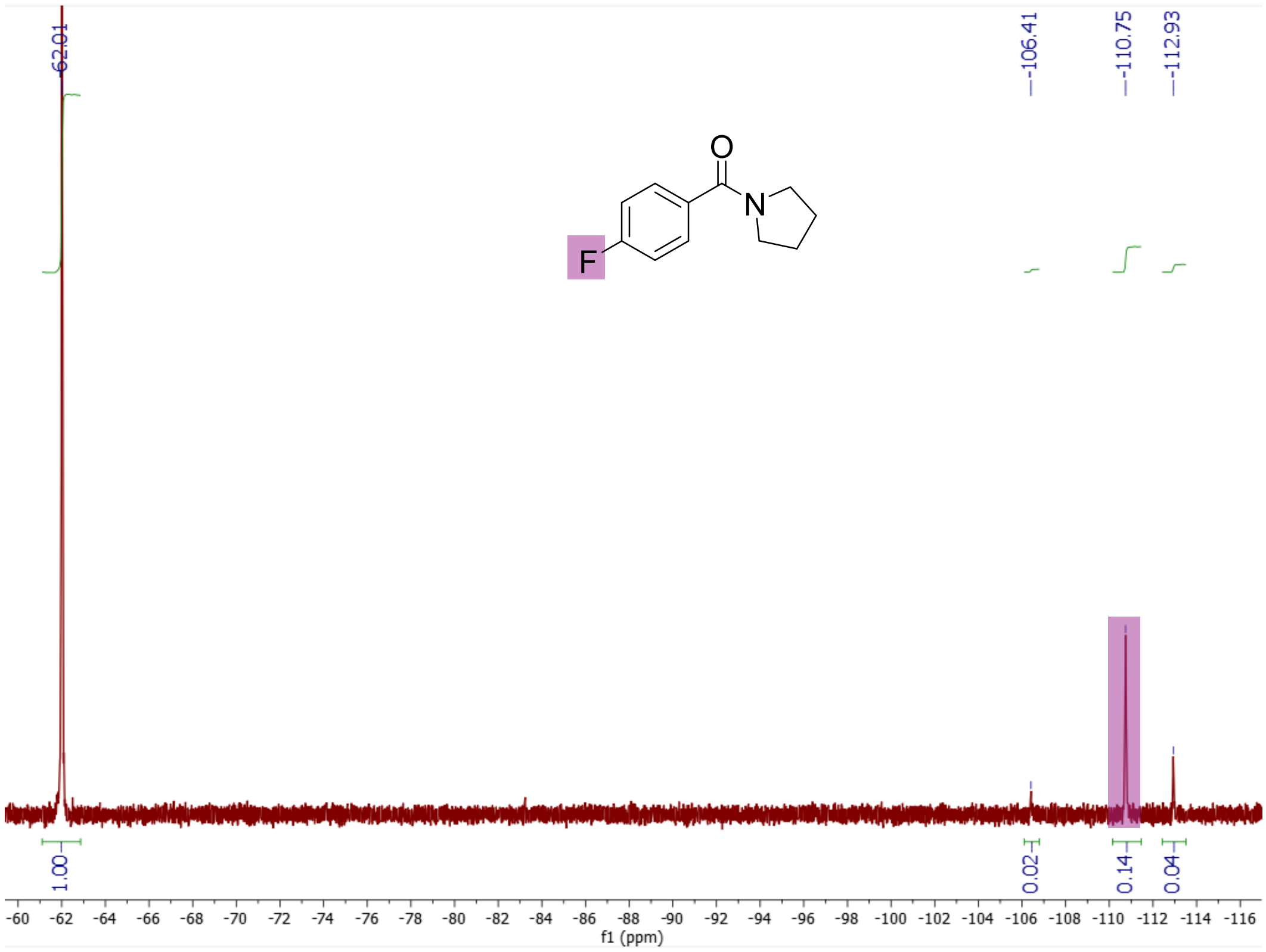

*Figure S10. ^{19}F NMR spectrum of the reaction mixture of the synthesis of **2**. Internal standard: trifluorotoluene (δ = -62.01 ppm in DMSO-d_6)*

N-butyl-4-fluorobenzamide (3): ^{19}F NMR (377 MHz, DMSO-d_6, δ): -110.25 (s, 1F).[6]

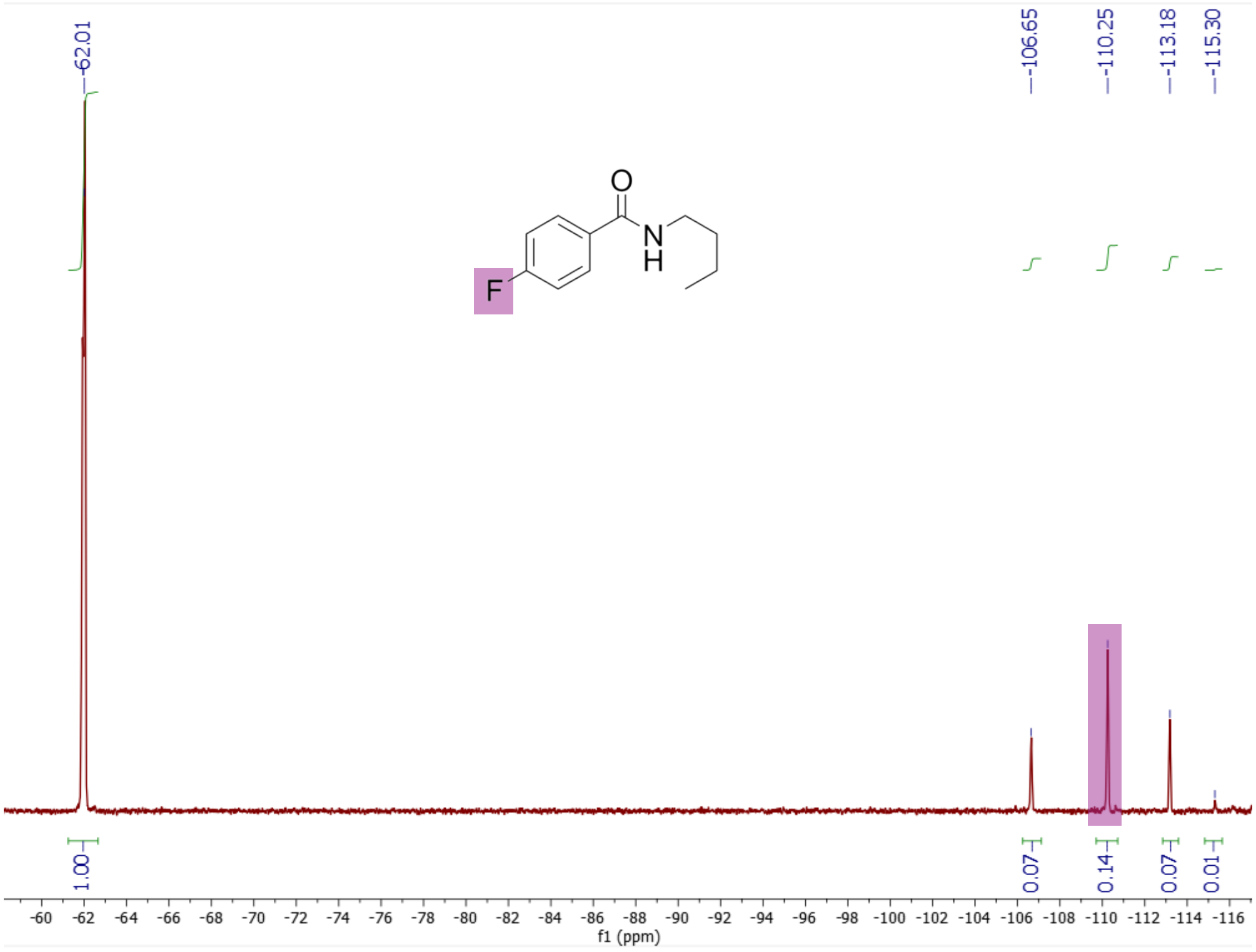

*Figure S11. ^{19}F NMR spectrum of the reaction mixture of the synthesis of **3**. Internal standard: trifluorotoluene (δ = -62.01 ppm in DMSO-d_6)*

N,N-diethyl-4-fluorobenzamide (4): ^{19}F NMR (377 MHz, DMSO-d_6, δ): -112.45 (s, 1F).[7]

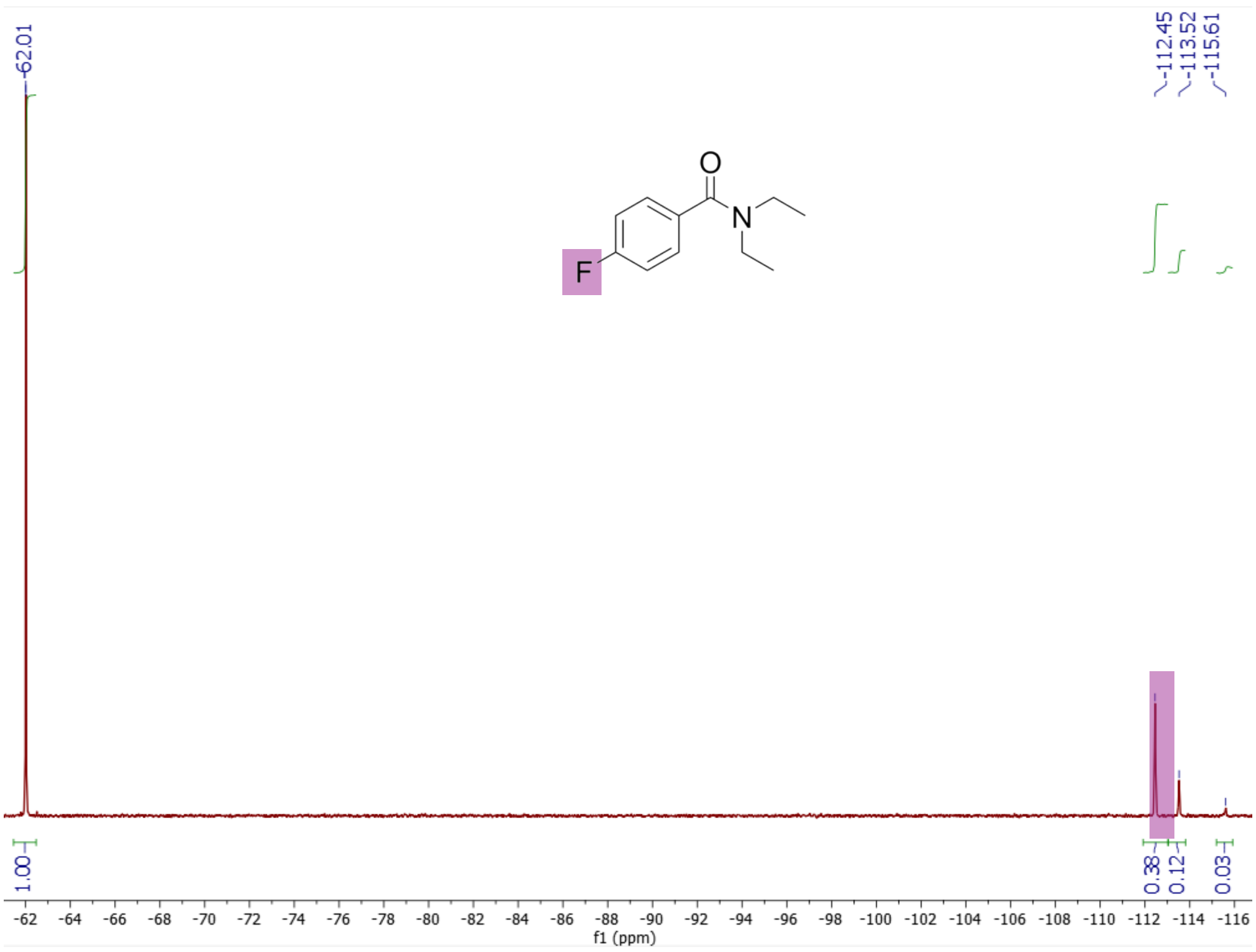

*Figure S12. ^{19}F NMR spectrum of the reaction mixture of the synthesis of **4**. Internal standard: trifluorotoluene (δ = -62.01 ppm in DMSO-d_6)*

N-cyclohexyl-4-fluorobenzamide (5): ^{19}F NMR (377 MHz, DMSO-d_6, δ): -110.46 (s, 1F).[8]

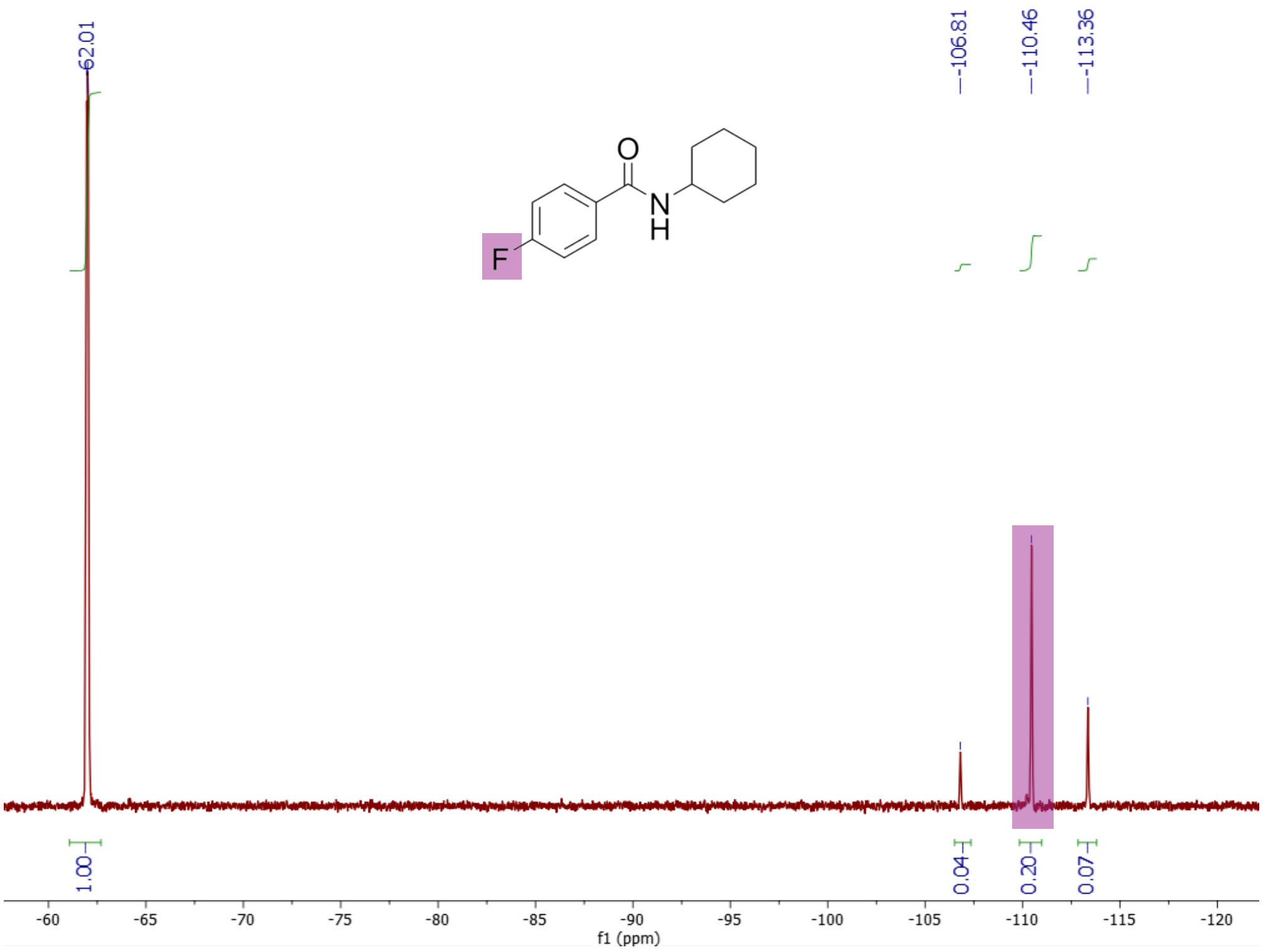

*Figure S13. ^{19}F NMR spectrum of the reaction mixture of the synthesis of **5**. Internal standard: trifluorotoluene (δ = -62.01 ppm in DMSO-d$_6$)*

N-(tert-Butyl)-4-fluorobenzamide (6): ^{19}F NMR (377 MHz, DMSO-d$_6$, δ): -110.92 (s, 1F).[9]

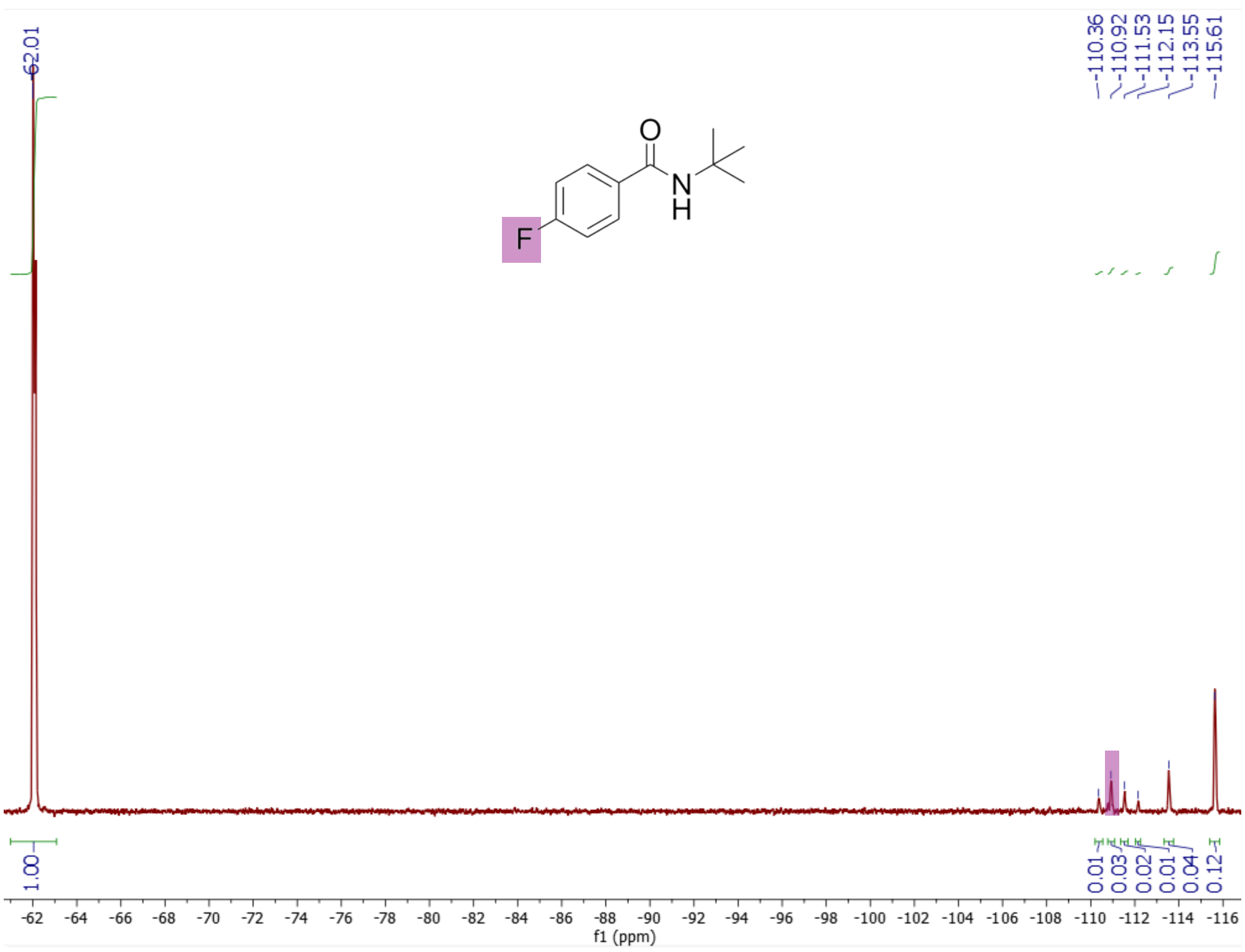

*Figure S14. ^{19}F NMR spectrum of the reaction mixture of the synthesis of **6**. Internal standard: trifluorotoluene (δ = -62.01 ppm in DMSO-d$_6$)*

(2-Fluorophenyl)(1-piperidinyl)methanone (7): ^{19}F NMR (377 MHz, DMSO-d$_6$, δ): -117.07 (s, 1F).[10]

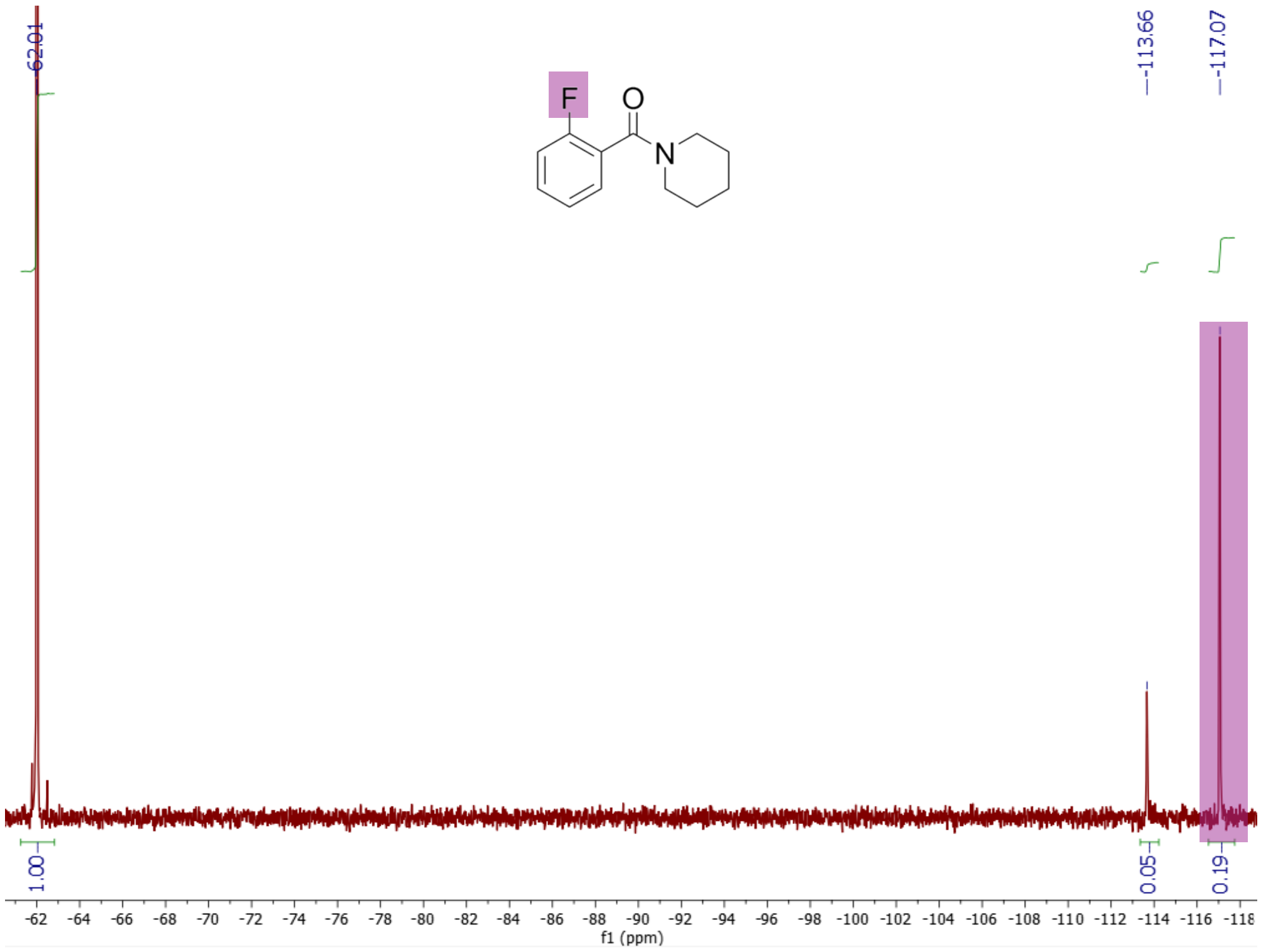

*Figure S15. ^{19}F NMR spectrum of the reaction mixture of the synthesis of **7**. Internal standard: trifluorotoluene (δ = -62.01 ppm in DMSO-d$_6$)*

(2-Fluorophenyl)(1-piperidinyl)methanone (8): ^{19}F NMR (377 MHz, DMSO-d$_6$, δ): -115.60 (s, 1F).[11]

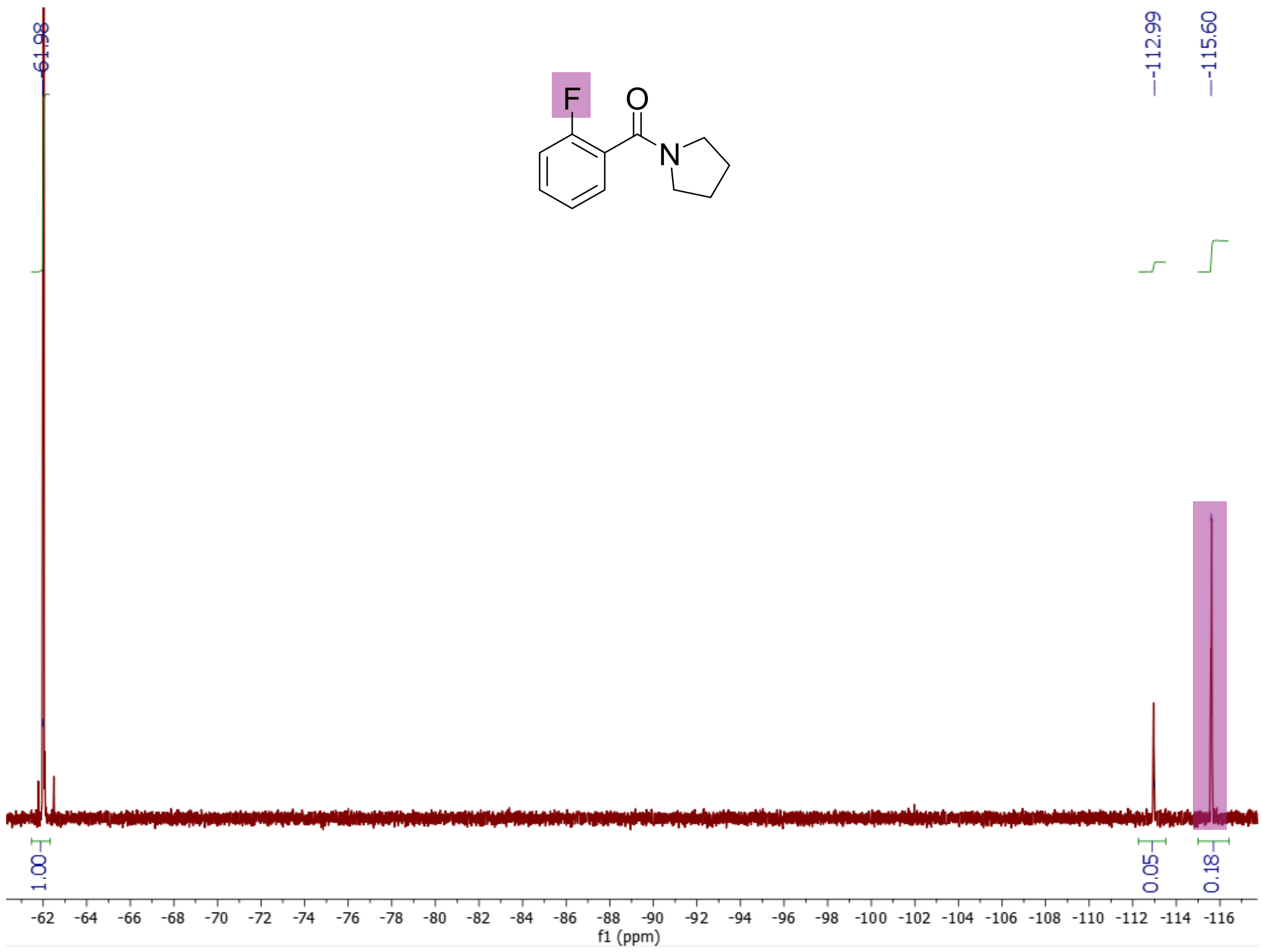

*Figure S16. ^{19}F NMR spectrum of the reaction mixture of the synthesis of **8**. Internal standard: trifluorotoluene (δ = -62.01 ppm in DMSO-d$_6$)*

N-butyl-2-fluorobenzamide (9): ^{19}F NMR (377 MHz, DMSO-d$_6$, δ): -114.24 (s, 1F).[10]

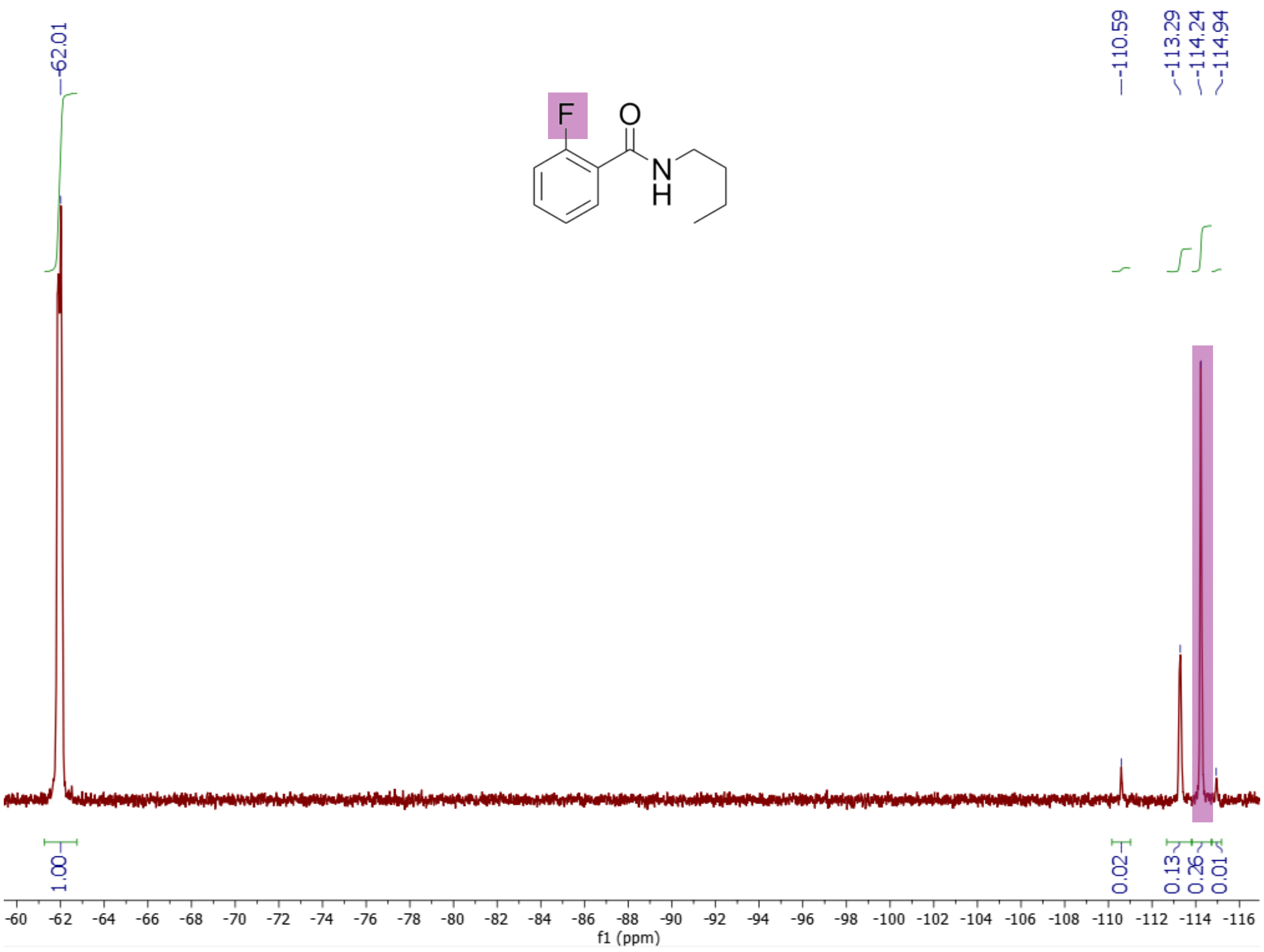

*Figure S17. ^{19}F NMR spectrum of the reaction mixture of the synthesis of **9**. Internal standard: trifluorotoluene (δ = -62.01 ppm in DMSO-d_6)*

N,N-diethyl-2-fluorobenzamide (10): ^{19}F NMR (377 MHz, DMSO-d_6, δ): -117.12 (s, 1F).[10]

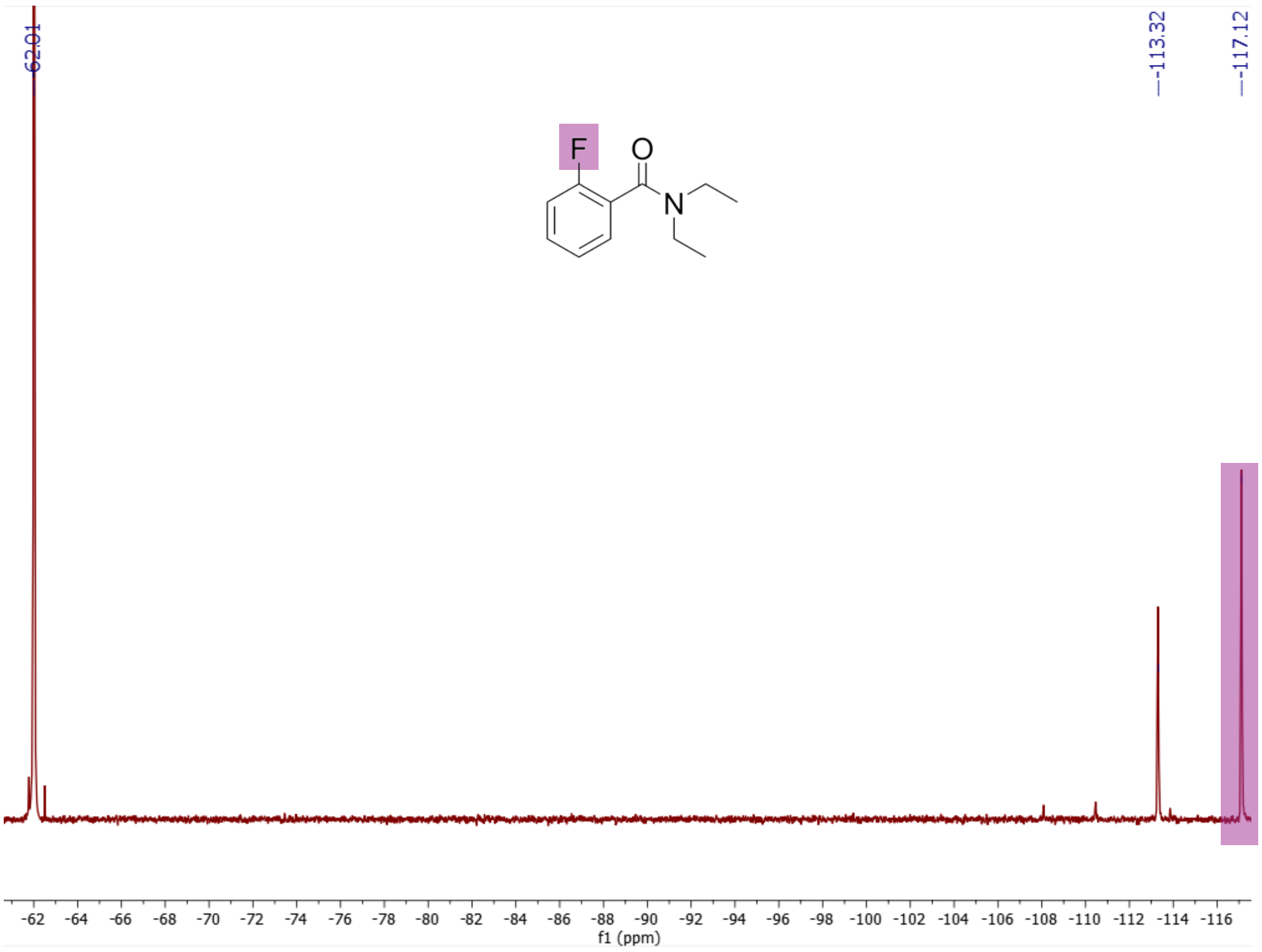

*Figure S18. ^{19}F NMR spectrum of the reaction mixture of the synthesis of **10**. Internal standard: trifluorotoluene (δ = -62.01 ppm in DMSO-d_6)*

N-cyclohexyl-2-fluorobenzamide (11): ^{19}F NMR (377 MHz, DMSO-d_6, δ): -114.85 (s, 1F).[10]

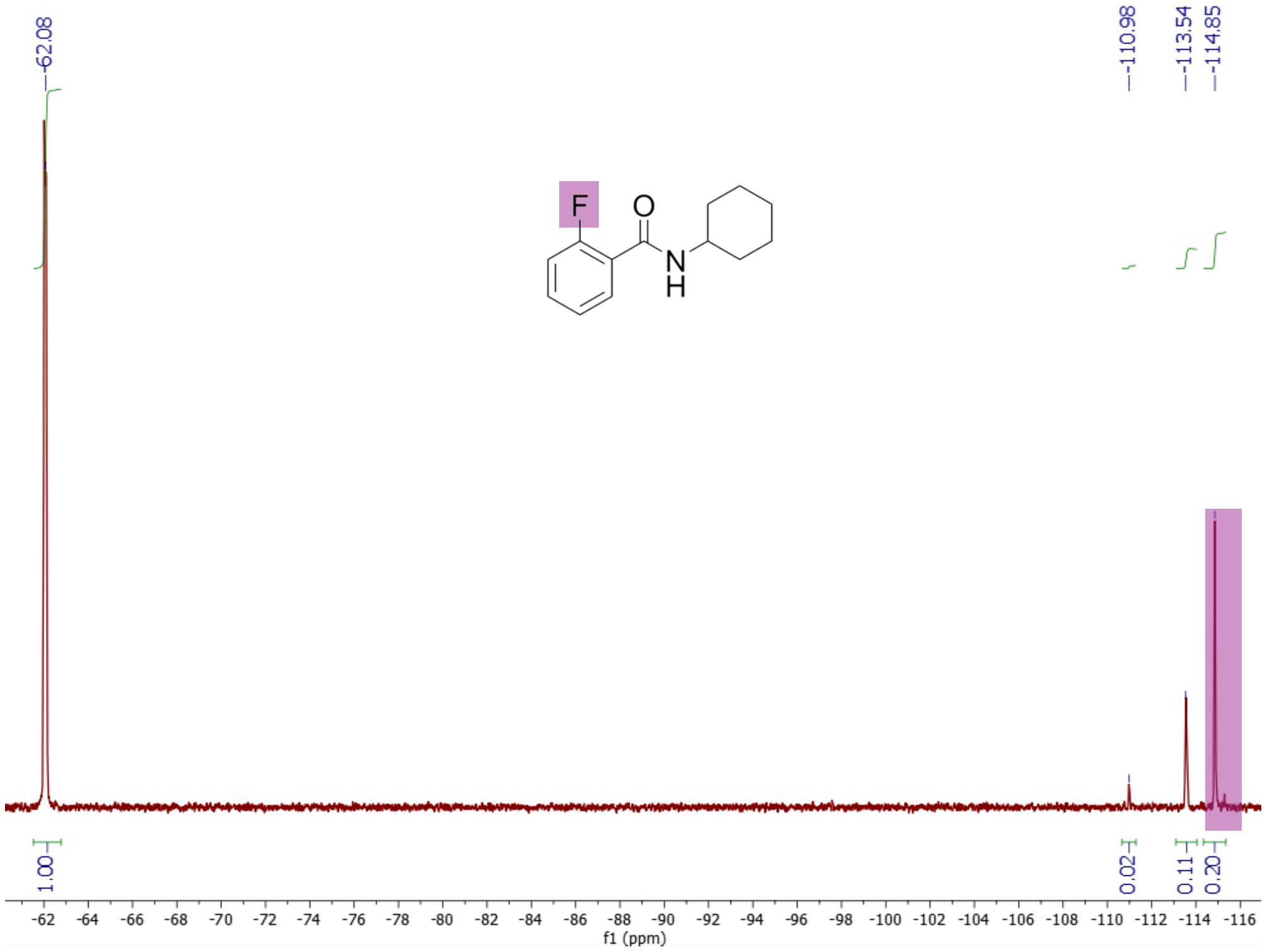

*Figure S19. ^{19}F NMR spectrum of the reaction mixture of the synthesis of **11**. Internal standard: trifluorotoluene (δ = -62.01 ppm in DMSO-d_6)*

N-(tert-butyl)-2-fluorobenzamide (12): ^{19}F NMR (377 MHz, DMSO-d_6, δ): -114.50 (s, 1F).[12]

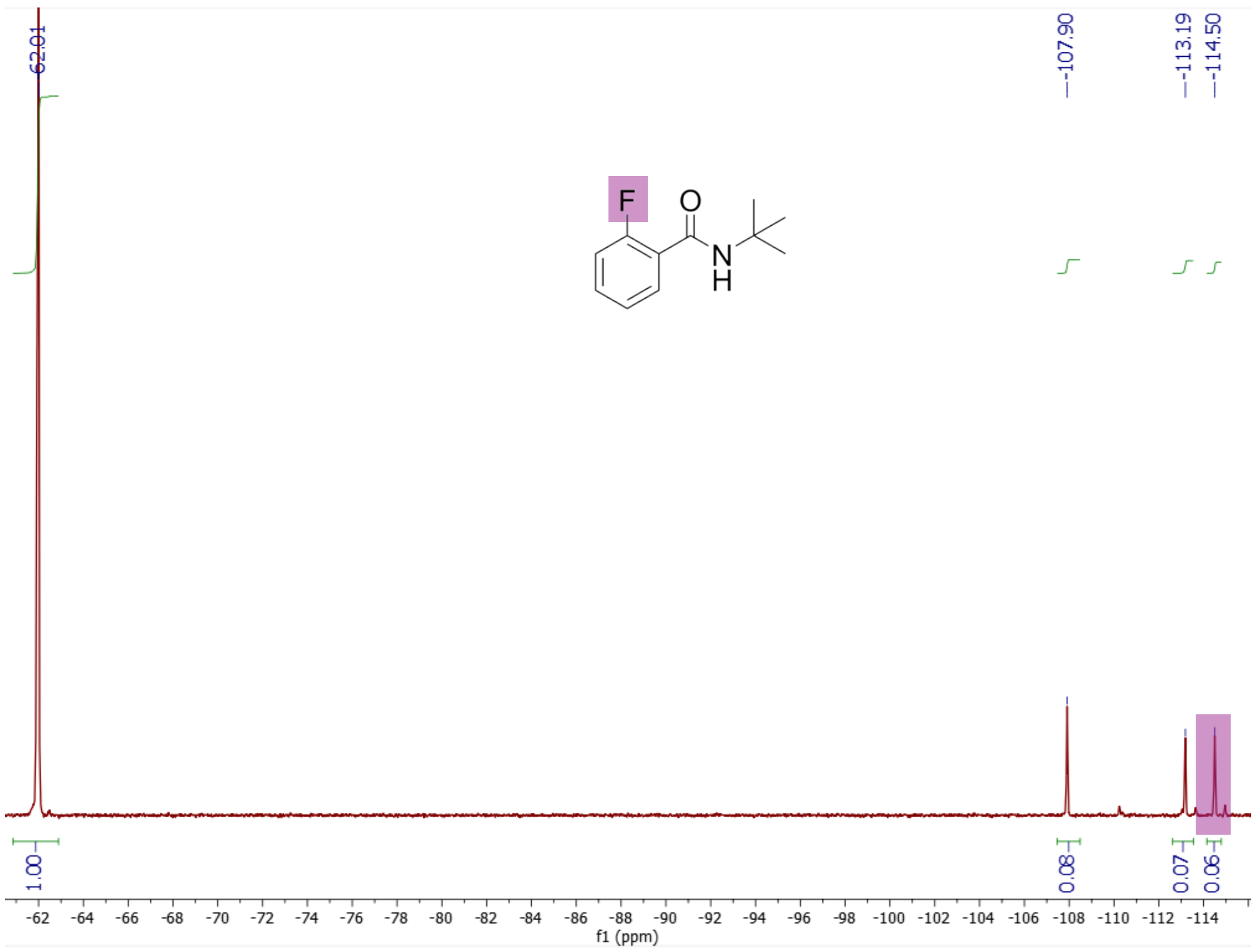

*Figure S20. ^{19}F NMR spectrum of the reaction mixture of the synthesis of **12**. Internal standard: trifluorotoluene (δ = -62.01 ppm in DMSO-d_6)*

Pyrrolidin-1-yl(4-(trifluoromethyl)phenyl)methanone (13): ^{1}H NMR (400 MHz, $CDCl_3$, δ): 7.68 – 7.59 (m, 4H), 3.66 (t, *J* = 6.9 Hz, 2H), 3.39 (t, *J* = 6.6 Hz, 2H), 2.00 – 1.84 (m, 4H).[13]

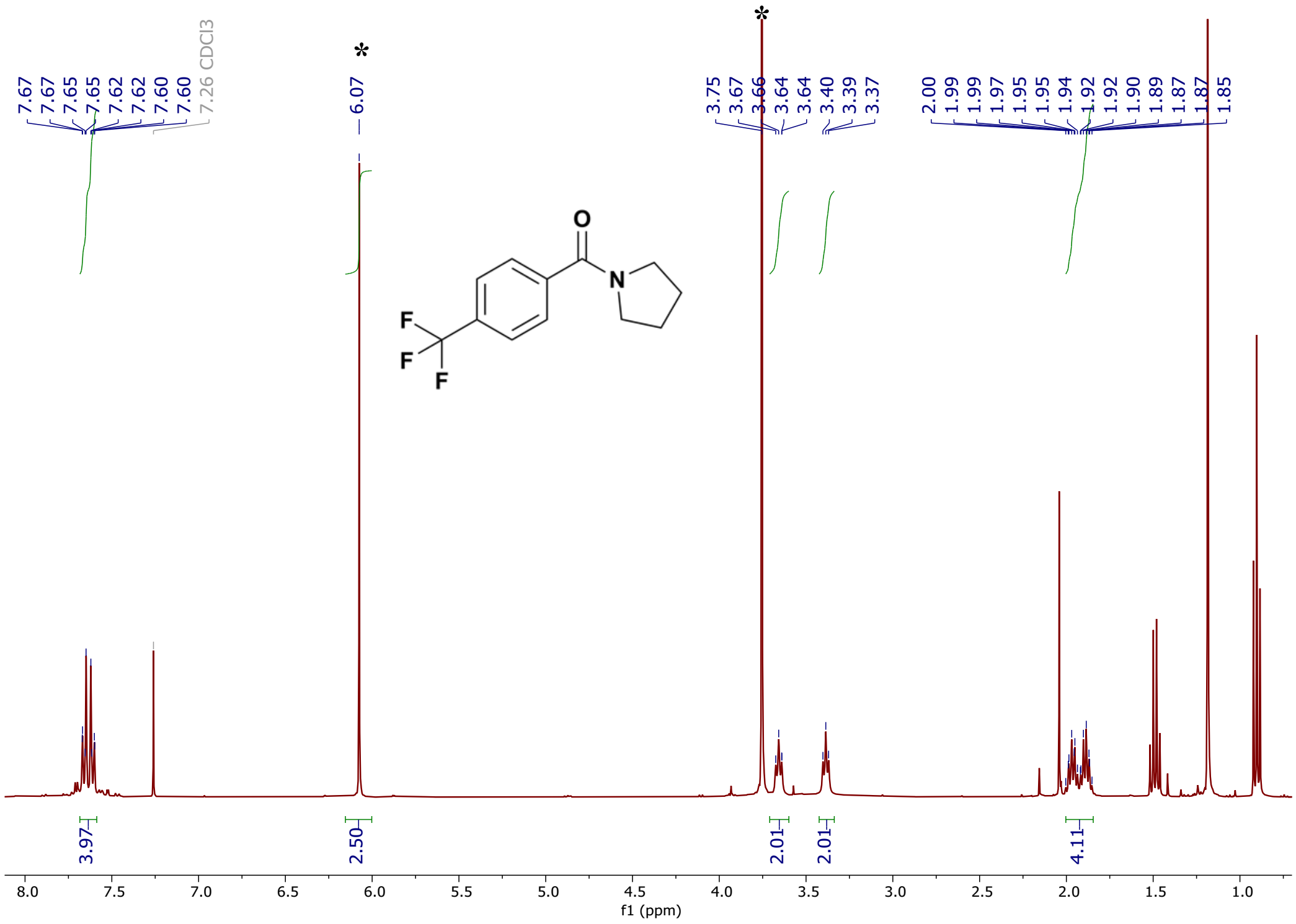

*Figure S21. ^{1}H NMR spectrum of the reaction mixture after acid wash of the synthesis of **13**. *Internal standard: 1,3,5-trimethoxybenzene.*

Pyrrolidin-1-yl(3-(trifluoromethyl)phenyl)methanone (14): ^{1}H NMR (400 MHz, $CDCl_3$, δ): 7.79 (s, 1H), 7.73 - 7.63 (m, 2H), 7.53 (t, *J* = 7.7 Hz, 1H), 3.66 (t, *J* = 7.0 Hz, 2H), 3.41 (t, *J* = 6.6 Hz, 2H), 2.02 – 1.85 (m, 4H).[13]

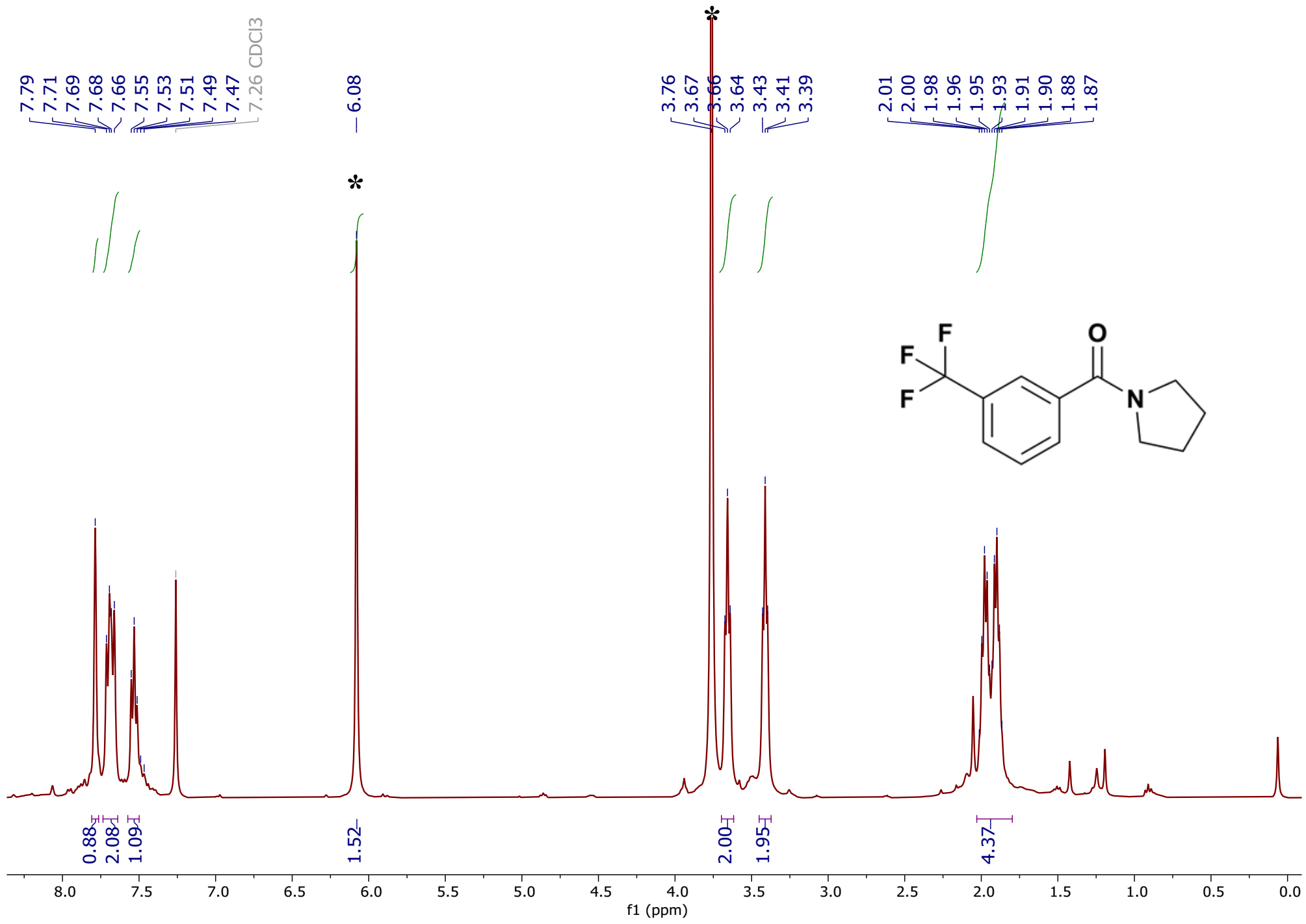

*Figure S22 ^{1}H NMR spectrum of the reaction mixture after acid wash of the synthesis of **14**. *Internal standard: 1,3,5-trimethoxybenzene.*

Pyrrolidin-1-yl(2-(trifluoromethyl)phenyl)methanone (15): ^{1}H NMR (400 MHz, $CDCl_3$, δ): 7.68 (d, J = 8.0 Hz, 1H), 7.59 (t, J = 7.6 Hz, 1H), 7.49 (t, J = 7.8 Hz, 1H), 7.36 (d, J = 7.6 Hz, 1H), 3.66 (t, J = 7.3 Hz, 2H), 3.12 (t, J = 6.8 Hz, 2H), 2.01 – 1.79 (m, 4H).[13]

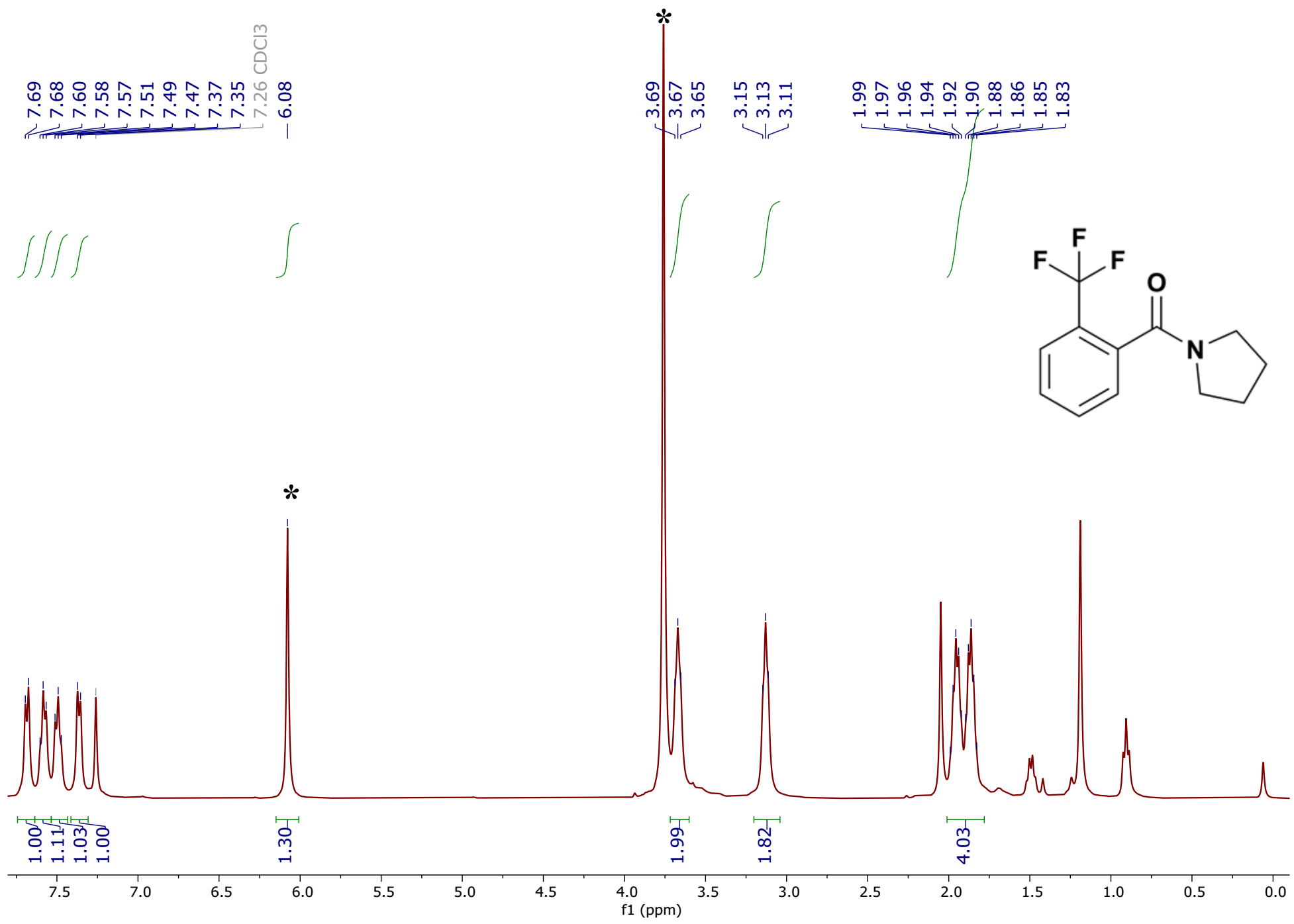

*Figure S23. ^{1}H NMR spectrum of the reaction mixture after acid wash of the synthesis of **15**. *Internal standard: 1,3,5-trimethoxybenzene.*

(4-methoxyphenyl)(pyrrolidin-1-yl)methanone (16): ^{1}H NMR (400 MHz, $CDCl_3$, δ): 7.49 (d, J = 8.4 Hz, 2H), 6.88 (d, J = 8.2 Hz, 2H), 3.82 (s, 3H), 3.79-3.50 (m, 4H), 2.01-1.72 (m, 4H).[13]

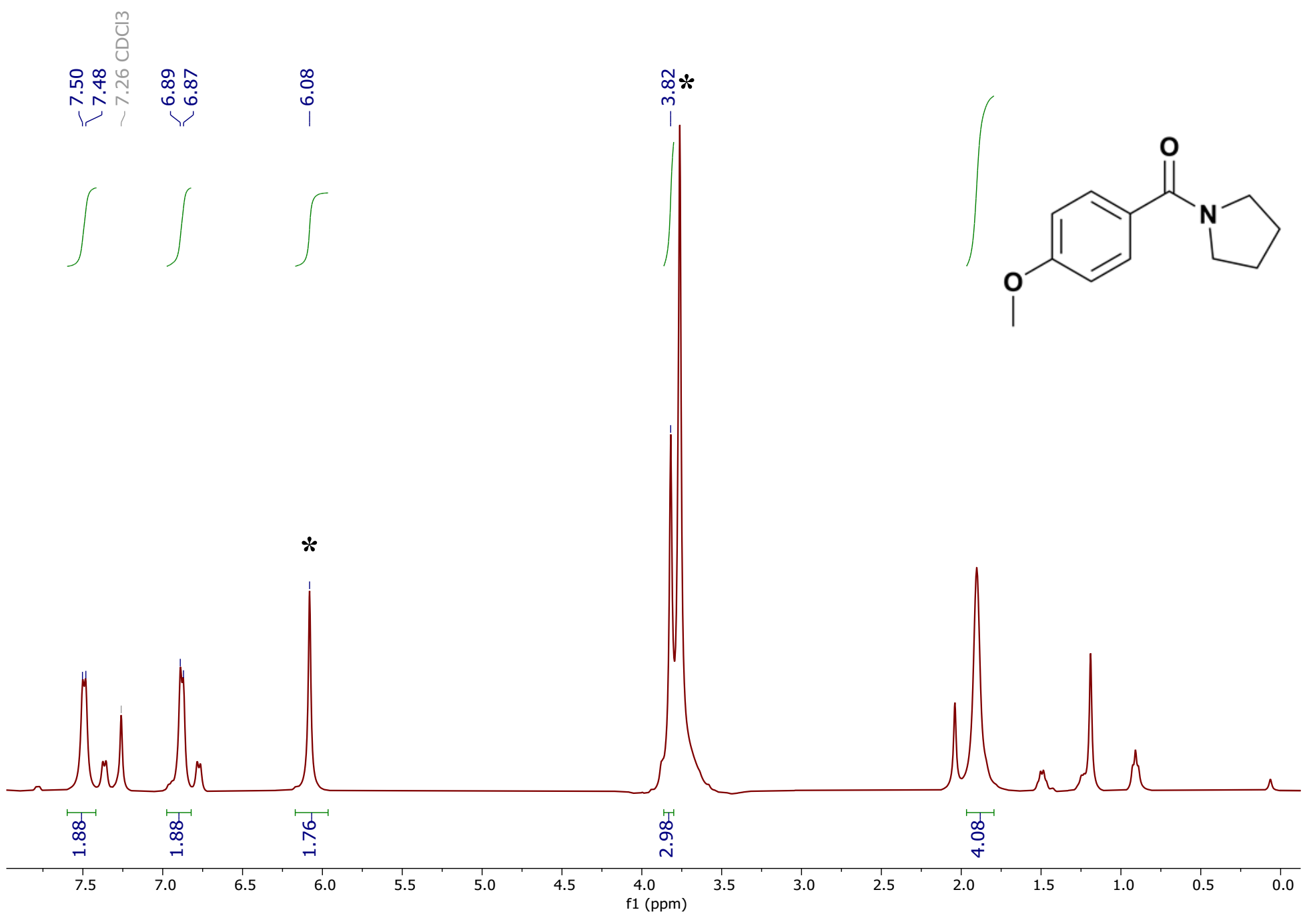

*Figure S24. ^{1}H NMR spectrum of the reaction mixture after acid wash of the synthesis of **16**. *Internal standard: 1,3,5-trimethoxybenzene.*

(3-methoxyphenyl)(pyrrolidin-1-yl)methanone (17): ^{1}H NMR (400 MHz, $CDCl_3$, δ): 7.39 – 7.26 (m, 1H), 7.08-7.03 (m, 2H), 6.93 (d, *J* = 8.4 Hz, 1H), 3.81 (s, 3H), 3.70-3.43 (m, 4H), 2.00-1.80 (m, 4H).[13]

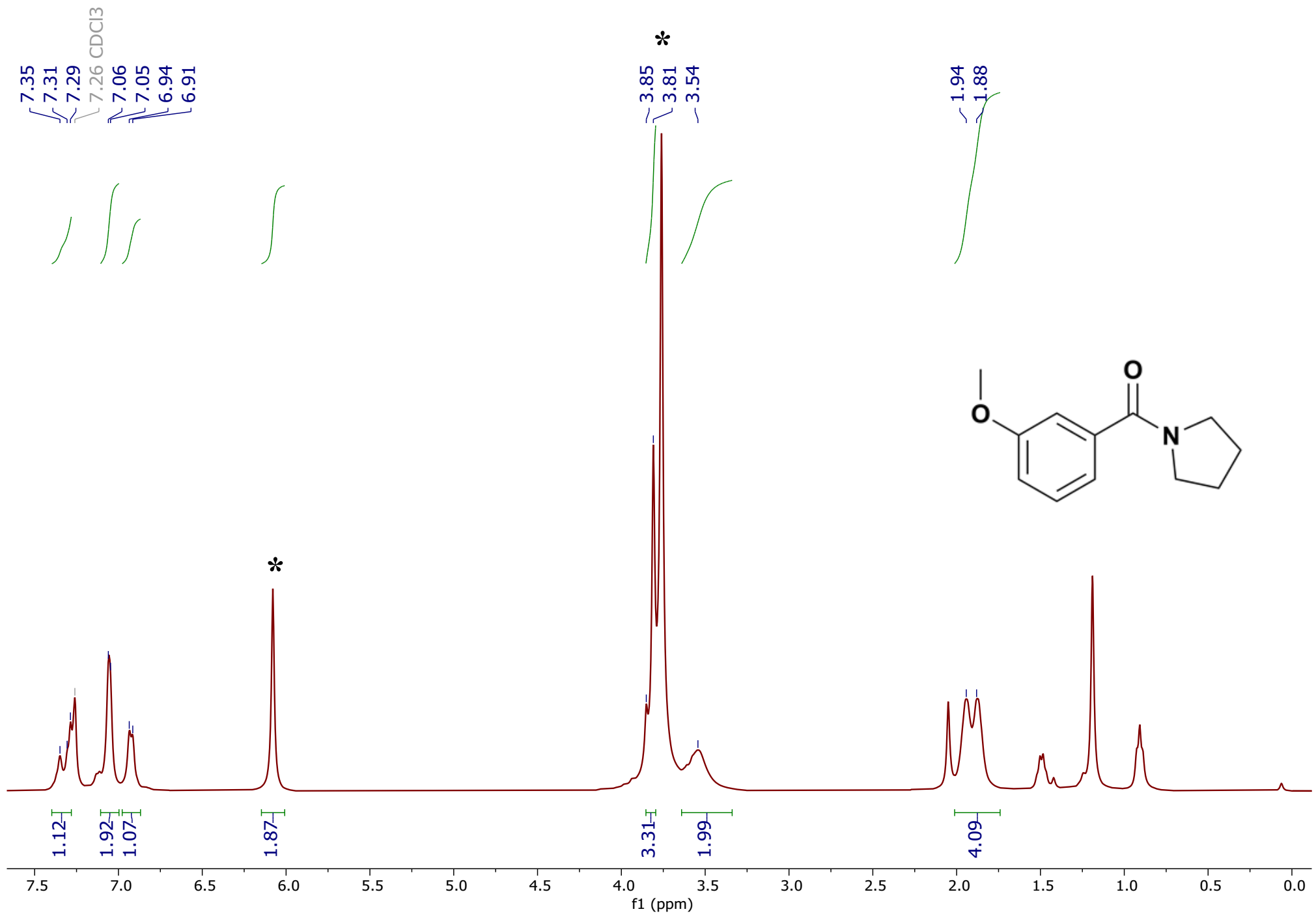

*Figure S25. ^{1}H NMR spectrum of the reaction mixture after acid wash of the synthesis of **17**. *Internal standard: 1,3,5-trimethoxybenzene.*

(2-methoxyphenyl)(pyrrolidin-1-yl)methanone (18): ^{1}H NMR (400 MHz, $CDCl_3$, δ): 7.33 (t, *J* = 7.9 Hz, 1H), 7.27-7.22 (m, 1H), 6.96 (t, *J* = 7.4 Hz, 1H), 6.90 (d, *J* = 8.4 Hz, 1H), 3.82 (s, 3H), 3.68 – 3.61 (m, 2H), 3.26 – 3.21 (m, 2H), 1.98 – 1.79 (m, 4H).[14]

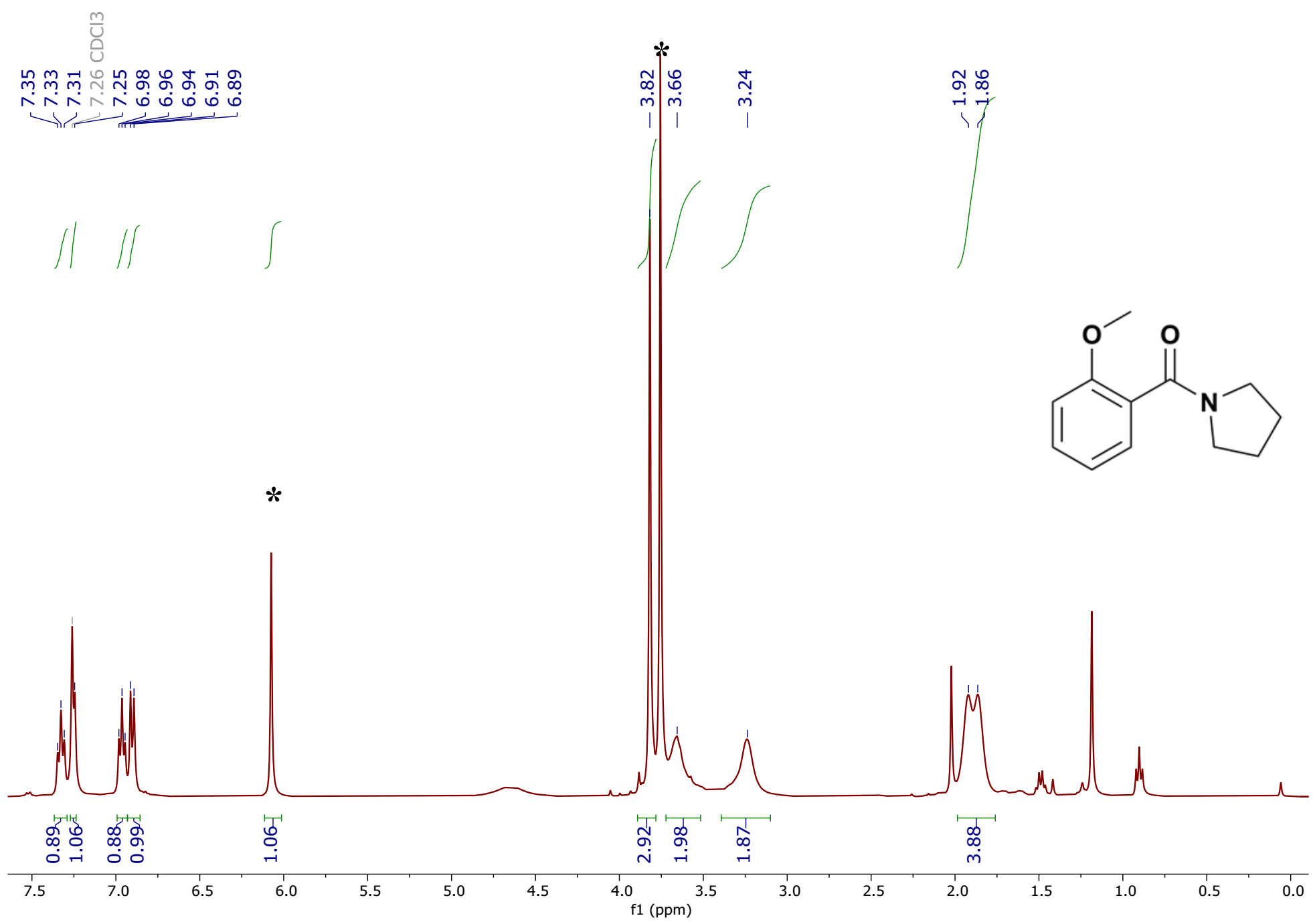

*Figure S26. ^{1}H NMR spectrum of the reaction mixture after acid wash of the synthesis of **18**. *Internal standard: 1,3,5-trimethoxybenzene.*

Furan-2-yl(pyrrolidin-1-yl)methanone (19): ^{1}H NMR (400 MHz, $CDCl_3$, δ): 7.51 – 7.47 (m, 1H), 7.06 – 7.01 (m, 1H), 6.50 – 6.44 (m, 1H), 3.97 – 3.81 (m, 2H), 3.72 – 3.53 (m, 2H), 2.05 – 1.79 (m, 4H).[15]

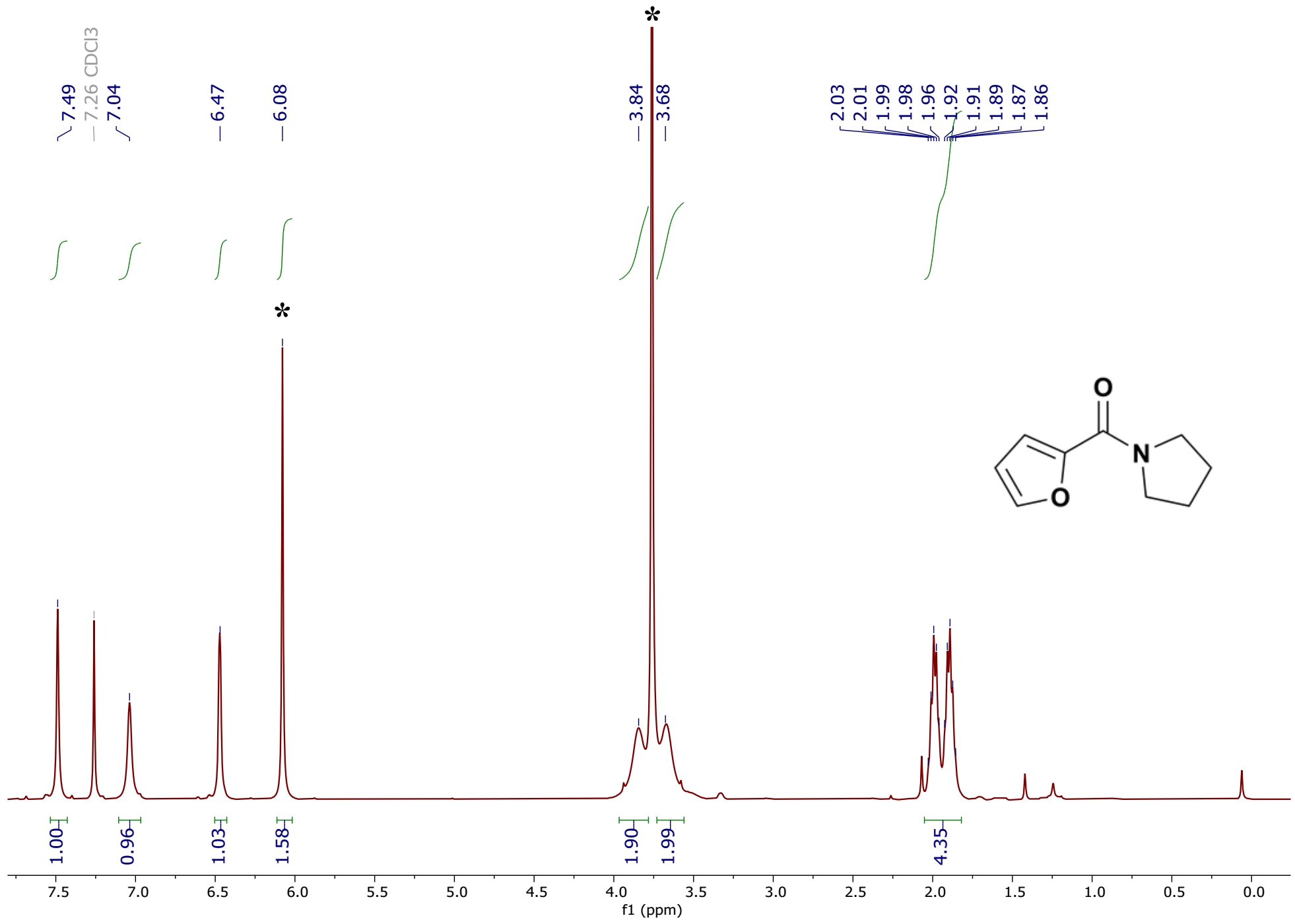

*Figure S27. ^{1}H NMR spectrum of the reaction mixture after acid wash of the synthesis of **19**. *Internal standard: 1,3,5-trimethoxybenzene.*

Pyridin-3-yl(pyrrolidin-1-yl)methanone (20): Reaction mixture was treated with 1M HCl, and treated with sodium hydrocarbonate until pH = 8. The product was extracted with ethyl acetate. ^{1}H NMR (400 MHz, $CDCl_3$, δ): 8.80 – 8.75 (m, 1H), 8.68 – 8.62 (m, 1H), 7.85 (d, J = 8.2 Hz, 1H), 7.34 (dd, J = 7.9, 4.8 Hz, 1H), 3.65 (t, J = 6.9 Hz, 2H), 3.44 (t, J = 6.6 Hz, 2H), 2.01 – 1.82 (m, 4H).[15]

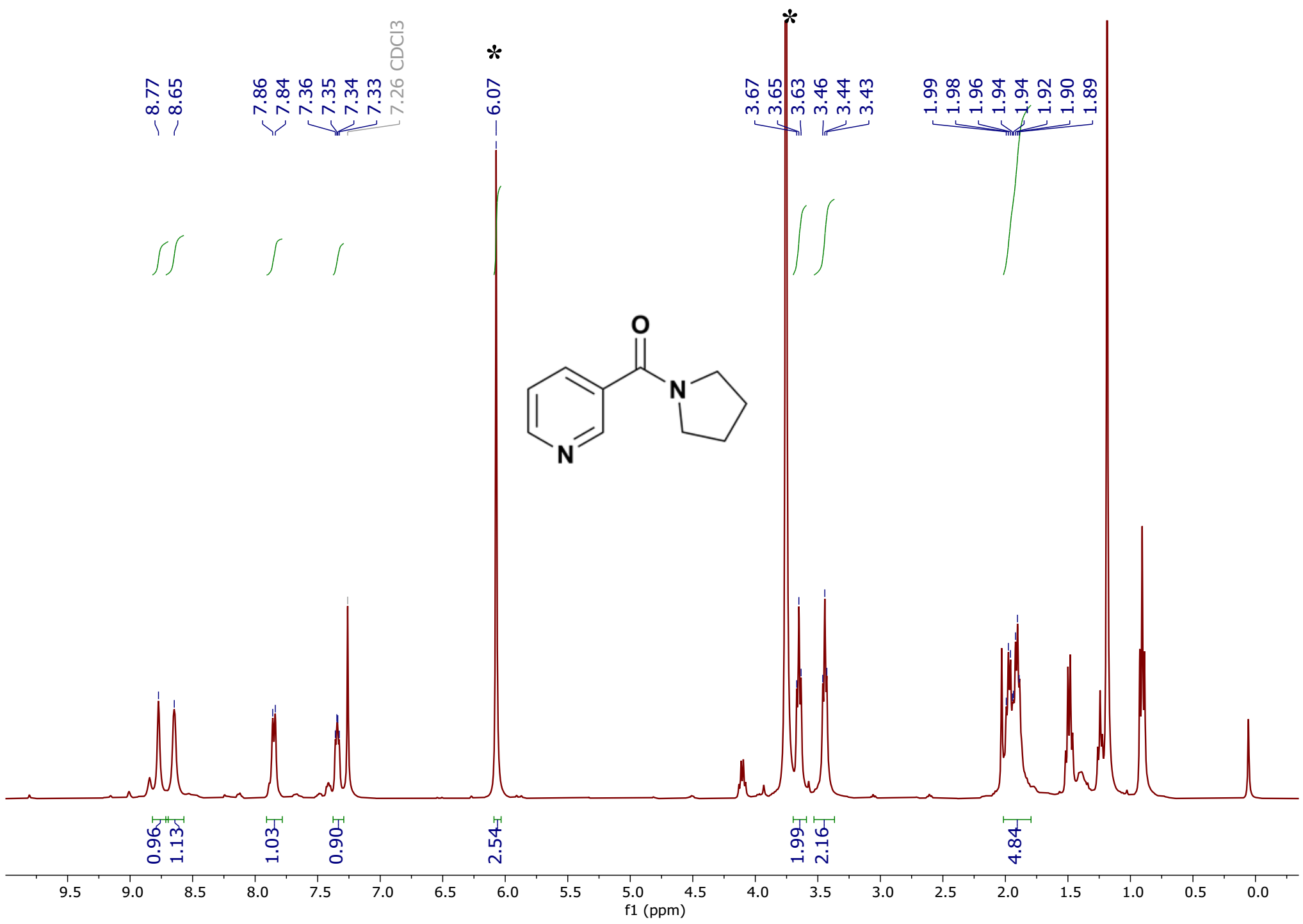

*Figure S28. ^{1}H NMR spectrum of the reaction mixture after acid wash of the synthesis of **20**. *Internal standard: 1,3,5-trimethoxybenzene.*

Pyrrolidin-1-yl(quinolin-5-yl)methanone (21): Reaction mixture was treated with 1M HCl, and treated with sodium hydrocarbonate until pH = 8. The product was extracted with ethyl acetate. ^{1}H NMR (400 MHz, $CDCl_3$, δ): 8.95 – 8.91 (m, 1H), 8.26 (d, *J* = 8.5 Hz, 1H), 8.12 (d, *J* = 10.6 Hz, 1H), 7.71 (dd, *J* = 8.5, 7.8 Hz, 1H), 7.56 – 7.50 (m, 1H), 7.48 – 7.40 (m, 1H), 3.78 (t, *J* = 7.0 Hz, 2H), 3.13 (t, *J* = 6.8 Hz, 2H), 2.00 (p, *J* = 6.9 Hz, 2H), 1.84 (p, *J* = 6.8 Hz, 2H).[16]

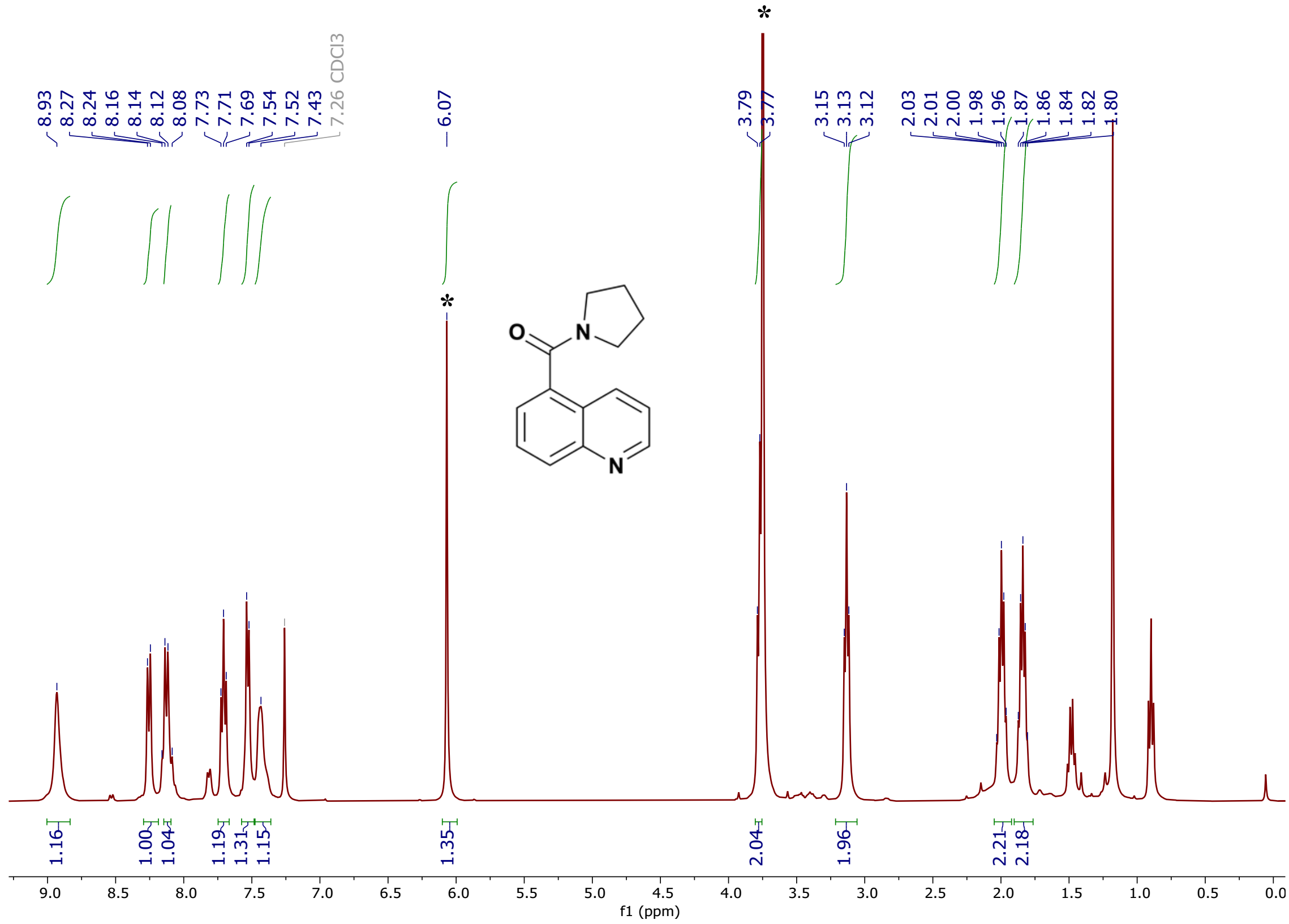

*Figure S29. ^{1}H NMR spectrum of the reaction mixture after acid wash of the synthesis of **21**. *Internal standard: 1,3,5-trimethoxybenzene.*

6.2. General procedure for alkoxycarbonylation reactions

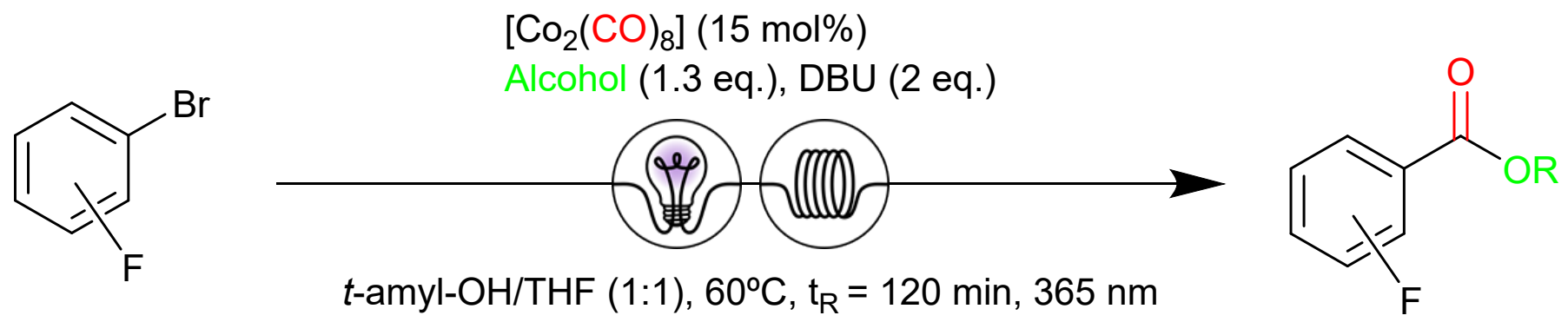

A stock solution of $[Co_2(CO)_8]$ in a 1:1 v/v mixture of *t*-amyl-OH/THF and a separate organic stock solution containing 2/4-fluorobromobenzene (1 eq, 0.25 M), the corresponding alcohol (1.3 equiv.), DBU (2 equiv.), and 1:1 v/v mixture of *t*-amyl-OH/THF were prepared and loaded into 50 mL syringes. The solutions were infused at a total flow rate of 0.084 ml/min (t_R = 120 minutes) and mixed prior to entering the photoreactor using a T-mixer (part number: P-714, supplier: VWR), this resulted in a 0.125 M concentration in 4-fluorobromobenzene, and a catalyst loading of 15 mol%. The temperature was set to 60ºC. Three 5 ml samples were collected sequentially after 1.5 x t_R, and yields determined using quantitative ^{19}F NMR spectroscopy using trifluorotoluene as internal standard. The signals of the products were assigned based on the values found in the literature and the isolated products.

Pentyl 4-fluorobenzoate (22): ^{1}H NMR (400 MHz, DMSO-d$_6$, δ): 8.06 – 7.96 (m, 2H), 7.40 – 7.29 (m, 2H), 4.25 (td, *J* = 6.6, 1.4 Hz, 2H), 1.69 (m, 2H), 1.43 – 1.25 (m, 4H), 0.88 (td, *J* = 7.2, 1.4 Hz, 3H). ^{19}F NMR (377 MHz, DMSO-d$_6$, δ): -106.15 (s, 1F).[17]

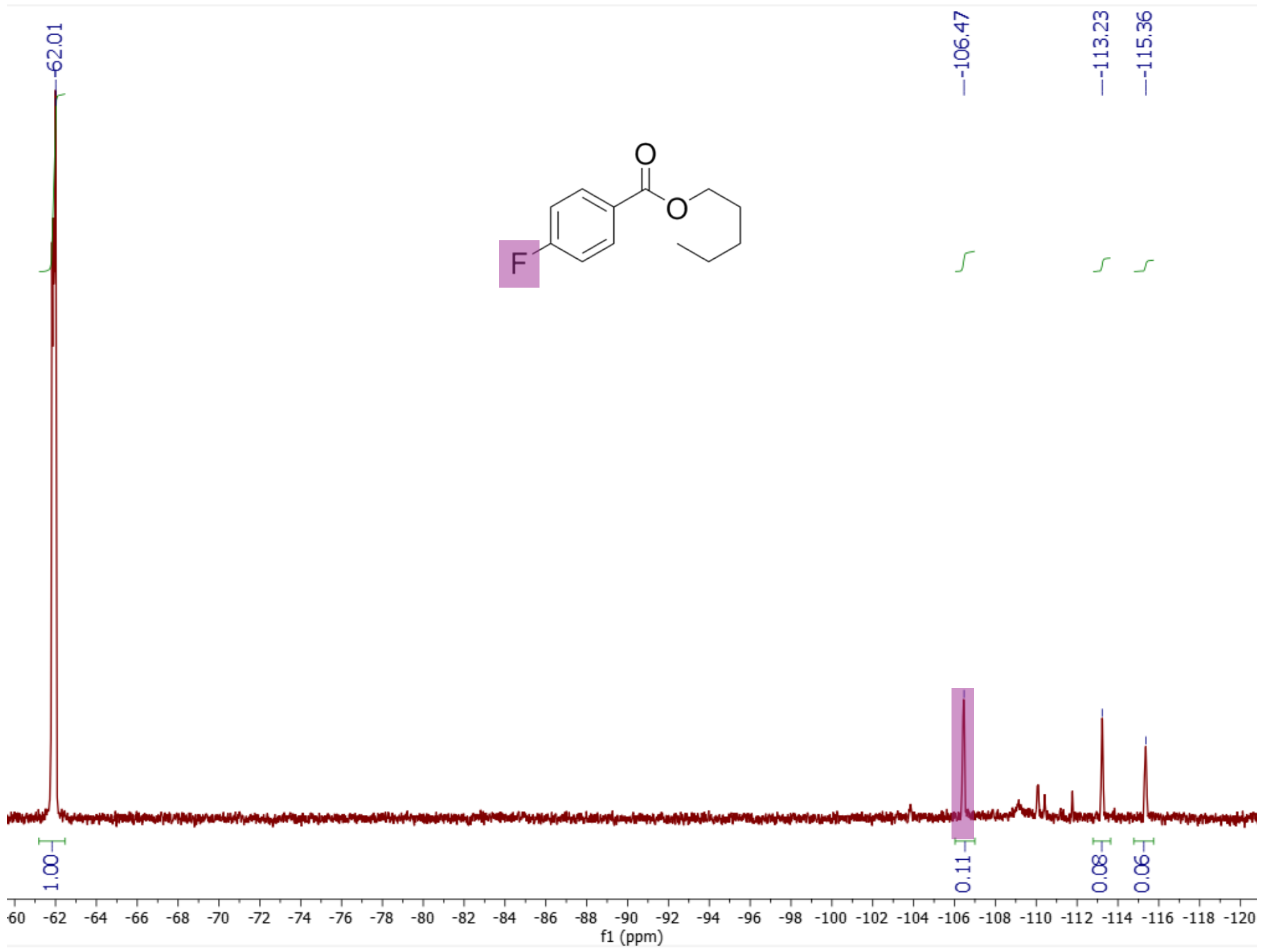

*Figure S30. ^{19}F NMR spectrum of the reaction mixture of the synthesis of **22**. Internal standard: trifluorotoluene (δ = -62.01 ppm in DMSO-d$_6$)*

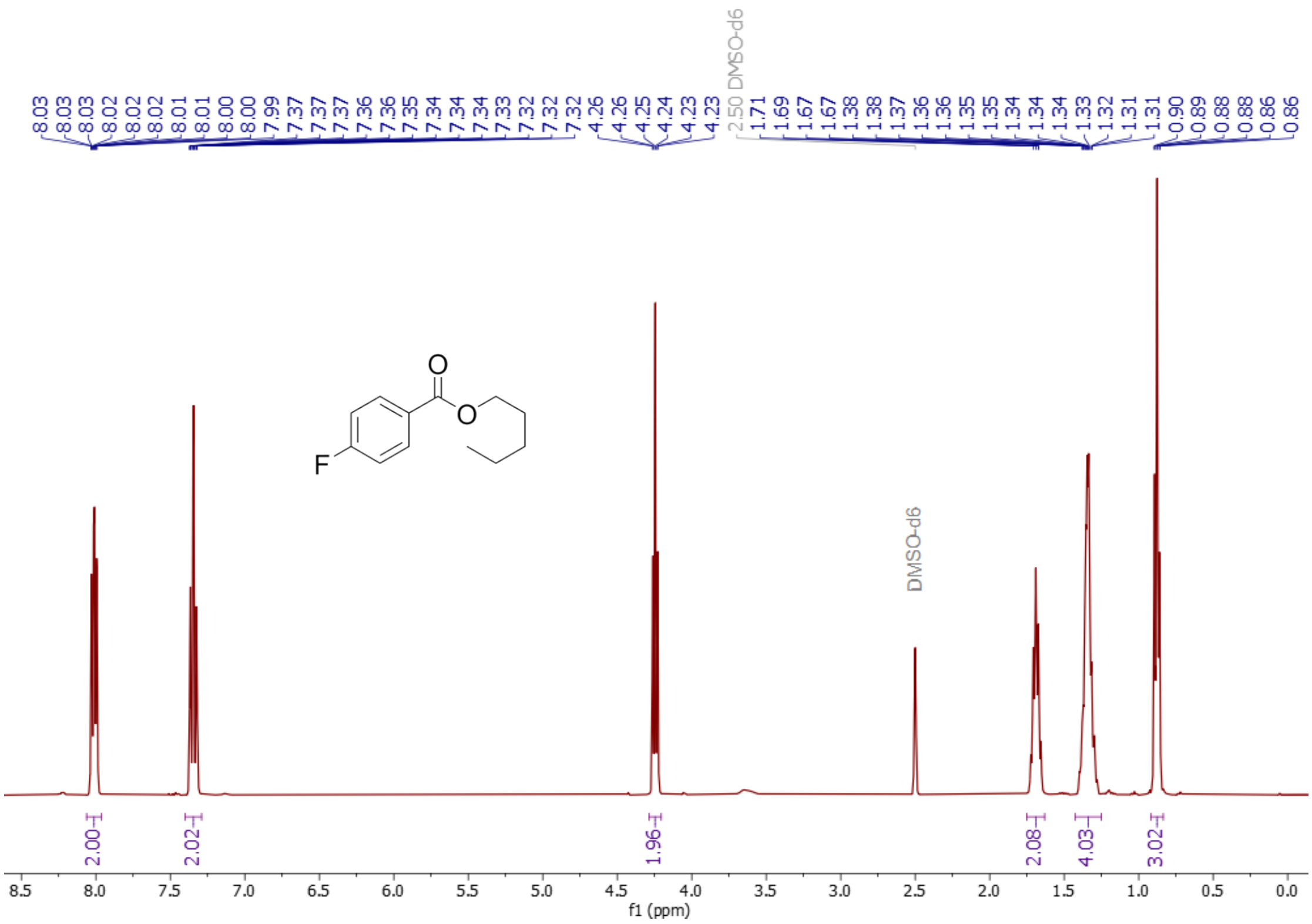

*Figure S31. ^{1}H NMR spectrum of isolated pentyl 4-fluorobenzoate **(22)** in DMSO-d$_6$.*

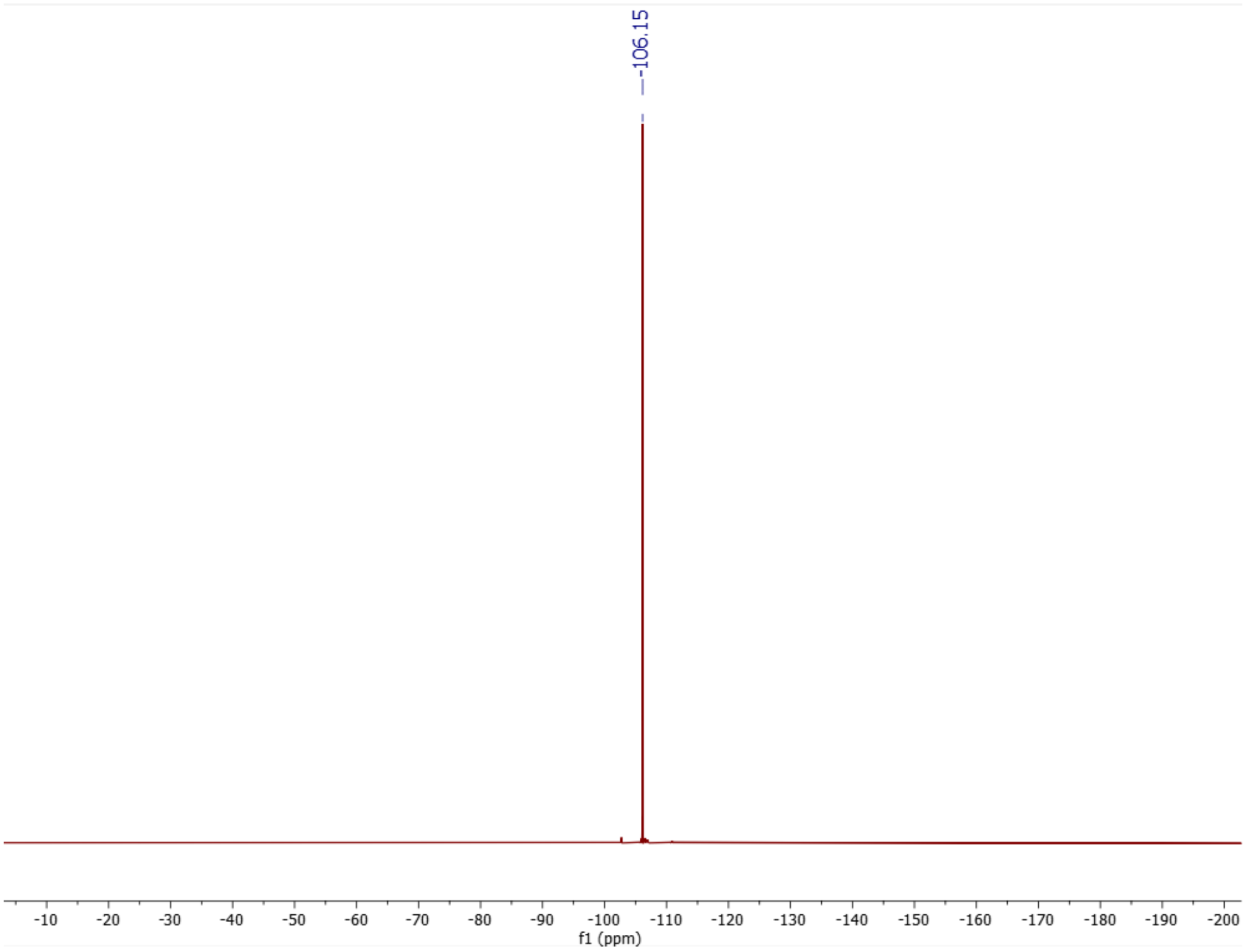

Figure S32. ^{19}F NMR spectrum of isolated pentyl 4-fluorobenzoate ***(22)*** *in DMSO-d_6.*

Pentyl 2-fluorobenzoate (23): ^{1}H NMR (400 MHz, DMSO-d_6, δ) 7.87 (tdd, J = 7.9, 1.9, 0.9 Hz, 1H), 7.72 – 7.61 (m, 1H), 7.38 – 7.28 (m, 2H), 4.26 (td, J = 6.6, 1.5 Hz, 2H), 1.67 (m, J = 6.3 Hz, 2H), 1.33 (m, 4H), 0.87 (td, J = 7.1, 1.9 Hz, 3H). ^{19}F NMR (377 MHz, DMSO-d_6, δ): -110.57 (s, 1F).[17]

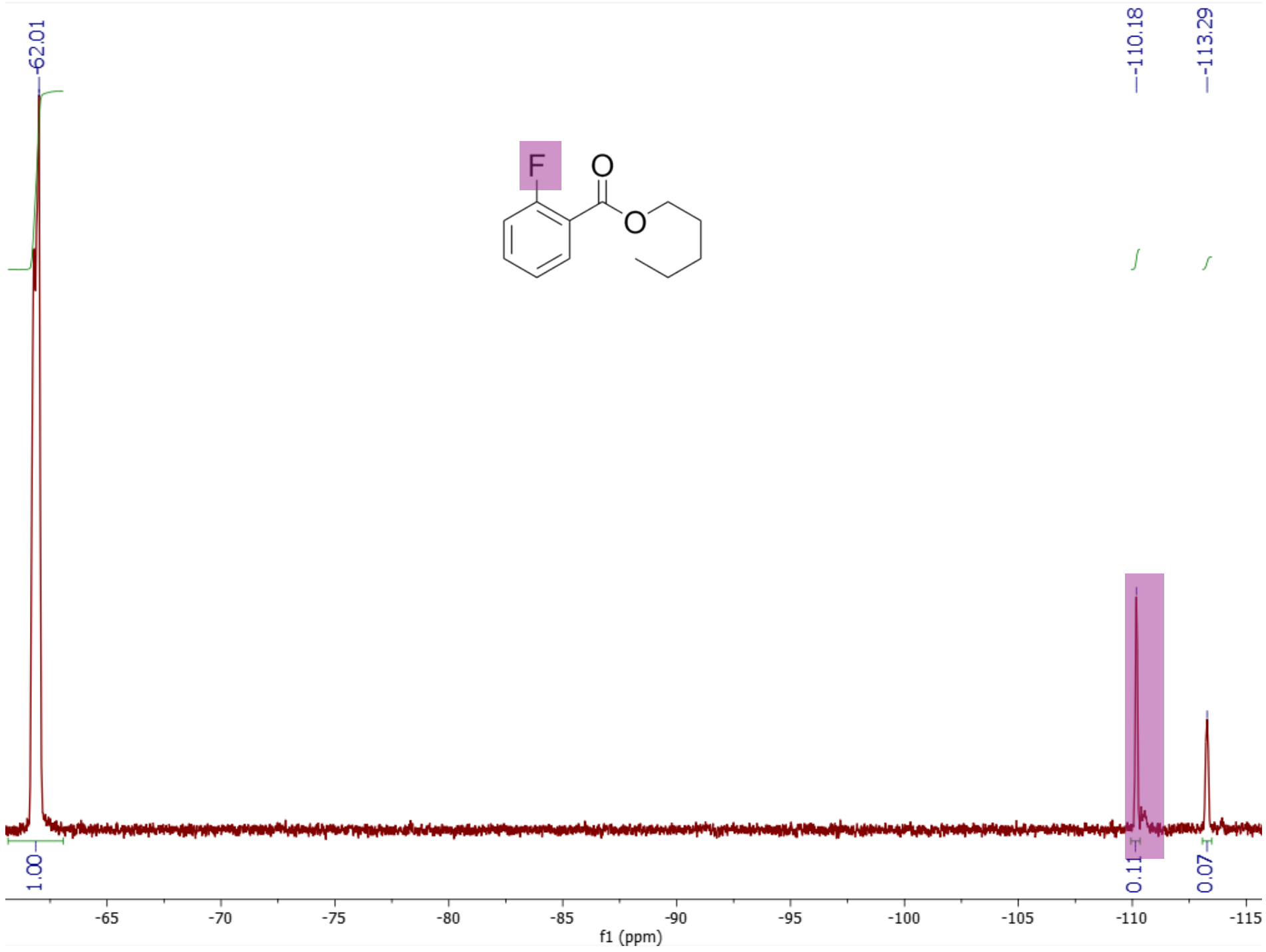

*Figure S33. ^{19}F NMR spectrum of the reaction mixture of the synthesis of **23**. Internal standard: trifluorotoluene (δ = -62.01 ppm in DMSO-d_6)*

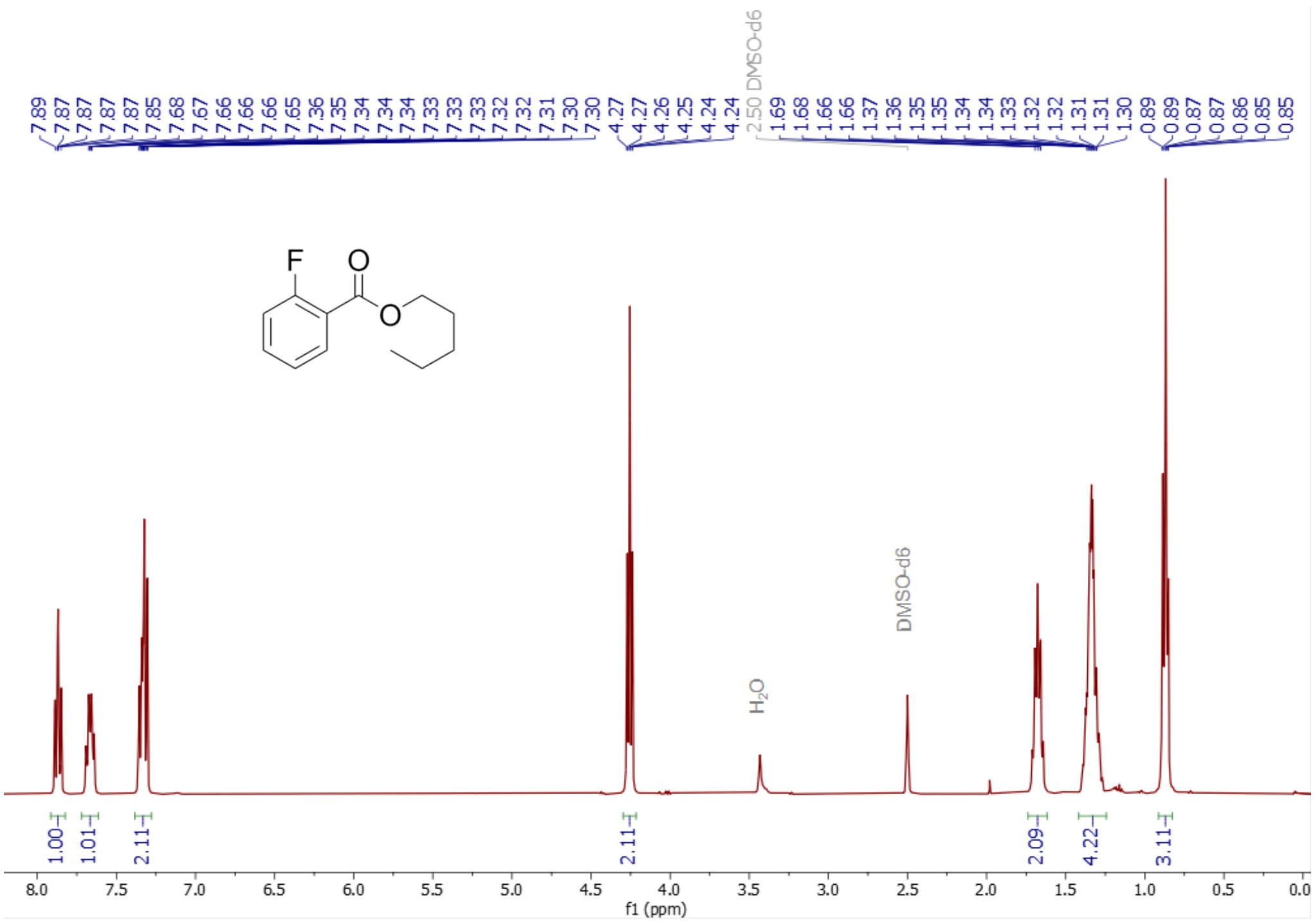

*Figure S34. ^{1}H NMR spectrum of isolated pentyl 2-fluorobenzoate **(23)** in DMSO-d_6.*

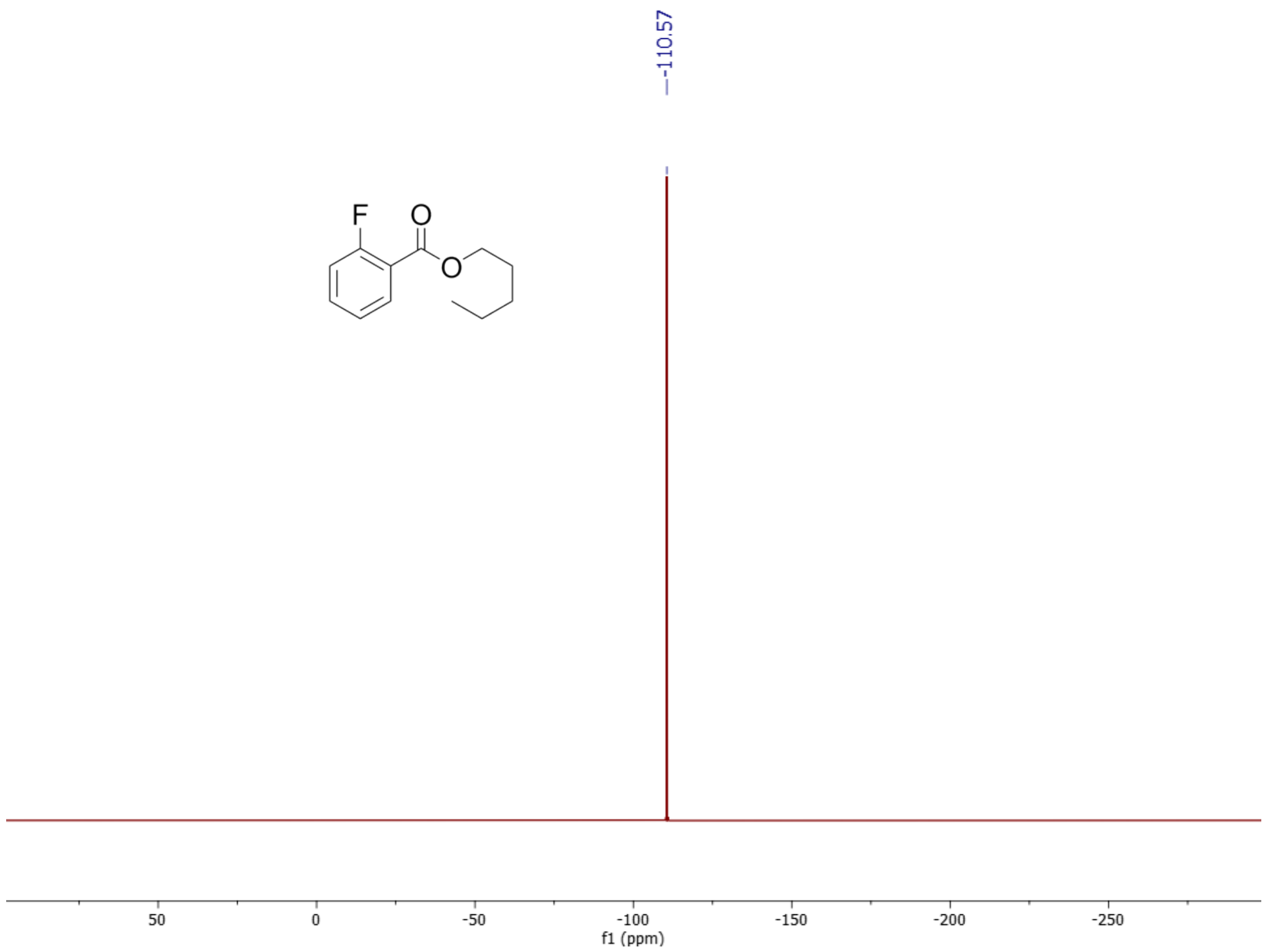

Figure S35. ^{19}F NMR spectrum of isolated pentyl 2-fluorobenzoate ***(23)*** *in DMSO-d$_6$.*

Cyclohexyl o-fluorobenzoate (24): ^{1}H NMR (400 MHz, DMSO-d_6, δ) 7.87 (m,, 1H), 7.72 – 7.62 (m, 1H), 7.39 – 7.29 (m, 2H), 4.96 (tt, *J* = 8.2, 3.7 Hz, 1H), 1.86 (m, 2H), 1.71 (m, 2H), 1.61 – 1.26 (m, 6H). ^{13}C NMR (101 MHz, DMSO-d_6, δ): 162.9 (d, *J* = 3.5 Hz), 160.9 (d, *J* = 257.2 Hz), 134.9 (d, *J* = 8.9 Hz), 131.6, 124.5 (d, *J* = 3.9 Hz), 118.8 (d, *J* = 10.2 Hz), 117.0 (d, *J* = 22.0 Hz), 72.8, 30.8 (2C), 24.9, 22.8 (2C). ^{19}F NMR (377 MHz, DMSO-d_6, δ): -110.69 (s, 1F). HRMS (ES-TOF): $^{m}/_{z}$ calc for $C_{13}H_{16}O_2F$: 223.1134 [**15**+H]$^+$, found 223.1126.

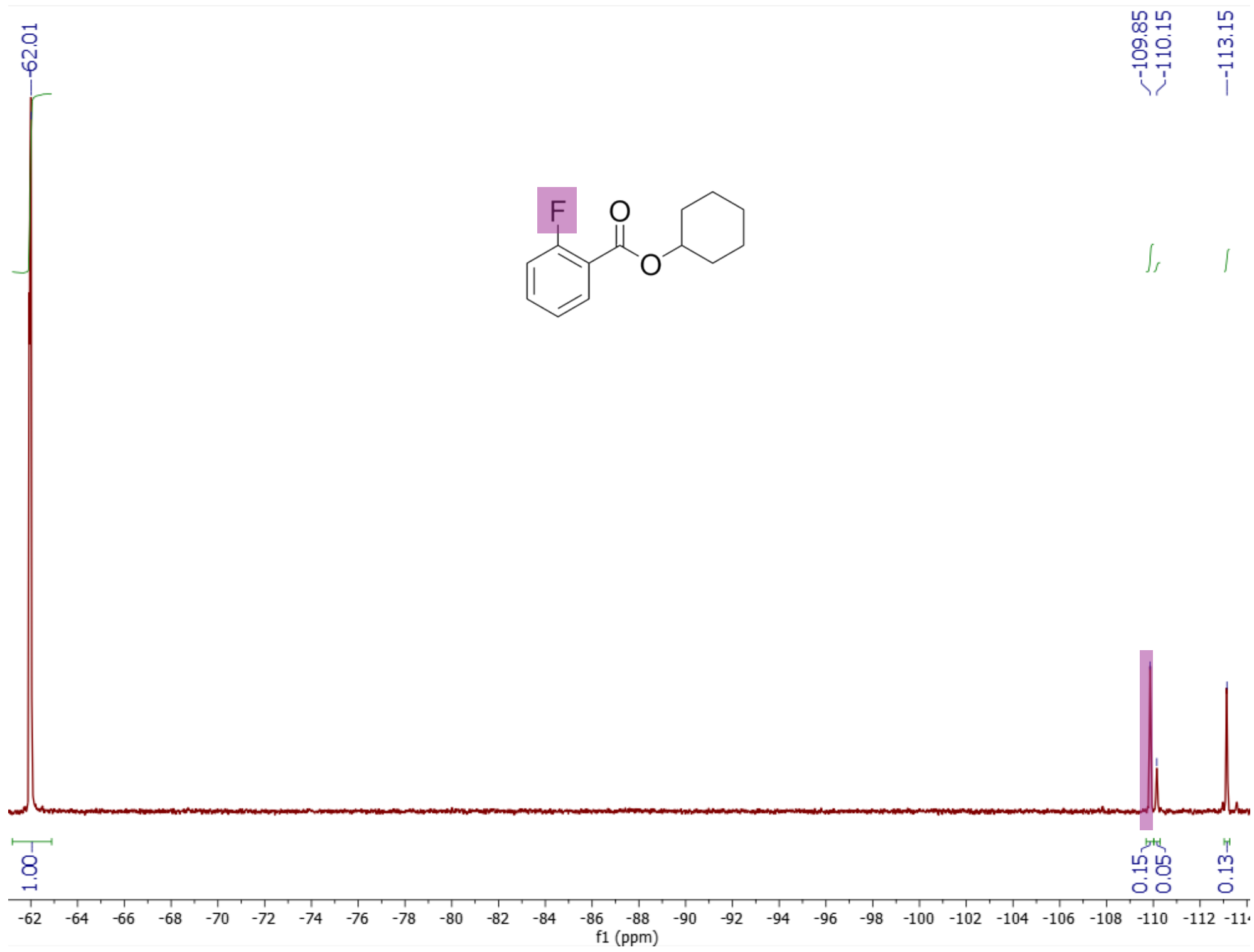

*Figure S36. ^{19}F NMR spectrum of the reaction mixture of the synthesis of **24**. Internal standard: trifluorotoluene (δ = -62.01 ppm in DMSO-d_6)*

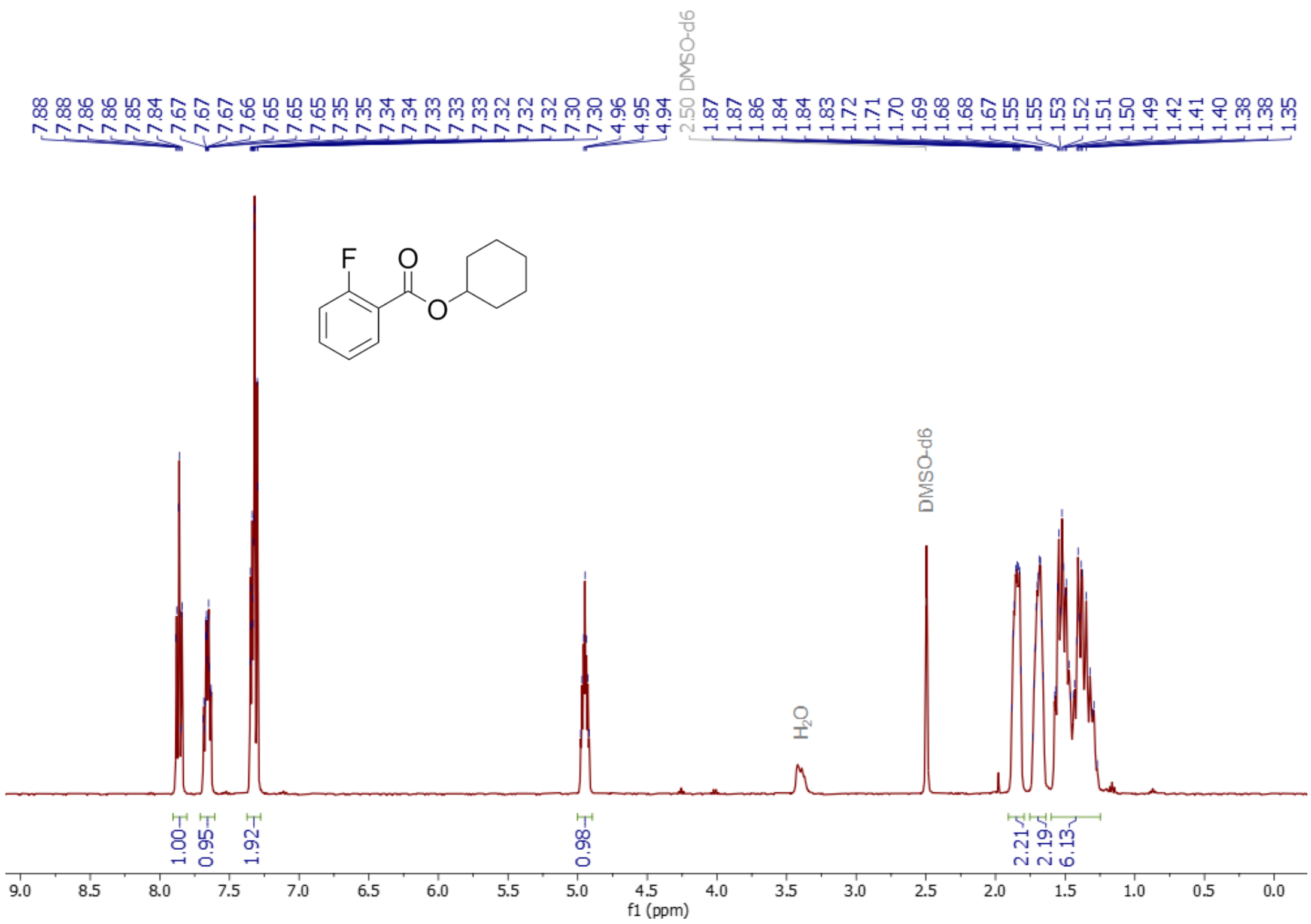

*Figure S37. ^{1}H NMR spectrum of isolated cyclohexyl o-fluorobenzoate **(24)** in DMSO-d_6.*

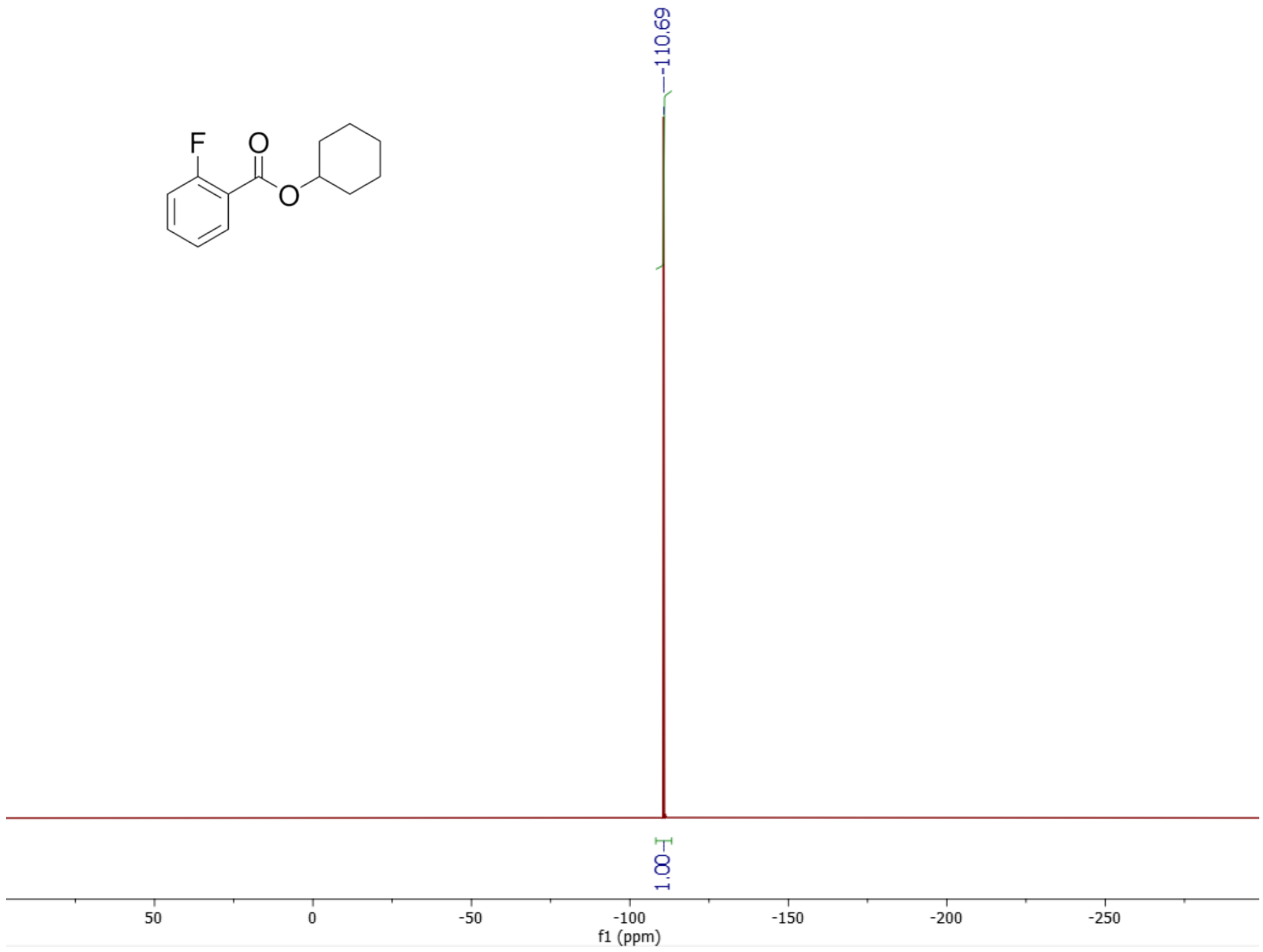

*Figure S38. ^{19}F NMR spectrum of isolated cyclohexyl o-fluorobenzoate **(24)** in DMSO-d_6.*

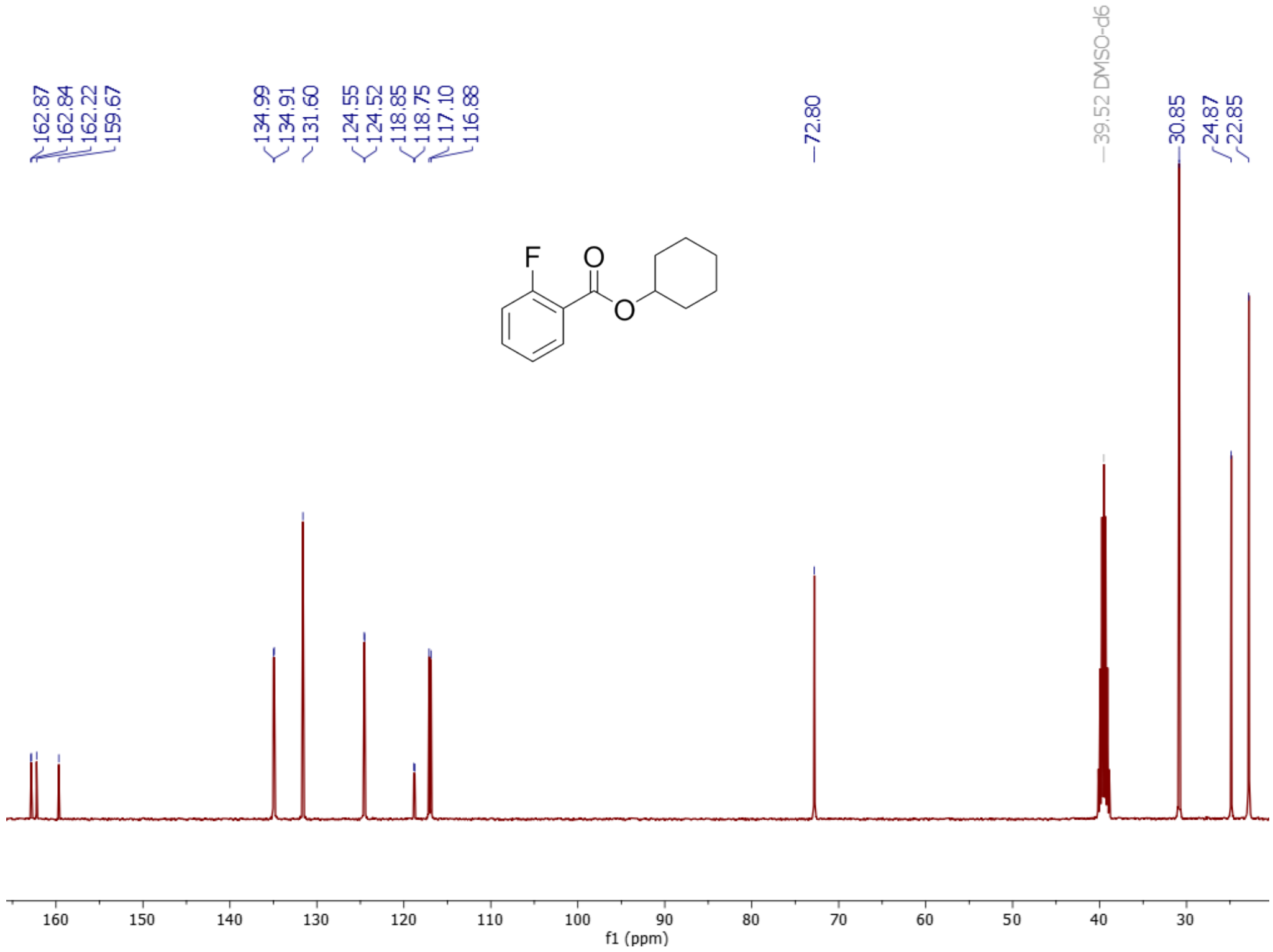

*Figure S39. ^{13}C NMR spectrum of isolated cyclohexyl o-fluorobenzoate **(24)** in DMSO-d_6.*

Tert-butyl 2-fluorobenzoate (25): ^{19}F NMR (377 MHz, DMSO-d_6, δ): -110.72 (s, 1F).[18]

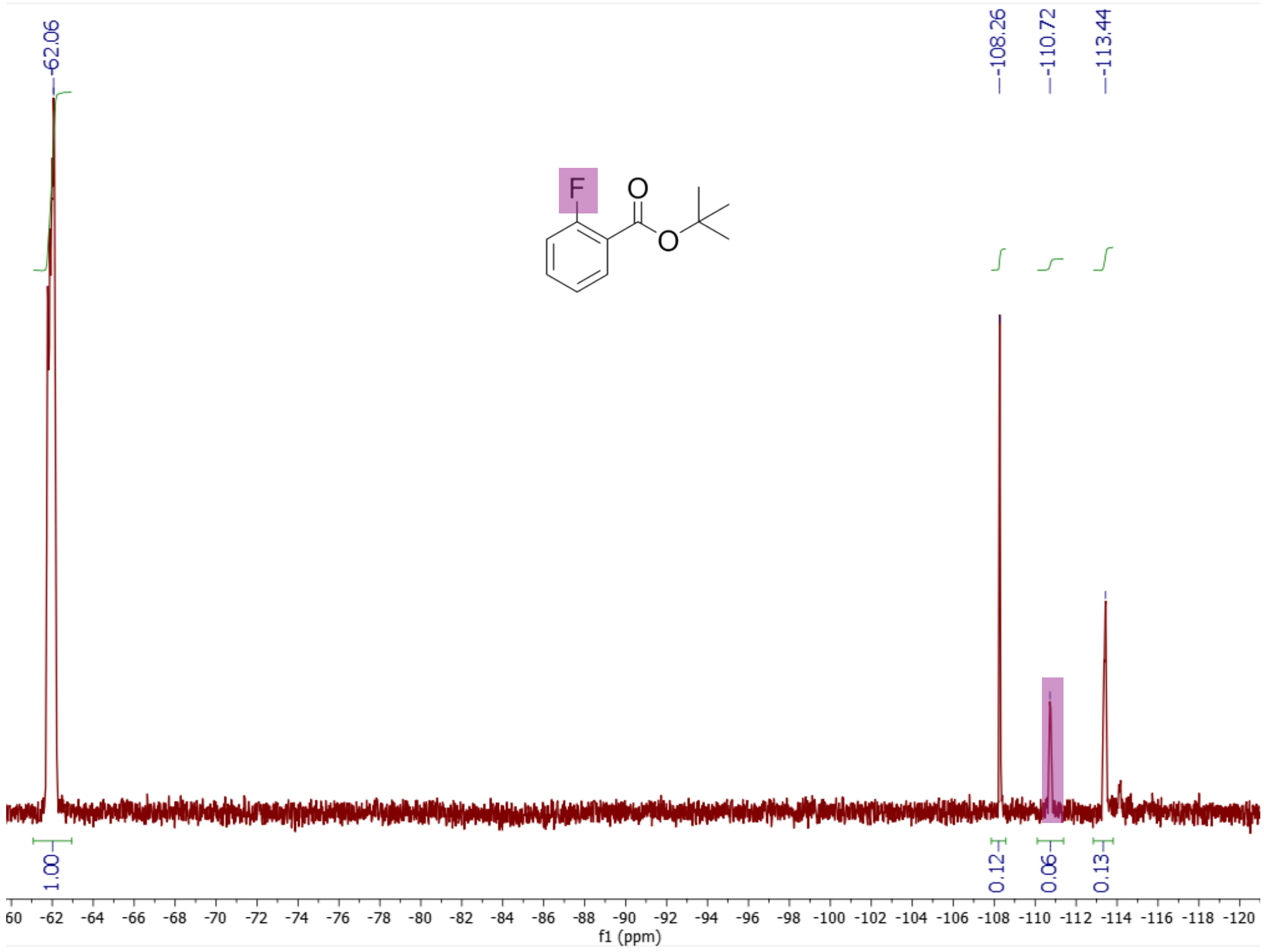

*Figure S40. ^{19}F NMR spectrum of the reaction mixture of the synthesis of **25**. Internal standard: trifluorotoluene (δ = -62.01 ppm in DMSO-d_6).*

Tert-butyl 2-fluorobenzoate (26): ^{19}F NMR (377 MHz, DMSO-d_6, δ): -108.51 (s, 1F).[19]

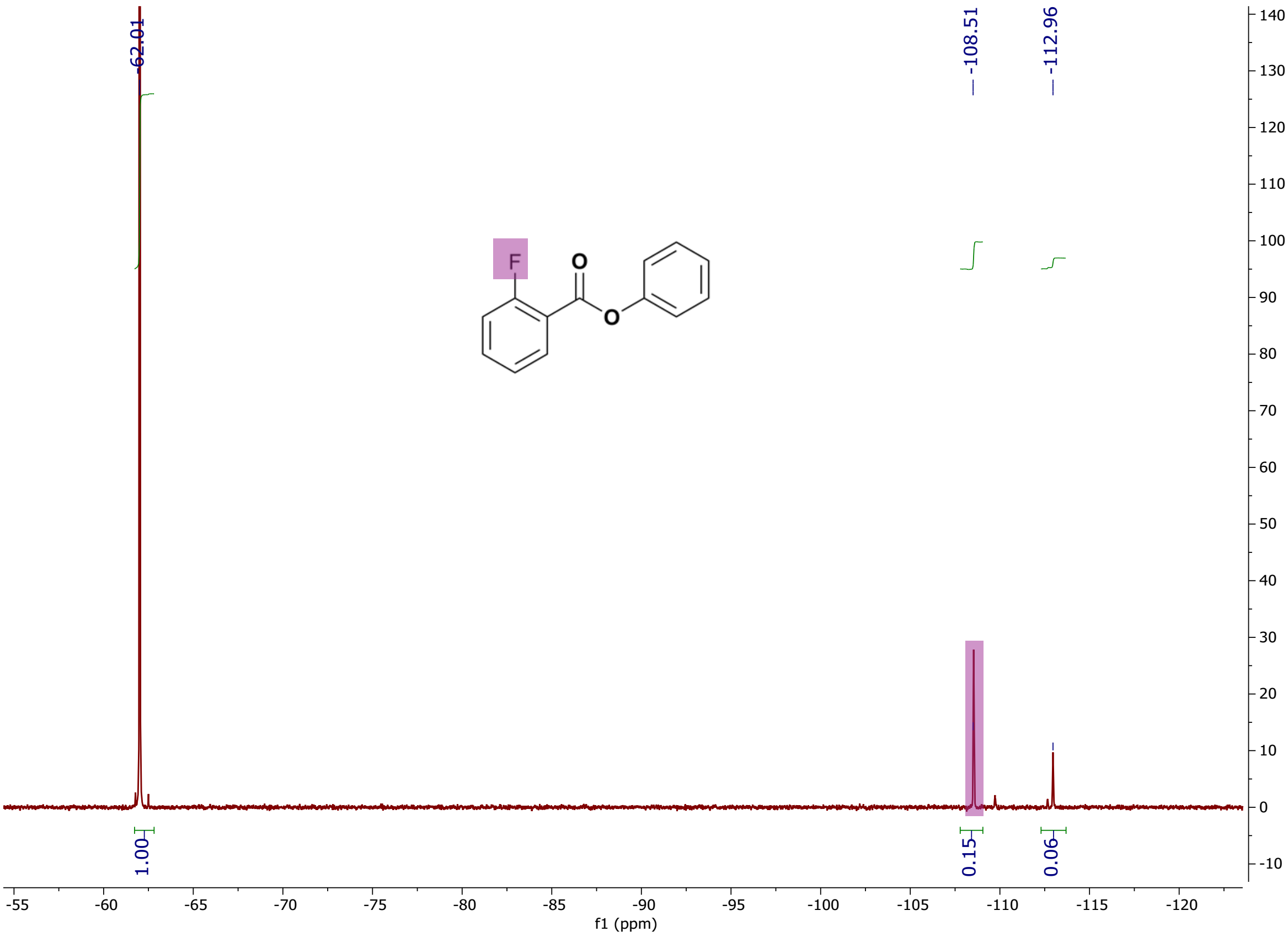

*Figure S41. ^{19}F NMR spectrum of the reaction mixture of the synthesis of **26**. Internal standard: trifluorotoluene (δ = -62.01 ppm in DMSO-d_6).*

7. Scale-up and in-line extraction in flow

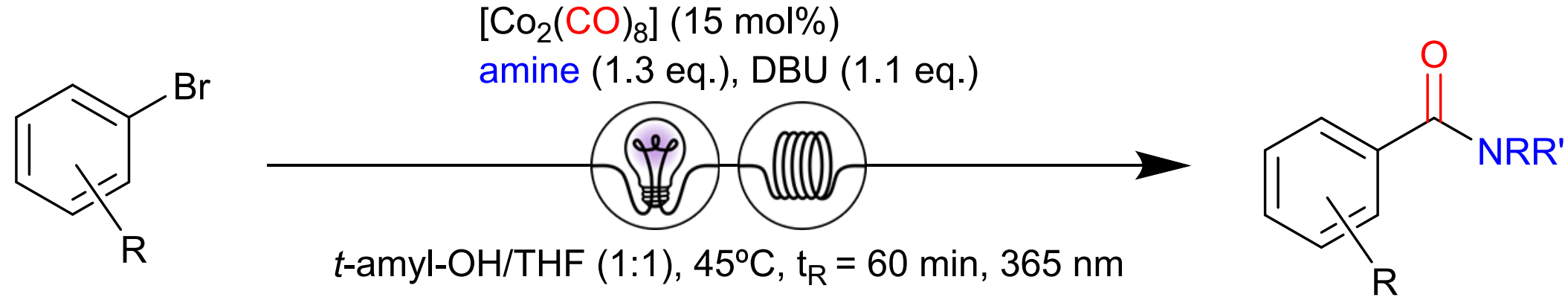

The optimised conditions were used for the scale-up reactions (see reaction scheme), using a 0.25 M concentration solution of the corresponding aryl bromide. A photograph of the reactor set-up is shown in Figure S42.

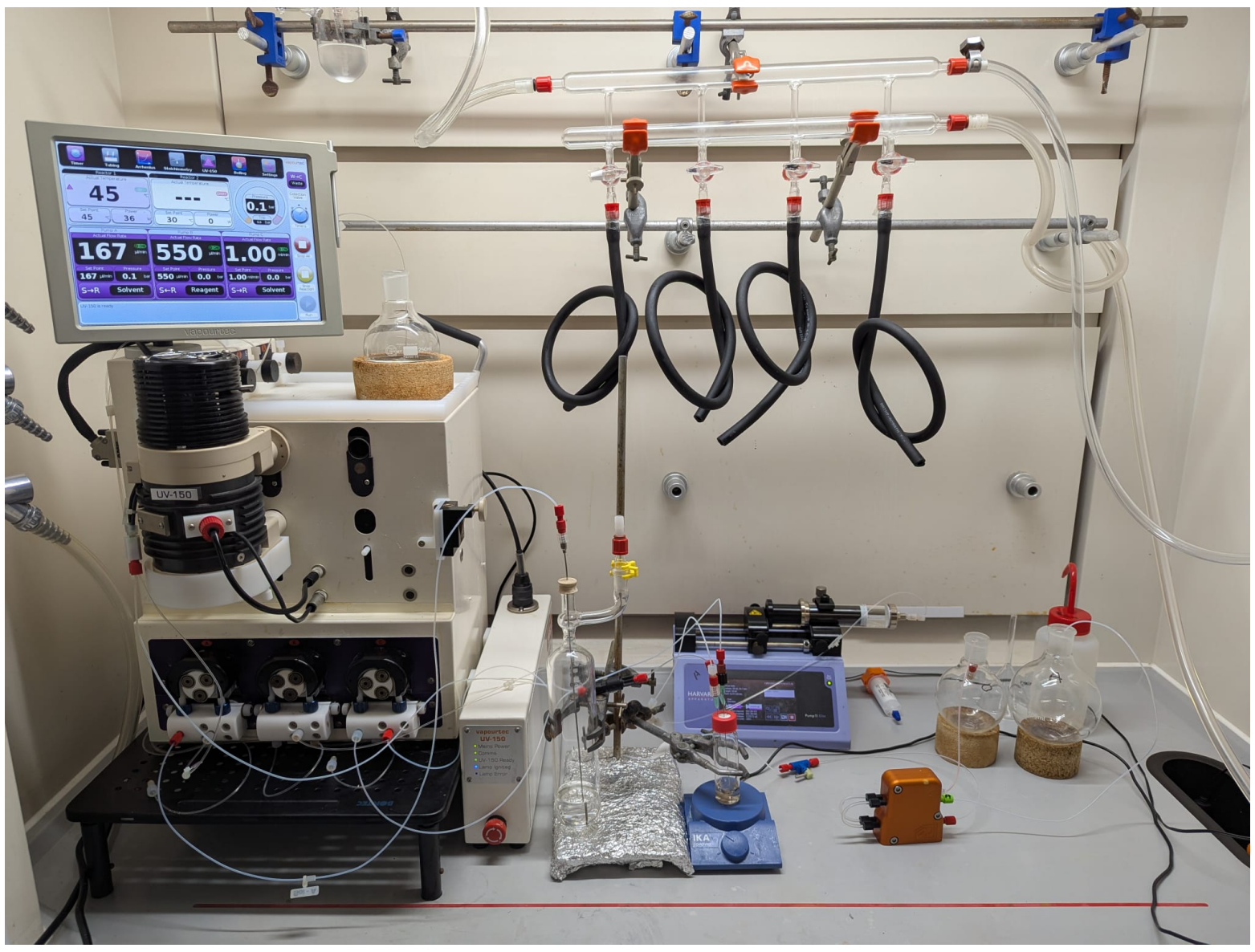

Figure S42. Set-up for the scale-up experiments

The reaction mixture was prepared and stored under nitrogen in a Schlenk tube, containing all organic reagents and catalyst. The reaction mixture was pumped using the Vapourtec peristaltic pump at 0.167 ml/min (t_R = 60 minutes) into the photoreactor. At the reactor outlet, the reaction effluent was mixed with an aqueous solution of 1M HCl (flow rate: 0.277 ml/min). An immediate change in the colour of the solution from dark blue to clear yellow was observed. The mixture was retained in a collection vial, into which EtOAc was pumped at a 0.550 ml/min and stirred vigorously, enabling organic and aqueous phases to mix. A needle inserted to the bottom of this vial and connected to a third peristaltic pump that was used to pump the mixture at 1 ml/min through a Zaiput membrane separator facilitating in-line separation. The aqueous stream was found to contain traces of organic phase that could be extracted manually to further improve yield. The organic fraction was concentrated in vacuo at 50ºC to yield the product. No further purification was conducted unless otherwise stated.

(4-fluorophenyl)(piperidino)methanone (1): Yellow oil, 1.742 g (8.41 mmol) after five-hour collection (Yield: 67%). ^{1}H NMR (400 MHz, $CDCl_3$) δ 7.38 (dd, *J* = 8.4, 5.3 Hz, 2H), 7.06 (m, 2H), 3.58 (m, 4H), 1.74 – 1.46 (m, 6H). NMR signals are consistent with literature.[2] ^{19}F NMR (377 MHz, $CDCl_3$, δ): -110.89 (s, 1F). **4,4′-**

Difluorobenzophenone: about 10% is also synthesised under this reaction conditions. NMR signals are consistent with literature.[20] ^{1}H NMR (400 MHz, $CDCl_3$, δ): 7.86 – 7.76 (m, 4H), 7.16 (m, 4H). ^{19}F NMR (377 MHz, $CDCl_3$, δ): -105.70 (s, 1F).

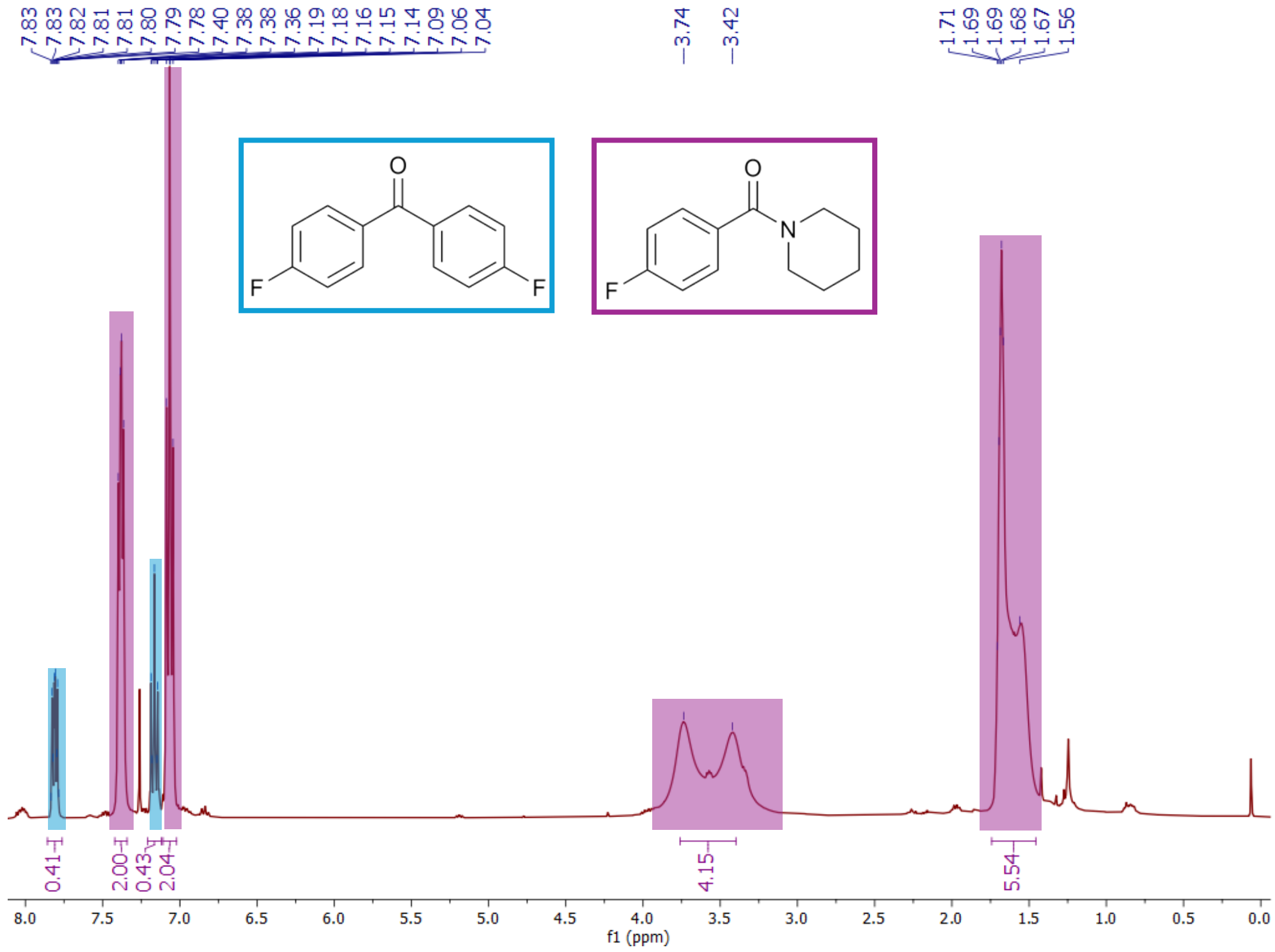

*Figure S43. ^{1}H NMR spectrum of (4-fluorophenyl)(piperidino)methanone (**1**) + 4,4'-Difluorobenzophenone*

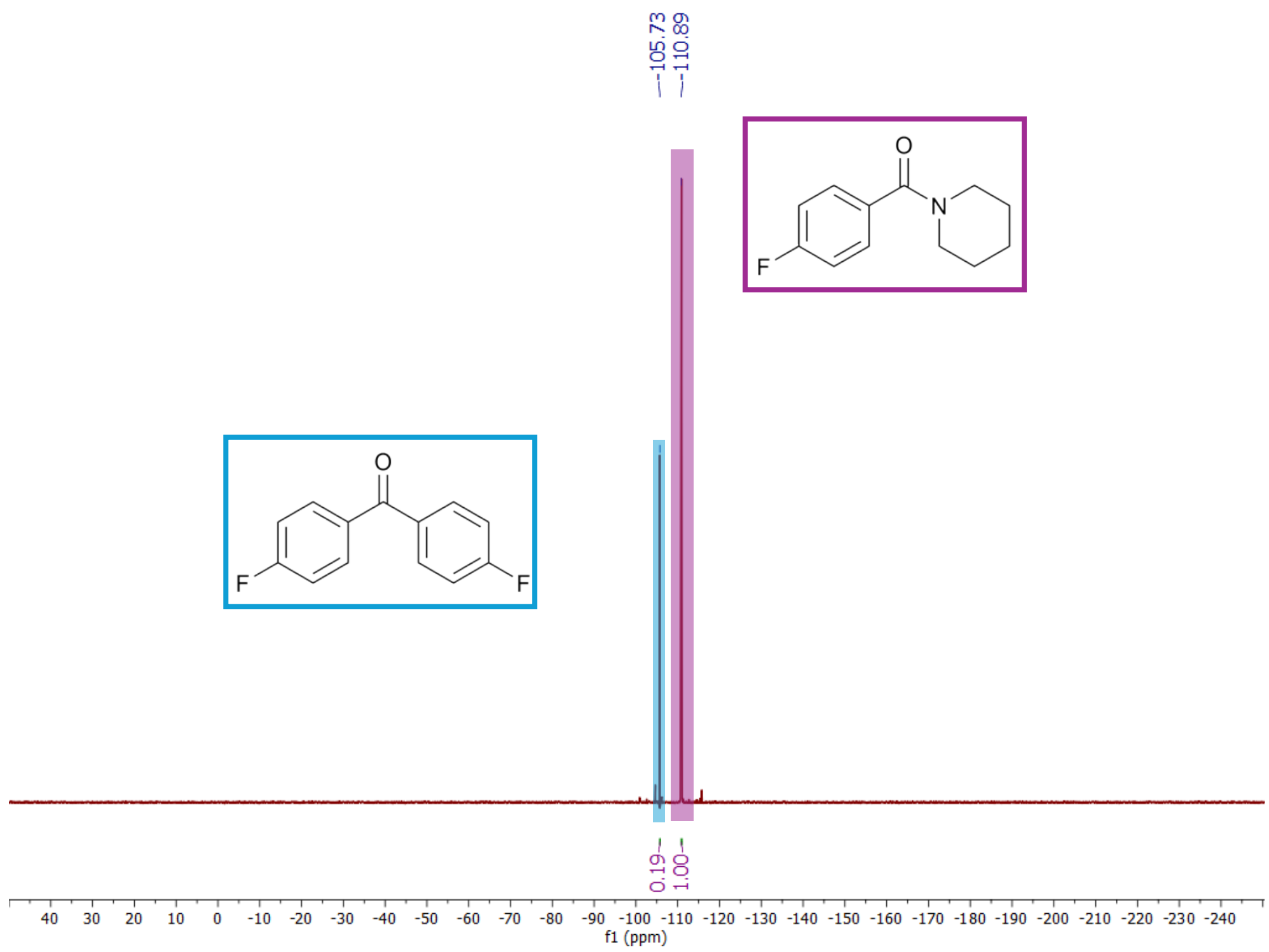

*Figure S44. ^{19}F NMR spectrum of (4-fluorophenyl)(piperidino)methanone (**1**) + 4,4'-Difluorobenzophenone*

(2-Fluorophenyl)(1-piperidinyl)methanone (7): Yellow oil, 1.086 g (5.24 mmol) after three hours collection (Yield: 70%). NMR spectra are consistent with literature.[10] ^{1}H NMR (400 MHz, $CDCl_3$, δ): 7.38 – 7.33 (m, 2H), 7.18 (pst, J = 7.4 Hz, 1H), 7.08 (pst, J = 8.9 Hz, 1H), 3.75 (m, 2H), 3.26 (s, 2H), 1.69 – 1.50 (m, 6H). ^{19}F NMR (377 MHz, $CDCl_3$, δ): -115.27 (s, 1F).

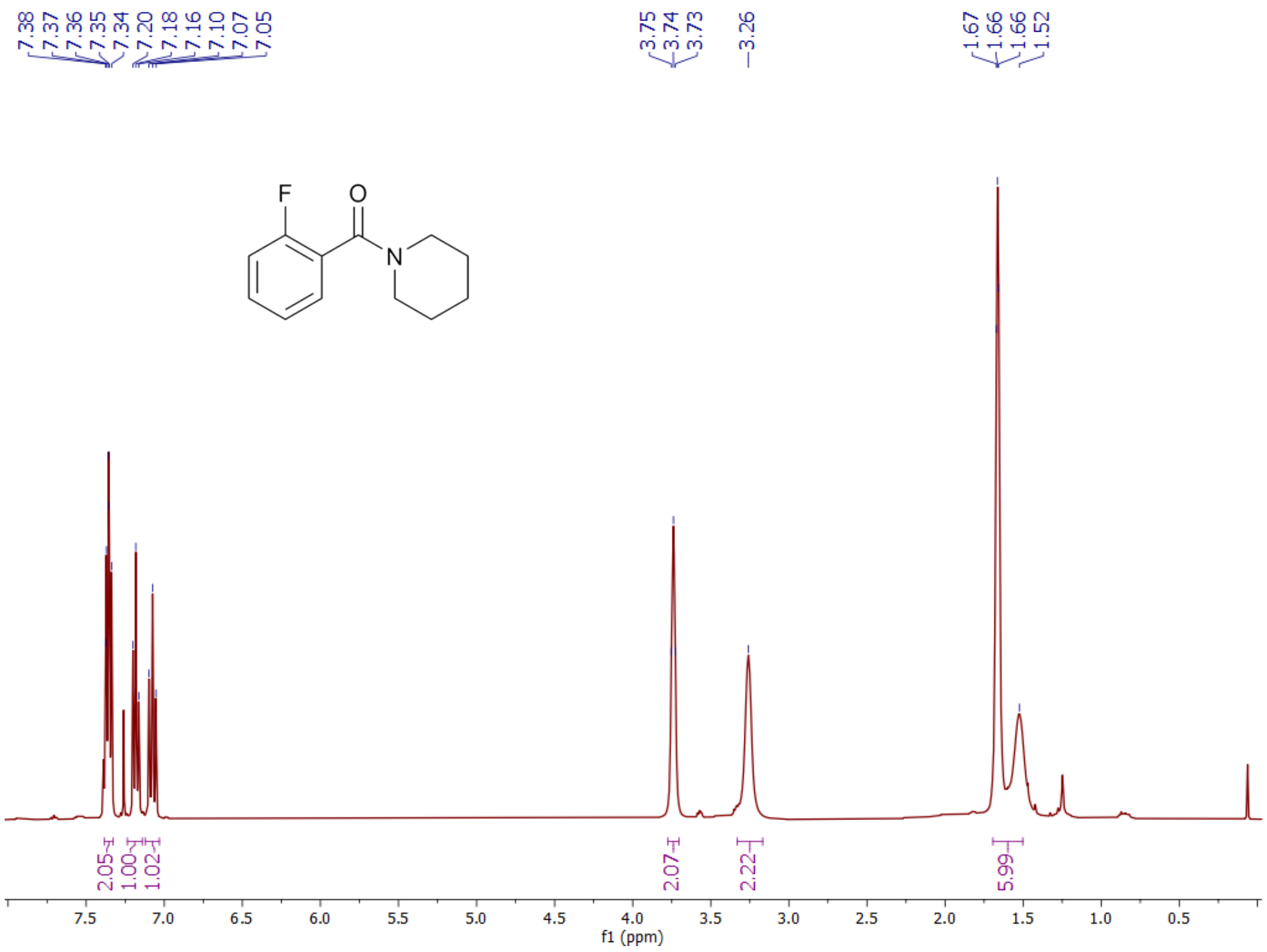

Figure S45. ^{1}H NMR spectrum of (2-Fluorophenyl)(1-piperidinyl)methanone (7)

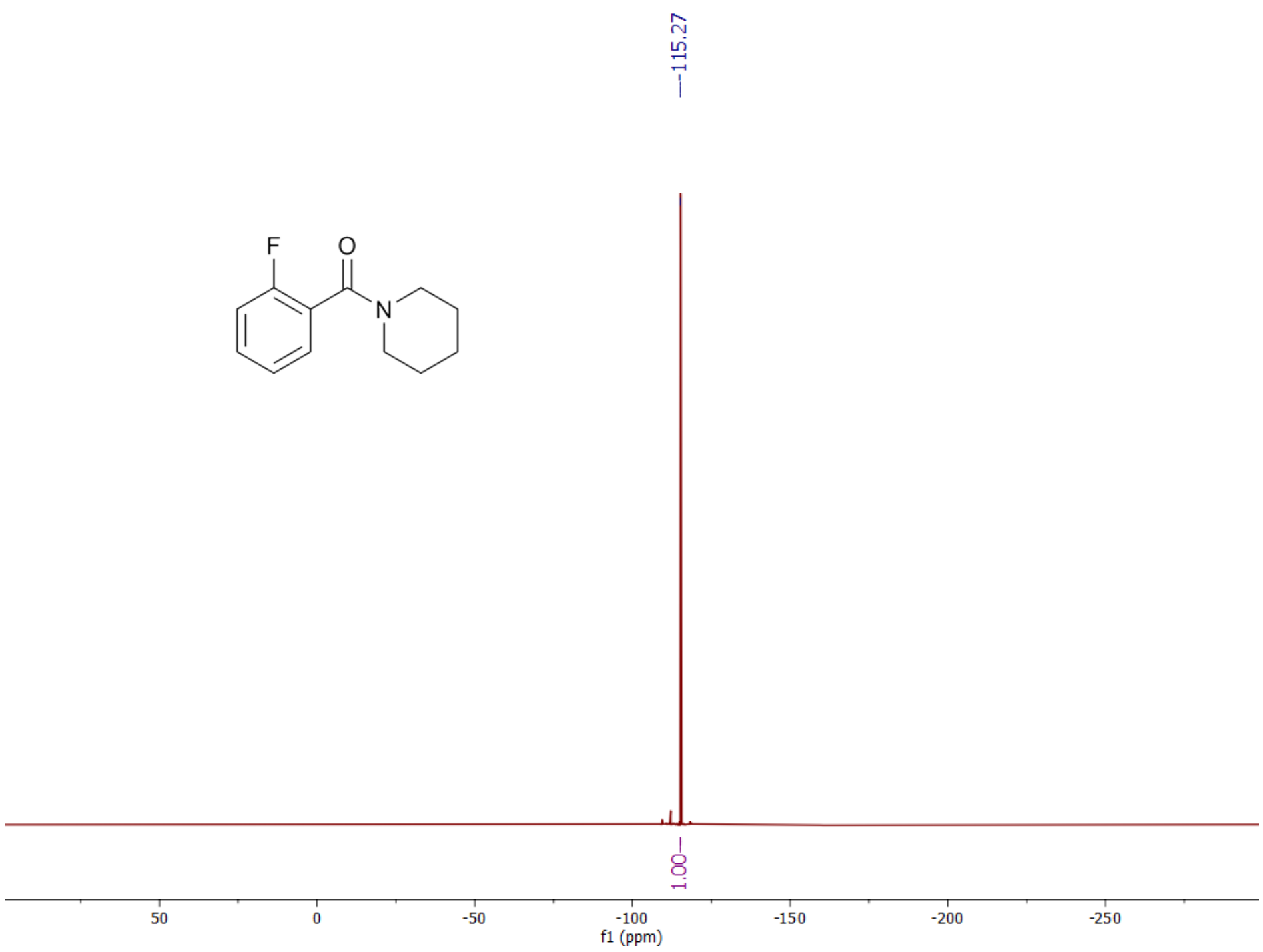

Figure S46. ^{19}F NMR spectrum of (2-Fluorophenyl)(1-piperidinyl)methanone (7)

1-Piperidinyl[4-(trifluoromethyl)phenyl]methanone (27): Yellow oil, 2.528 g (9.83 mmol) after five-hour collection (Yield: 78%). NMR signals are consistent with literature.[2] ^{1}H NMR (400 MHz, $CDCl_3$, δ): 7.66 (d, J = 8.0 Hz, 2H), 7.50 (d, J = 8.0 Hz, 2H), 3.72 (s, 2H), 3.30 (s, 2H), 1.60 (m, 6H). ^{19}F NMR (377 MHz, $CDCl_3$, δ): -62.84 (s, 1F).

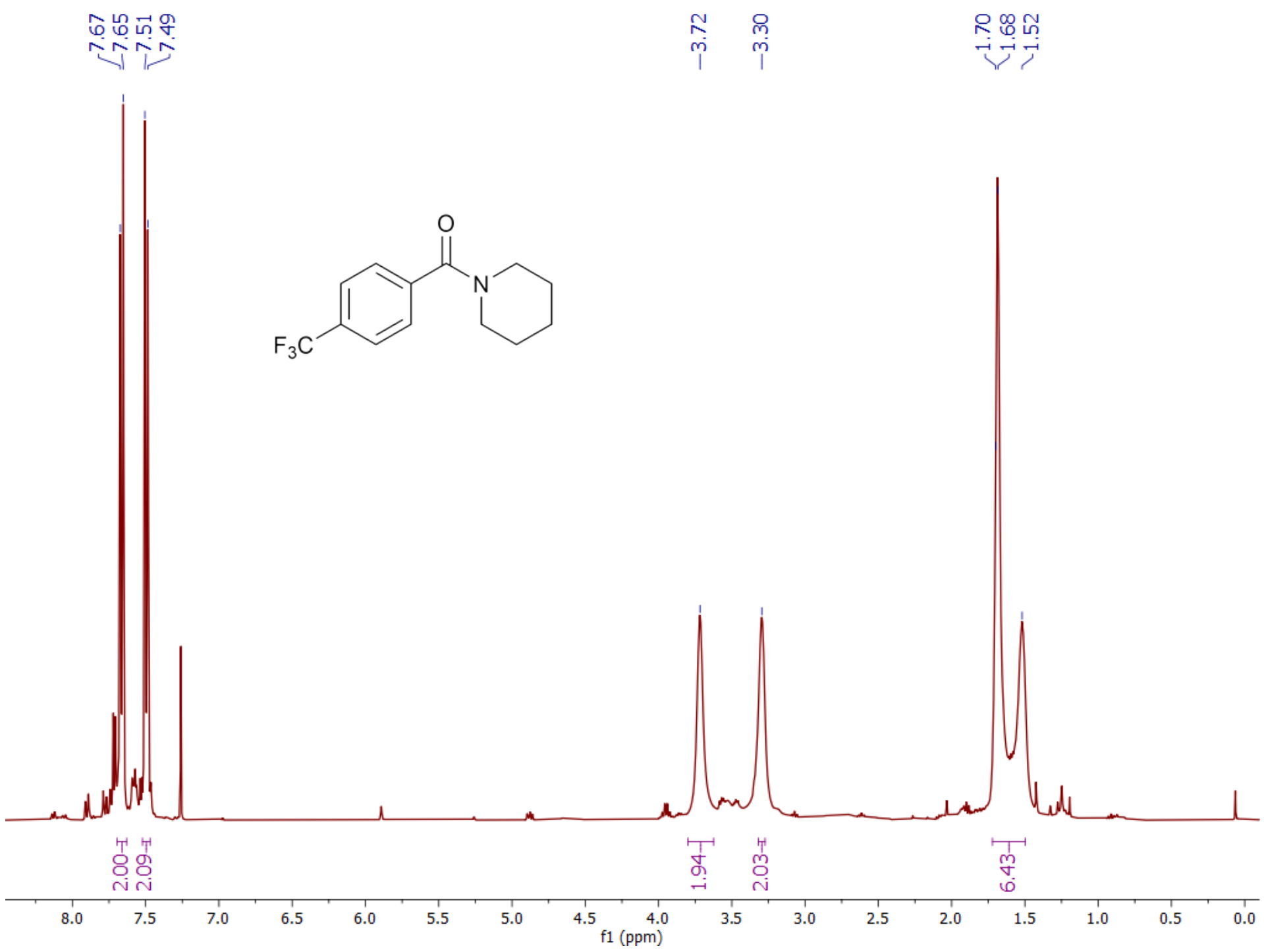

*Figure S47. ^{1}H NMR spectrum of 1-Piperidinyl[4-(trifluoromethyl)phenyl]methanone (**27**)*

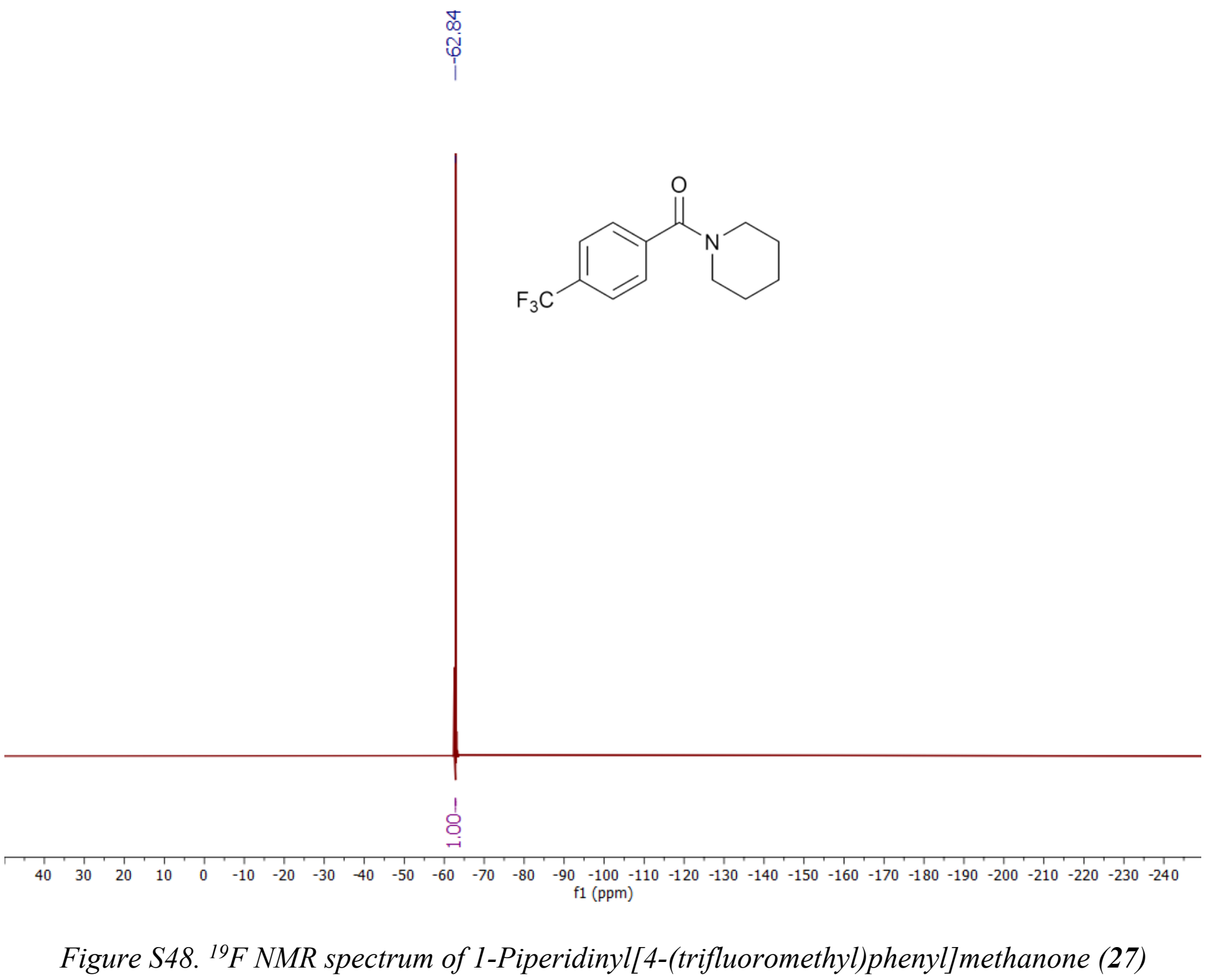

*Figure S48. ^{19}F NMR spectrum of 1-Piperidinyl[4-(trifluoromethyl)phenyl]methanone (**27**)*

[4-(5-Chloro-2-fluorobenzoyl)-1-piperazinyl](cyclopropyl)methanone (28): Further purification by column chromatography was necessary. Orange oil, 1.0796 g (3.47 mmol) after five-hour collection (Yield: 34 %). ^{1}H NMR (400 MHz, $CDCl_3$, δ): 7.43 – 7.34 (m, 2H), 7.07 (ddd, *J* = 8.6, 1.3 Hz, 1H), 3.98 – 3.53 (m, 6H), 3.36 (s, 2H), 1.74 (s, 1H), 1.01 (dt, *J* = 4.6, 3.2 Hz, 2H), 0.92 – 0.69 (m, 2H). ^{13}C NMR (101 MHz, $CDCl_3$, δ): 172.4, 164.0, 156.6 (d, *J* = 247.9 Hz), 131.7 (d, *J* = 8.2 Hz), 130.3 (d, *J* = 3.4 Hz), 129.2 (d, *J* = 3.9 Hz), 125.1 (d, *J* = 19.6 Hz), 117.4 (d, *J* = 23.5 Hz), 47.0, 45.2, 42.3 (2C), 11.1, 7.81 (4C). ^{19}F NMR (377 MHz, $CDCl_3$, δ): -62.84 (s, 1F). HRMS (ES-TOF): $^{m}/_{z}$ calc for $C_{15}H_{17}N_2O_2FCl$: 311.0963 [**28**+H]$^{+}$, found 311.0956.

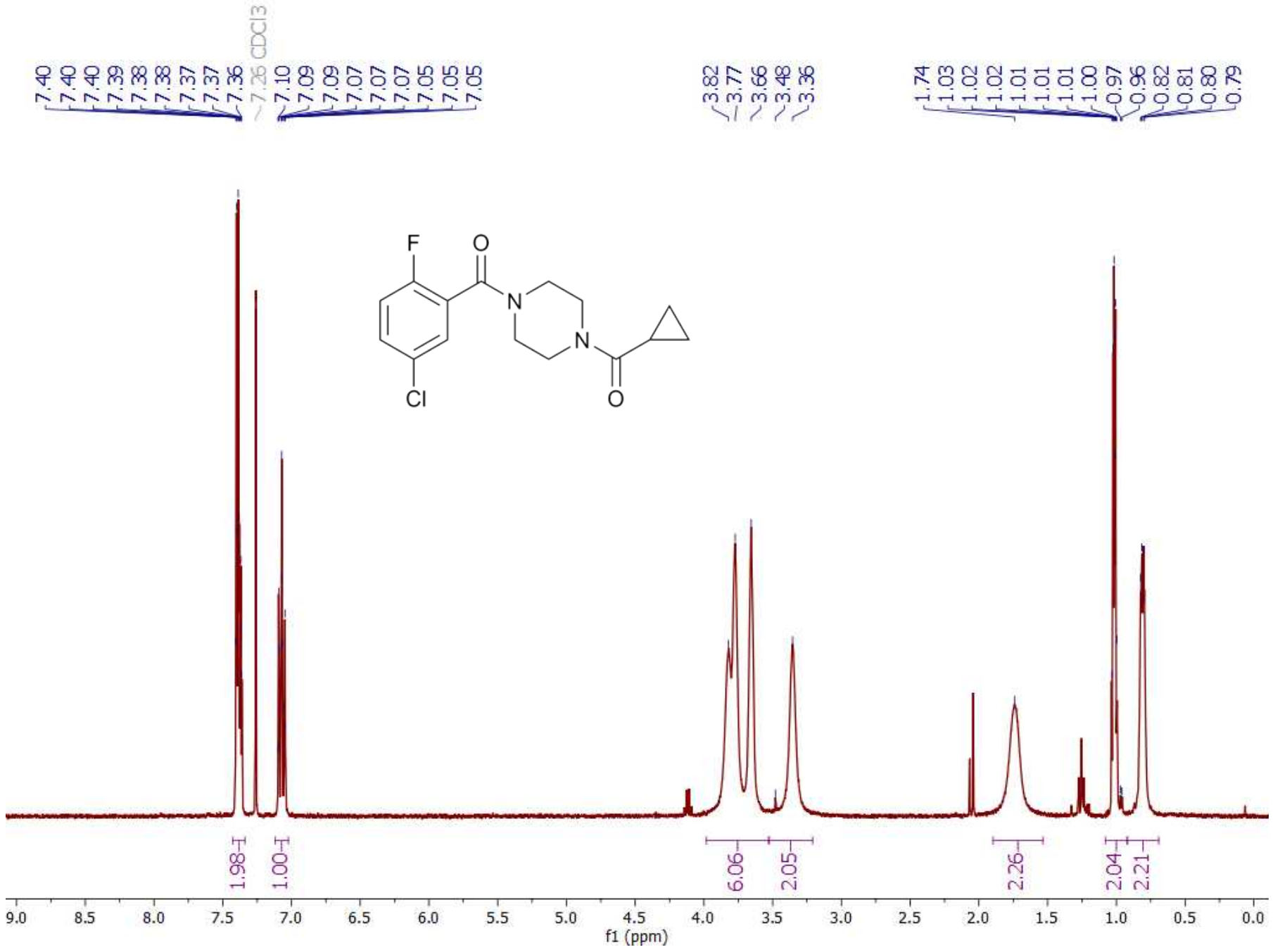

*Figure S49. ^{1}H NMR spectrum of [4-(5-Chloro-2-fluorobenzoyl)-1-piperazinyl](cyclopropyl)methanone (**28**)*

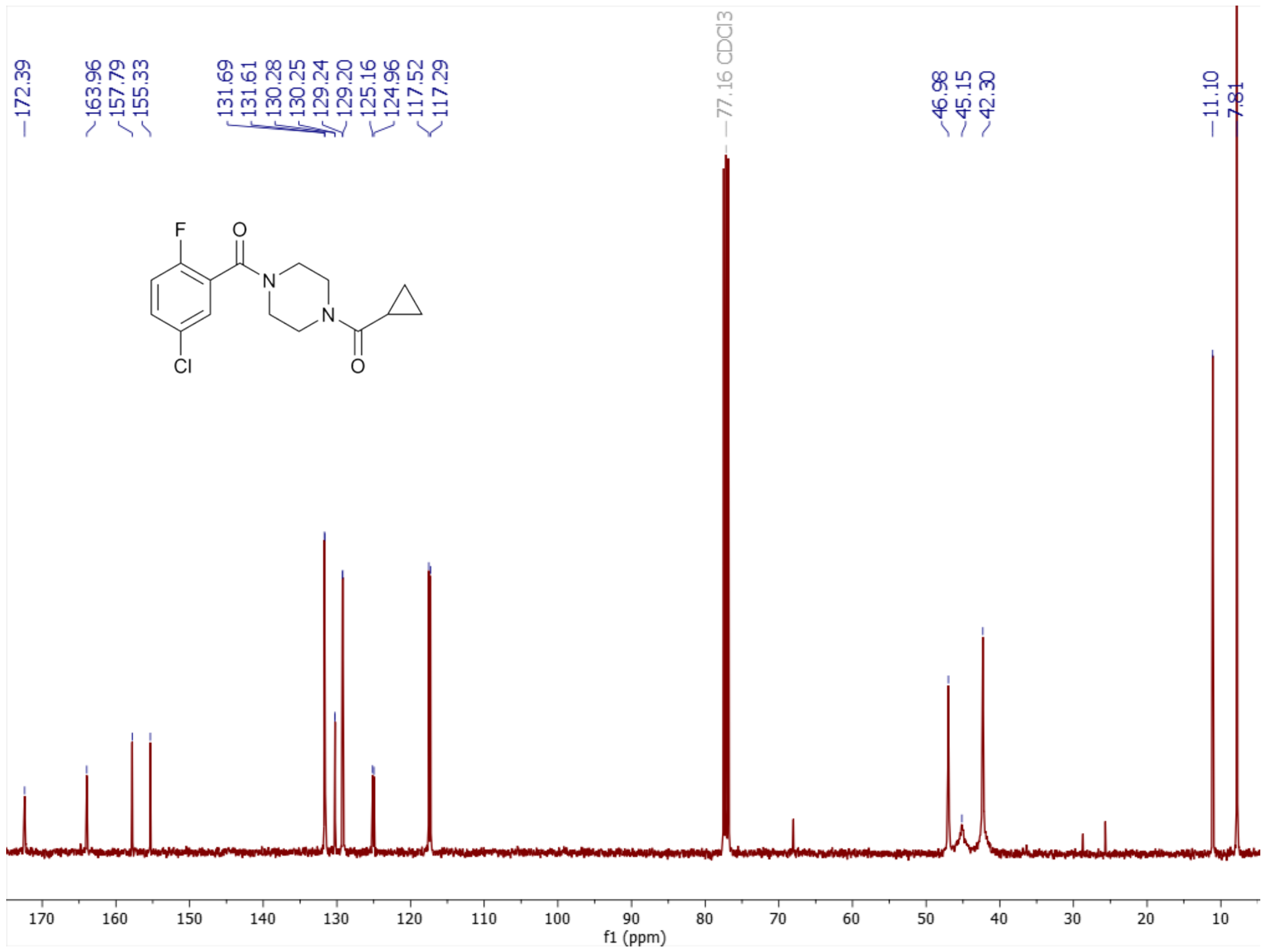

*Figure 50. ^{13}C NMR spectrum of [4-(5-Chloro-2-fluorobenzoyl)-1-piperazinyl](cyclopropyl)methanone (**28**)*

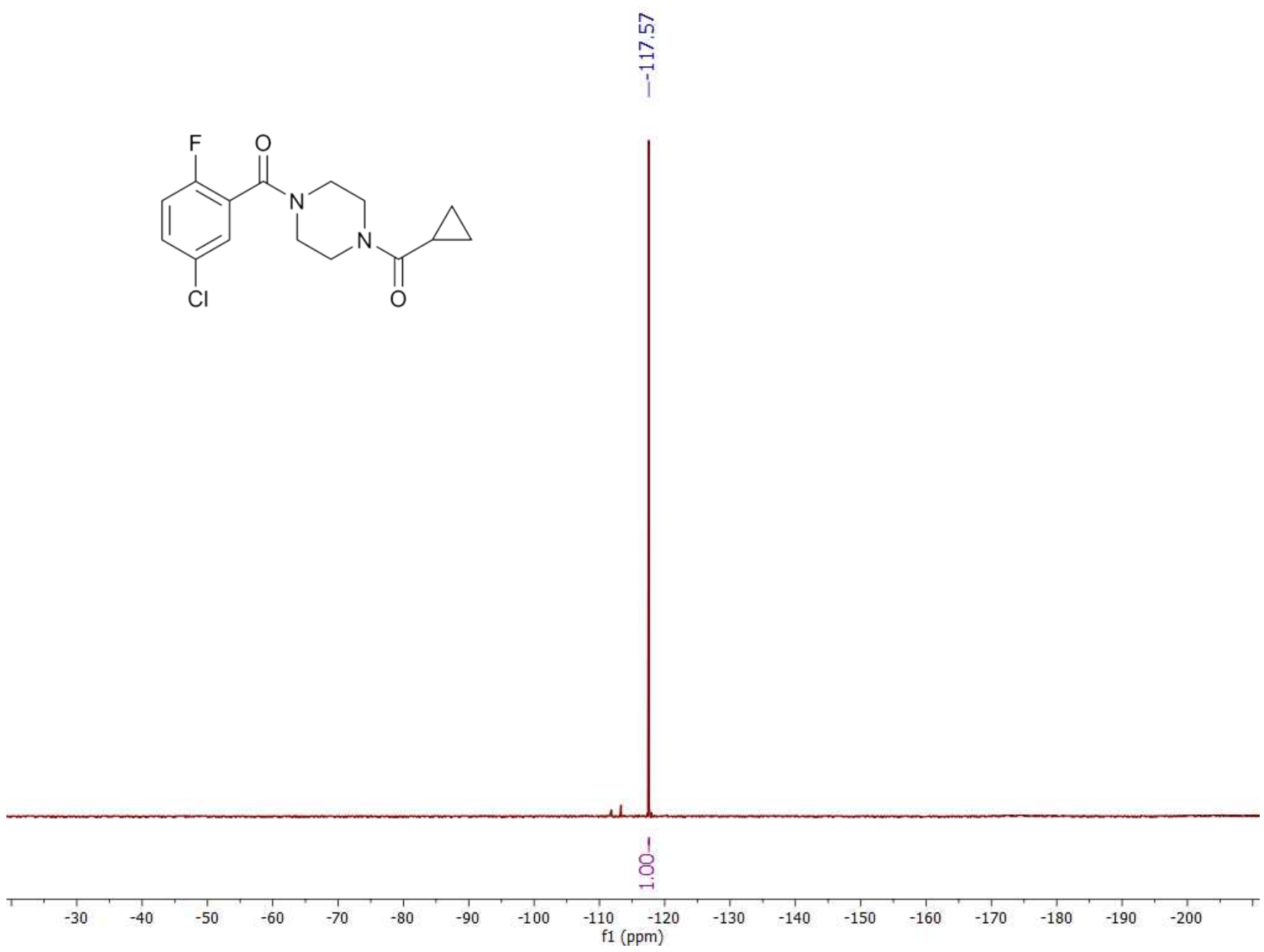

*Figure S51. ^{19}F NMR spectrum of [4-(5-Chloro-2-fluorobenzoyl) -1-piperazinyl](cyclopropyl)methanone (**28**)*

4-Chloro-N-(2-morpholinoethyl)benzamide (Moclobemide, 29): Further purification by column chromatography was necessary. White solid, 0.1791 g (3.47 mmol) after 2.5-hour collection (Yield: 11 %). NMR signals are consistent with literature.[21] ^{1}H NMR (400 MHz, $CDCl_3$, δ): 7.79 – 7.71 (m, 2H), 7.45 – 7.39 (m, 2H), 6.99 (s, 1H), 3.78 (t, J = 4.7 Hz, 4H), 3.59 (q, J = 5.4 Hz, 2H), 2.68 (t, J = 5.8 Hz, 2H), 2.59 (s, 4H).

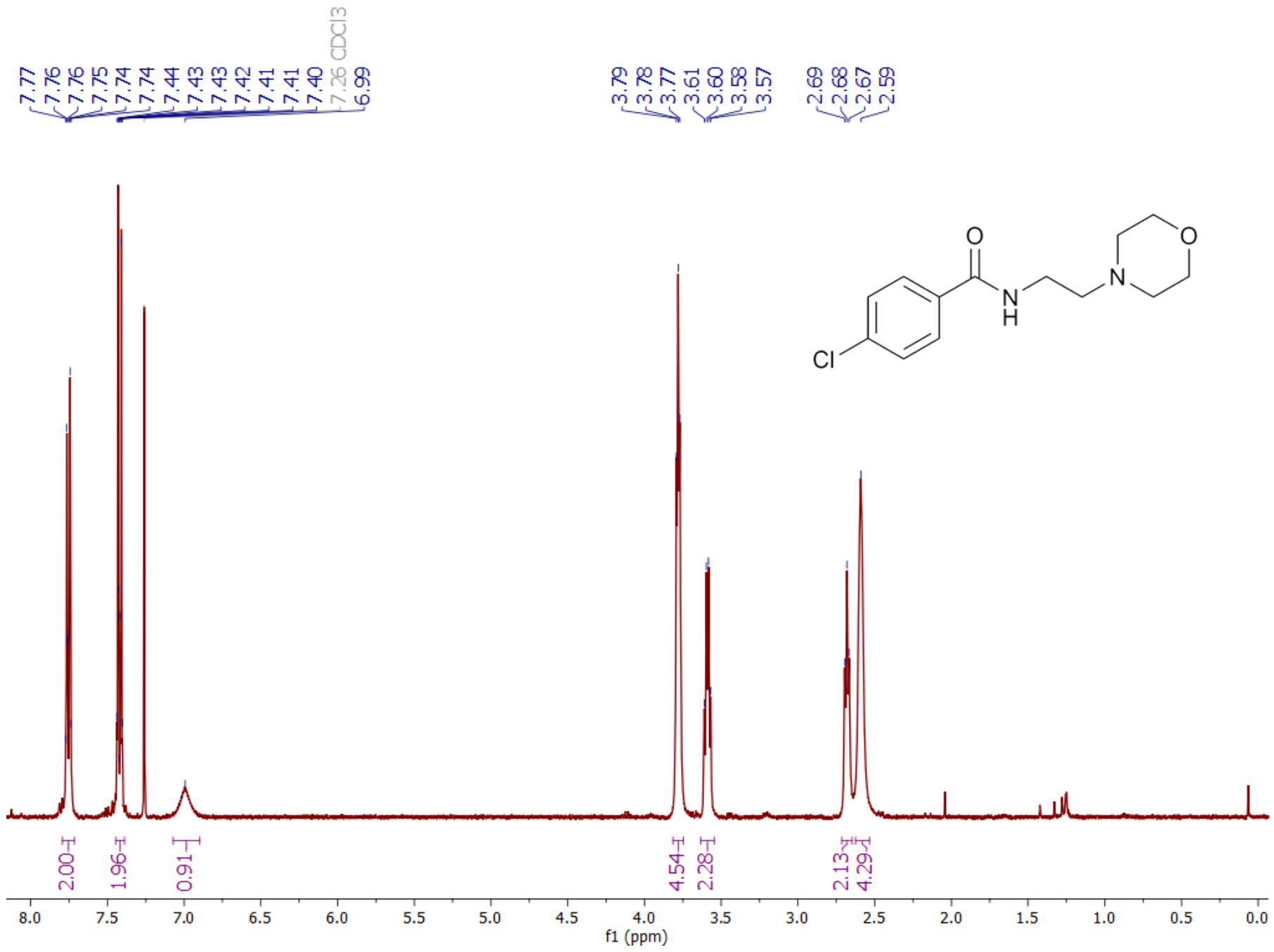

*Figure S52. ^{1}H NMR spectrum of 4-Chloro-N-(2-morpholinoethyl)benzamide (**29**)*

8. Actinometry

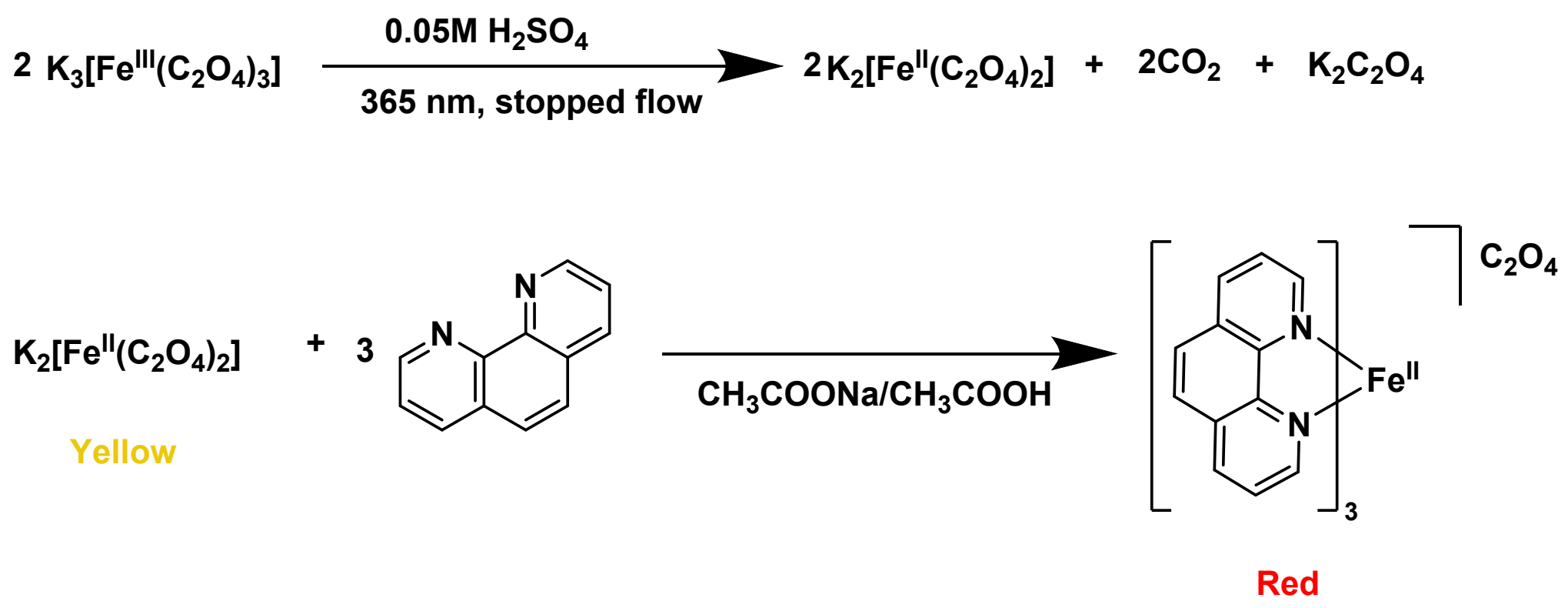

A general procedure of the ferrioxalate actinometry was adapted from literature to measure the photon flux of selected reactors.[22-24] Potassium ferrioxalate and 1,10-phenanthroline were used directly from suppliers, and distilled water was used as solvent. A solution of 0.1 M potassium ferrioxalate in 0.05 M sulfuric acid was prepared in a dark room and protected from ambient light using foil covered bottles.

Vapourtec UV-150 photoreactor: The reactor was coupled with the 365 nm 60 W LEDs. The ferrioxalate solution was pumped into the reactor coil (10 ml), while the LED lights were off. After the whole system was filled with fresh solution, the flow was stopped, and the reactor was treated as a batch system (stopped flow measurements). The lights were turn on for a specific time (1, 2, 3 and 4 seconds), and the solution was then pumped out of the reactor in the dark and collected into vials. Each time was measured three times, and a sample was collected under dark conditions by flowing ferrioxalate solution through the reactor.

After collection, 0.1 mL of every sample was transferred into vials containing pre-measured stock solution of phenanthroline (10 mL buffer solution containing 0.003 M of 1,10-phenanthroline in 0.05 M sulfuric acid and 0.03 M sodium acetate). Mixed samples were left in the dark for 1 hour, before measuring the UV-Vis absorption spectra. The concentration of Fe^{2+} was determined by analysing the absorption value at 550 nm versus a calibration curve developed prior (0.03 – 2 mM solutions of Fe_2SO_4 in the 1,10-phenanthroline buffer solution, Figure S53). Photon flux of the 365 nm LEDs in the 10 ml coil of the Vapourtec UV-150 photoreactor was determined to be 2.80E-5 ± 0.07E-5 mol/s (Figure S54).

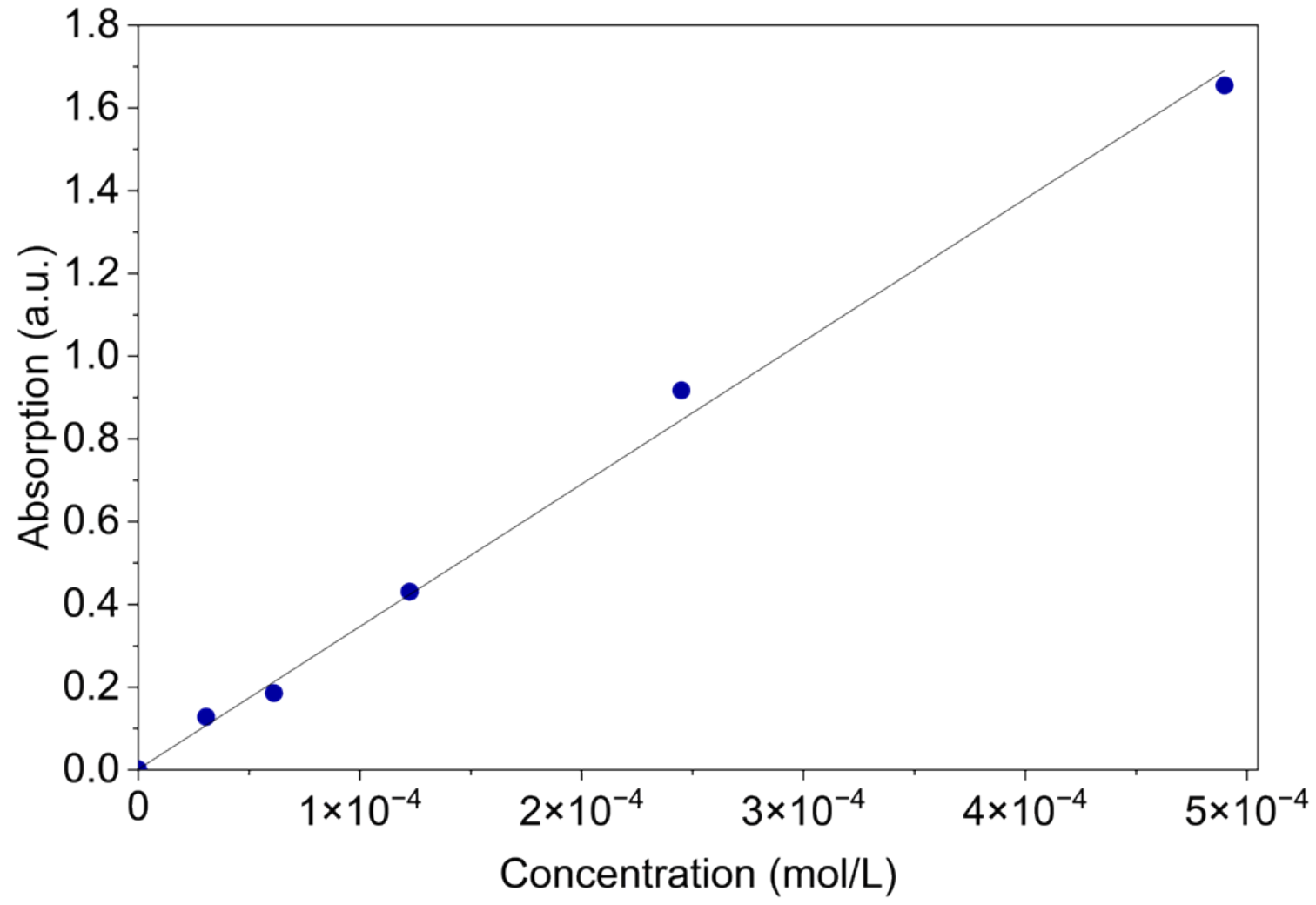

Figure S53. UV-vis spectroscopy calibration curve for photon flux determination

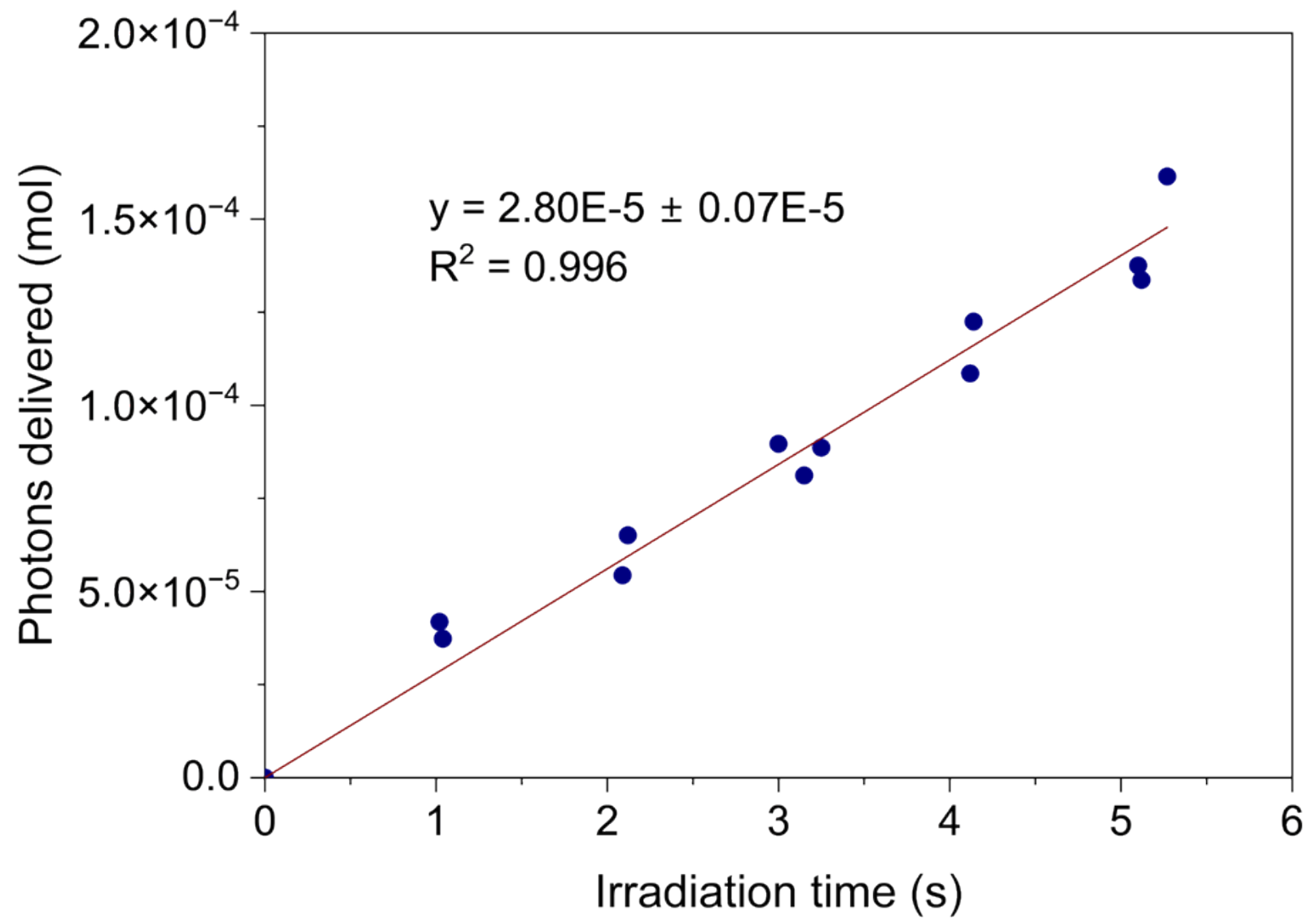

Figure S54. Actinometry quantification of light delivery for the Vapourtec UV-150 photoreactor with 10 ml coil and 365 nm 60 W LEDs.

9. Bibliography

1. R. Kohlbecher and T. J. J. Müller, *Chem. Eur. J.*, 2024, **30**, e202304119.
2. M. Annereau, M. Salmain and V. Corcé, *ChemComm*, 2024, **60**, 3934-3937.
3. C. Laurence, S. Mansour, D. Vuluga and J. Legros, *J. Phys. Org. Chem.*, 2025, **38**, e4665.
4. H. Tafazolian, D. C. Samblanet and J. A. R. Schmidt, *Organometallics*, 2015, **34**, 1809-1817.
5. Z. Huang, H. Xu, W. Zhang, Z. Liang, P. Lin, L. Zhu and X. Yao, *Eur. J. Org. Chem.*, 2025, **28**, e202401348.
6. Y. Yang, J. Liu, F. S. Kamounah, G. Ciancaleoni and J.-W. Lee, *J. Org. Chem.*, 2021, **86**, 16867-16881.
7. C. Liu, H.-N. Chen, T.-F. Xiao, X.-Q. Hu, P.-F. Xu and G.-Q. Xu, *ChemComm*, 2023, **59**, 2003-2006.
8. W. Fengtian, Y. Fangming, Z. Rong, S. Yijia, L. Xiuping, Z. Caihong, C. Yenan, W. Ling and C. Chunna, *Lett. Org. Chem.*, 2023, **20**, 221-229.
9. J. Park, S. Park, G. S. Jang, R. H. Kim, J. Jung and S. K. Woo, *ChemComm*, 2021, **57**, 9995-9998.
10. X.-Q. Ning, S.-J. Lou, Y.-J. Mao, Z.-Y. Xu and D.-Q. Xu, *Org. Lett.*, 2018, **20**, 2445-2448.
11. Z. Tang, Z. Yao, Y. Yu, J. Huang, X. Ma, X. Zhao, Z. Chang and D. Zhao, *Angew. Chem. Int. Ed.*, 2024, **63**, e202412152.
12. E. Bellamy, O. Bayh, C. Hoarau, F. Trécourt, G. Quéguiner and F. Marsais, *ChemComm*, 2010, **46**, 7043-7045.
13. D. Leow, *Org. Lett.*, 2014, **16**, 5812-5815.
14. Y. Yan, P. Wang, S. Li, J. Dong, G. Li, C. Wang and D. Xue, *Org. Lett.*, 2025, **27**, 675-679.
15. L. Wang, M. Yu, C. Wu, N. Deng, C. Wang and X. Yao, *Adv. Synth. Catal.*, 2016, **358**, 2631-2641.
16. Z. Tang, K. Mo, X. Ma, J. Huang and D. Zhao, *Angew. Chem. Int. Ed.*, 2022, **61**, e202208089.
17. J. Liu, C. Shao, Y. Zhang, G. Shi and S. Pan, *Org. Biomol. Chem.*, 2014, **12**, 2637-2640.
18. A. Nagaki, Y. Uesugi, H. Kim and J.-i. Yoshida, *Chem. Asian J.*, 2013, **8**, 705-708.
19. Y. Man and B. Xu, *Org. Lett.*, 2024, **26**, 2456-2461.
20. M. Mondal and A. Saha, *J. Org. Chem.*, 2025, **90**, 52-58.
21. B. Zoller, J. Zapp and P. H. Huy, *Chem. Eur. J.*, 2020, **26**, 9632-9638.
22. B. Wriedt and D. Ziegenbalg, *J. Flow Chem.*, 2020, **10**, 295-306.
23. B. Wriedt and D. Ziegenbalg, *ChemPhotoChem*, 2021, **5**, 947-956.
24. T. Aillet, K. Loubiere, O. Dechy-Cabaret and L. Prat, *Int. J. Chem. React. Eng.*, 2014, **12**, 257-269.
